# Effects of music therapy as an adjunct to chest physiotherapy in children with cystic fibrosis: A randomized controlled trial

**DOI:** 10.1371/journal.pone.0241334

**Published:** 2020-10-30

**Authors:** Alberto Montero-Ruiz, Laura A. Fuentes, Estela Pérez Ruiz, Nuria García-Agua Soler, Francisca Rius-Diaz, Pilar Caro Aguilera, Javier Pérez Frías, Elisa Martín-Montañez

**Affiliations:** 1 Departamento de Farmacología y Pediatría, Universidad de Málaga, Instituto de Investigación Biomédica de Málaga, Facultad de Medicina, Málaga, Spain; 2 Consejería de Educación, Junta de Andalucía, Delegación Territorial de Málaga, Málaga, Spain; 3 Hospital Regional Universitario de Málaga, Sección de Neumología Pediátrica, Málaga, Spain; 4 Departamento de Medicina Preventiva y Salud Pública, Universidad de Málaga, Facultad de Medicina, Málaga, Spain; PLOS ONE, UNITED STATES

## Abstract

Airway clearance therapy (ACT) is considered an important approach to improve airway clearance in children with cystic fibrosis (CF). Daily ACT administration requires substantial commitments of time and energy that complicate ACT and reduce its benefits. It is crucial to establish ACT as a positive routine. Music therapy (MT) is an aspect of integrative strategies to ameliorate the psycho-emotional consequences of chronic diseases, and a MT intervention could help children with CF between the ages of 2 and 17 develop a positive response. The aim of this randomized controlled trial was to evaluate the effects of specifically composed and recorded instrumental music as an adjunct to ACT. We compared the use of specifically composed music (Treated Group, TG), music that the patient liked (Placebo Group, PG), and no music (Control Group, CG) during the usual ACT routine in children with CF aged from 2 to 17. The primary outcomes, i.e., enjoyment and perception of time, were evaluated via validated questionnaires. The secondary outcome, i.e., efficiency, was evaluated in terms of avoided healthcare resources. Enjoyment increased after the use of the specifically composed music (children +0.9 units/parents +1.7 units; p<0.05) compared to enjoyment with no music (0 units) and familiar music (+0.5 units). Perception of time was 11.1 min (±3.9) less than the actual time in the TG (p<0.05), 3.9 min (±4.2) more than the actual time in the PG and unchanged in the CG. The potential cost saving related to respiratory exacerbations was €6,704.87, while the cost increased to €33,524.35 in the CG and to €13,409.74 in the PG. In conclusion, the specifically composed, played and compiled instrumental recorded music is an effective adjunct to ACT to establish a positive response and is an efficient option in terms of avoided costs.

Trial registered as ISRCTN11161411. ISRCTN registry (www.isrctn.com).

## Introduction

Cystic fibrosis (CF) is an inherited autosomal recessive multisystemic disease characterized mainly by respiratory and digestive disorders [1,2]. Due to a defect in the CF transmembrane conductance regulator gene, excess dehydrated and thick secretions are produced in the lungs, liver, pancreas, intestine, sweat and salivary glands and reproductive organs [3]. The cause of morbi-mortality is linked to respiratory manifestations due to the accumulation of mucus in the airways, which ultimately leads to secondary infections, bronchiectasis, and respiratory failure [2]. Routine airway clearance therapy (ACT) is one of the main approaches used to improve airway clearance [4,5]. These airway clearance techniques are prescribed daily and, in the case of children, are to be given by their parents until children and adolescents are old enough to be actively involved in their own ACT. Therefore, ACT is well established as an integral part of the management of CF and is aimed at minimizing symptoms, reducing the frequency of exacerbations and improving lung function [5]. However, ACT requires substantial commitments of time and energy, leading to low levels of ACT adherence [6] and decreases in its benefits [7].

Individuals with CF and their parents report elevated symptoms of psychological burden [8]. To avoid these negative effects related to this daily routine, it is important to establish ACT as an enjoyable task, and a music therapy (MT) strategy could help people with CF develop a positive response. In pulmonary diseases, MT interventions are components of integrative strategies adopted to ameliorate physical or psycho-emotional consequences [9,10]; however, MT interventions in CF are infrequent, and in any case very few. To our knowledge, only five studies have evaluated the effects of music-based strategies, based on playing instruments [11], singing [12,13] or listening to music [14,15], as adjunct therapy in children with CF. In this sense, it has been shown that listening to carefully selected motivational music while walking, which was chosen by physiotherapists, can lead to a positive affective response during exercise, increasing the enjoyment of exercise in CF patients between the ages of 8 and 18 [15]. Furthermore, a study carried out in children with CF who were less than 24 months old indicated that listening to specifically composed and recorded music as an adjunct to ACT increased children’s and parents’ enjoyment of ACT and assisted in the establishment of a positive routine [14]. Although Calik-Kutukcu and colleagues [15] showed that listening to selected motivational music can have a positive effect on exercise, Grasso and colleagues [14] revealed that listening to recorded music specifically composed, is an effective mean to increase the positive effects found during ACT routine with toddler, being more effective than familiar music. We planned this clinical trial considering the importance of these specific characteristics to enhance the positive effect of music [14] and also, the lack of literature on the use of music during ACT with CF children older than 24 months old. Thus, the purpose of the current study was to evaluate the effects of listening to specifically composed, played and recorded music as an adjunct to ACT in children with CF older than 2 years old. We hypothesized that the use of the specifically composed music may provide benefits compare to patients-preferred music or the use of no music. The primary aims were to examine (1) a possible increase in patients’ and parents’ enjoyment of the ACT routine and (2) a possible decrease in perception of time taken to complete the routine. As a secondary aim, we wanted to analyze the efficiency of the music therapy intervention in terms of avoided healthcare resources.

## Materials and methods

### Study participants

Individuals with CF between the ages of 2 and 17 were recruited from the Pediatric Pulmonology Unit at Malaga Regional Hospital, which was providing care to 80 people with CF at the time of study. Participants who met all of the inclusion criteria, none of the exclusion criteria (Table 1) and provided written informed consent were included in the trial. Due to all the participants were minors, written informed consent was obtained from their parents, which was also signed by children aged > 12.

**Table 1:** Clinical trial inclusion and exclusion criteria.

**Inclusion criteria**	Participants diagnosed with CF based on international criteria [16]: a positive sweat test (chloride value ≥ 60 mmol/L); 2 CF-causing CFTR mutations; and clinical features compatible with CF
	Participants undergoing periodic clinic visits in the CF unit
	Participants in the target age range
	Participants with an understanding of the purpose of the study
**Exclusion criteria**	Participants without ACT prescription
	Participants with severe hearing loss
	Participants with clinical complications such that the ACT may have to be adapted or may be contraindicated: radiologic or clinical risk of pneumothorax or pneumomediastinum; barotrauma in the month prior to entry in the study; or past history of massive or life-threatening hemoptysis [17]
	Transplant recipients or patients awaiting a lung transplant

Abbreviations: CF, cystic fibrosis; CFTR, cystic fibrosis transmembrane conductance regulator; ACT, airway clearance therapy.

Information about the clinical variables (age of diagnosis, body mass index, Bhalla score and respiratory infection exacerbations) was obtained from the clinical center.

The study was conducted according to the Declaration of Helsinki and was approved by the Ethics in Human Research Committee of Malaga Regional Hospital on October 27, 2016. The authors confirm that all ongoing and related trials for this intervention are registered. We registered the trial on the ISRCTN registry (ISRCTN11161411: www.isrctn.com.) on June 15, 2019. Patient recruitment and follow-up were conducted from February 1, 2018 to July 31, 2018. The delay in registering this study in a publicly accessible register was because the trial was previously registered in the Andalusian Ethics Biomedical Research Registry (PEIBA) in October 2016. Thus, the trial was registered before recruiting the first participant in the study. We thought the PEIBA register was included in the WHO list of approved registries, but in June 2019, we realized that the PEIBA registry was not a publicly accessible registry; therefore, we decided to register the trial in the ISRCTN registry immediately.

### Study design

The MT intervention evaluated consisted of the use of instrumental music that was specifically composed, played and compiled for children with CF and that was used as an adjunct to each part of the ACT routine without modifying the usual treatment regimens or programmed clinic visits; therefore, there was no risk of harm. We compared the use of this specifically composed music with (1) the use of music the patients liked, also called familiar music, and (2) the use of no music during ACT management. The participants were randomly allocated using the ‘Subjects assignment to treatment’ module of the software for the epidemiologic analysis of tabulated data Epidat (version 3.1) [18]. A design for equal-sized groups was chosen. After we set the number of groups (n = 3) and the total sample size (n = 54), Epidat made a random assignment according to the participant order number, with each participant assigned to one of the 3 following conditions: (1) Treated Group (TG)–use of the specifically composed music as an adjunct to the ACT routine; (2) Placebo Group (PG)–use of music the patients liked as an adjunct to the ACT routine; and (3) Control Group (CG)–the standard practice of the ACT routine.

In our Pediatric Pulmonology Unit, ACT is divided into 3 sections: nebulizer inhalation treatment, airway clearance techniques and relaxation/antibiotic nebulizer treatment, if necessary. Participants in the TG were given a music CD with music that was composed, played and compiled specifically for children with CF by a professional musician and music teacher under the recommendations of the CF specialists at the Pediatric Pulmonology Unit. The recommendations provided for developing the music were (1) to encourage enjoyment; (2) to elicit relaxation, maintaining children’s interest to facilitate deep inhalation during the inhaled treatment sections; (3) to provide appropriate rhythmic support for the management of airway clearance techniques to improve mucus clearance; and (4) to promote distraction from the time spent on the routine. The CD consisted of instrumental composed music played on percussion instruments to use as an adjunct to ACT during the 3-month trial period. The music CD had 3 sections related to the ACT routine: section A for the nebulizer treatment, section B for ACT work and bronchial clearance and section C for relaxation and nebulization. The CD was 40 min long, corresponding to the average length of the ACT routine. Section A contained 4 slow songs (≤ 65 bpm) with a total of 13 min of relaxing music for the duration of the nebulizer inhalation treatment. Section B consisted of 5 moderate rhythmical songs (mean = 112.4 bpm) and was a total of 23 min long; it provided a rhythmic structure to support the management of airway clearance techniques such as huffing, coughing, percussions or vibrations. Section C was a 4-min-long relaxing song that was a variation of a section A song. The music was performed with pitched percussion instruments (marimba, vibes, glockenspiel and xylophone) and unpitched percussion instruments (drums, congas, bongos, multipercussion set and small percussion instruments) that are widely used in MT interventions [19,20]. MuseScore software was used to write the scores, and the software Audacity was used to record and edit the audio. The music was compiled on a CD. Participants in the PG were given recommendations for the use of familiar music as an adjunct to ACT during the 3-month trial period. The recommendations encouraged the patients to listen to slow, relaxing music that they liked during the first and last ACT sections, and moderate rhythmical songs in the middle section. The CG was composed of participants who were asked to continue with their usual ACT routine during the 3-month trial period. Participants in these 2 groups (PG and CG) were given the music CD to keep at the end of the study.

Questionnaires designed and validated by Grasso and colleagues were employed in the current study [14]. We evaluated the internal consistency by calculating Cronbach's alpha coefficient among the items referring to the ‘enjoyment’ construct in an independent sample of people with CF over 18 and their parents [14] (Cronbach's alpha = 0.856).

Participants completed a baseline questionnaire, a follow-up questionnaire and an evaluation questionnaire during the 3-month trial period. The baseline questionnaire was completed in a face-to-face interview immediately following recruitment during a routine clinic visit. The follow-up and the evaluation questionnaires were completed via telephone interviews 6 and 12 weeks after the first interview, respectively. We used the middle questionnaire collecting information about experience with physiotherapy routine as a follow-up strategy, instead of evaluating the intervention. Nonclinical research staff performed the interviews and was trained previously. Adolescent participants completed questionnaires independently and were given the option to have their parents join them for the interviews. In all cases, the parents of adolescents joined. After the baseline questionnaire, participants received a reference sheet containing the Likert-type response scales and a list of descriptive words for reference during the telephone interviews. This reference sheet was also posted on the MT intervention website (an additional support for participants, which included a summary of the intervention, the reference sheet and contact information).

### Outcome measures

The primary outcomes, i.e., enjoyment and perception of time, were evaluated via the validated questionnaires specified above [14]. Enjoyment was assessed on 7-point Likert-type scales completed by participants, where +3 was the most enjoyment, 0 was neutral and −3 was the least enjoyment. Participants also chose 3 descriptive words from a list of 12 words that best described their own experiences of ACT. Parents ranked their own enjoyment and their children’s enjoyment on these scales when the children were under 8 years old. Parents also chose the 3 descriptive words in these cases.

Perception of time was assessed as the difference between the actual time taken to complete ACT (measured using a stopwatch) and the apparent time to complete ACT (subjective perception) [14]. The actual time was evaluated as the response (given in minutes) to the question ‘How long does the ACT routine take?’ The apparent time was evaluated as the response (given in minutes) to the question ‘How long does it feel like it takes to complete ACT?’.

The secondary outcome, i.e., efficiency of the intervention, was evaluated in terms of avoided healthcare resources to treat respiratory infection exacerbations that required hospitalization. The number of exacerbations and the number of days hospitalized during the previous 3 months and during the 3-month trial period were obtained from the clinic history. Hospitalization cost (expressed in Euros) was estimated based on the associated diagnosis-related group (DRG) (DRG 131: CF-Pulmonary disease, average hospitalization: 11.14 days) [21]. The DGR is a unit for classifying patients by diagnosis, average length of hospital stay and therapy received.

### Statistical analysis

To calculate the sample size, the perception of time taken to complete ACT was used as the main variable. In the study by Grasso [14], the control group values for this variable were 0.2 min ±10.2 (mean ± SD); based on an assumption of these values as a baseline, a power of 80%, a confidence level of 95%, and a similar variability at the end of the study, a difference of 8 min would be considered statistically significant with a sample size of 13 participants. A total of 39 participants are necessary to enable a valid answer to the research question. The variables are expressed as the mean (±SD) or n (%). To compare quantitative measures, ANOVA/Kruskal-Wallis tests were used, while chi-square test was used for the qualitative demographic variable ‘gender’. To compare the differences in the change in the variables between the different groups, from a global point of view, a repeated measures multivariate analysis of variance (MANOVA) test was applied with a between-participants factor (different groups) and a within-participants factor (different time points of data collection). Depending on compliance with the assumption of sphericity, Greenhouse-Geisser correction was used. Box test and Levene test were used to ensure homogeneity of covariance matrices and error variances, respectively. To more specifically compare the differences between the baseline and final measures in each group, the Wilcoxon test was applied. To compare the response to the use of music after the intervention in the TG and PG, the Mann–Whitney U test was used. Statistical significance was declared at the 0.05 level. Statistical analysis was performed using SPSS software (version 24.0; IBM SPSS Statistics, Chicago, IL).

## Results

**Fig 1:**
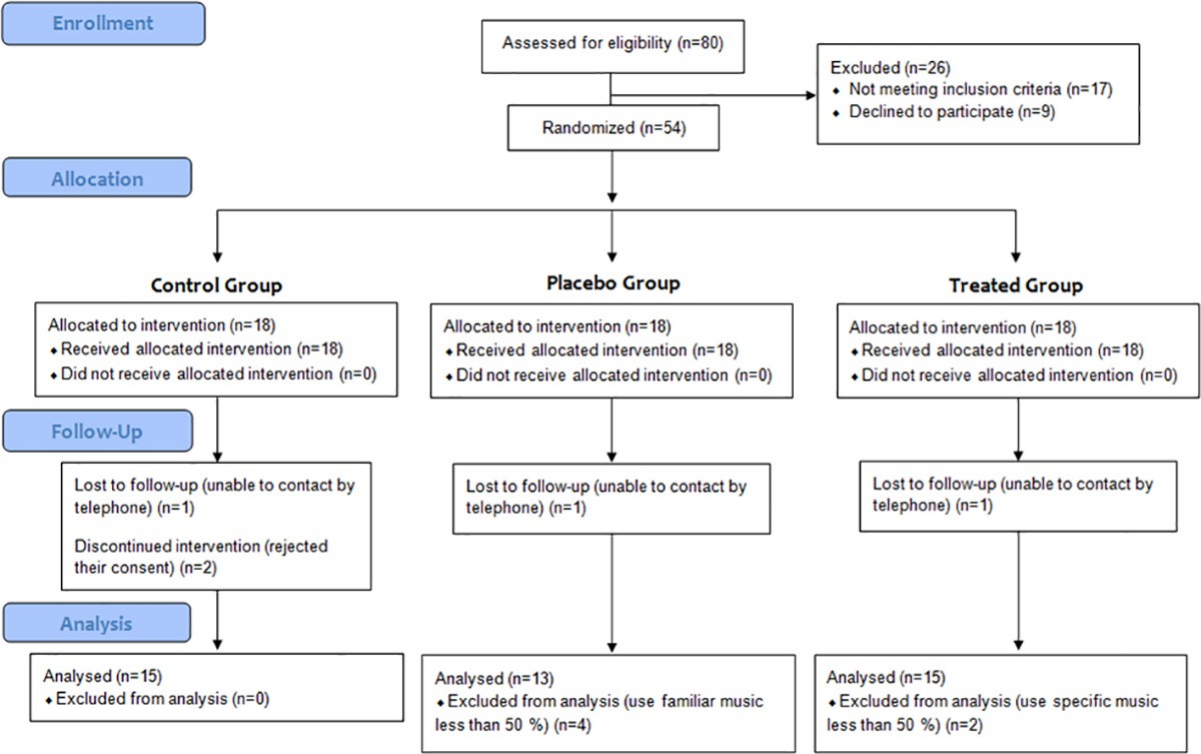
Flow diagram of the clinical trial.

A total of 54 patients met all the inclusion criteria and agreed to participate in the intervention. As we explain in the study design, the participants were randomly allocated into the TG (n = 18), PG (n = 18) and CG (n = 18). Eleven patients were withdrawn when they could not be contacted by telephone to be interviewed (n = 3), rejected their consent (n = 2) or used the music CD (n = 2) or familiar music (n = 4) as an adjunct the ACT less than 50% of the time, leaving a total of 15 patients in the TG, 15 patients in the CG, and 13 patients in the PG (Fig 1). The percentage of participants who withdrew from the study (20.4%) was lower than the potential withdrawal estimated for the study (27.8%, 15 participants). The groups had similar withdrawal distribution.

The demographic and clinical data and ACT variables are shown in Table 2; the data reflect standard characteristics of the population of children with CF and show no statistically significant differences between groups in the baseline measures.

**Table 2:** Demographic and clinical data and baseline ACT variables.

Parameters	Control Group (n = 15)	Placebo Group (n = 13)	Treated Group (n = 15)
**Age, years** ^**1**^	7.2 ± 4.3 (2–16)	8.1 ± 5.6 (2–17)	7.9 ± 4.7 (2–17)
**Age of diagnosis, months** ^**1**^	6.8 ± 8.4 (0.5–24)	6.8 ± 6.8 (1–24)	6.4 ± 7 (0.5–24)
**Male** ^**2**^	8 (53.3)	9 (69.2)	10 (66.7)
**BMI** ^**3**^	16.6 ± 1.7 (13.7–19.4)	16.3 ± 2.2 (12.1–19.1)	16.9 ± 2.2 (12.8–19.6)
**Bhalla score** ^**1**^	19.4 ± 3.8 (13–25)	18 ± 5 (10–25)	18.9 ± 4.7 (9–24)
**ACT frequency per day** ^**1**^	1.3 ± 0.5 (1–2)	1.4 ± 0.5 (1–2)	1.4 ± 0.5 (1–2)
**Time to take an ACT session, minutes** ^**3**^	26 ± 3.4 (10–60)	22.4 ± 3.7 (10–45)	27.3 ± 3.4 (10–60)

Continuous variables are expressed as the mean ± SD (range). Categorical data are expressed as n (%).

^1^ Median ± SD (range): Kruskal-Wallis test was performed to compare groups.

^2^ N (%): Chi square test was performed to compare groups for the variable 'gender'.

^3^ Median ± SD (range): ANOVA test was performed to compare groups. No statistically significant differences were identified.

Abbreviations: BMI, body mass index; ACT, airway clearance therapy. Bhalla score: a lung damage severity rating system for high-resolution computed tomography in cystic fibrosis. The lower the Bhalla score is, the more severe the condition [22].

Concerning the baseline use of adjuncts to ACT prior to the intervention, the most common adjuncts to ACT were the use of television or other audiovisual devices by 28 (66.7%) participants, while music was used by 10 (23.8%) participants. There were no statistically significant differences in the ACT baseline adjunct measures between the 3 groups. During the intervention, audiovisual devices were frequently used as adjuncts to ACT in the CG (12, 80%), while 12 (80%) and 11 (73.3%) patients in the TG used the music CD frequently after 6 or 12 weeks, respectively. The frequent use of familiar music decreased during the intervention, being used by 10 (76.9%) participants after 6 weeks and 6 (46.2%) participants after 12 weeks. Throughout, participants in the TG and PG used music more than 50% of the time.

**Fig 2:**
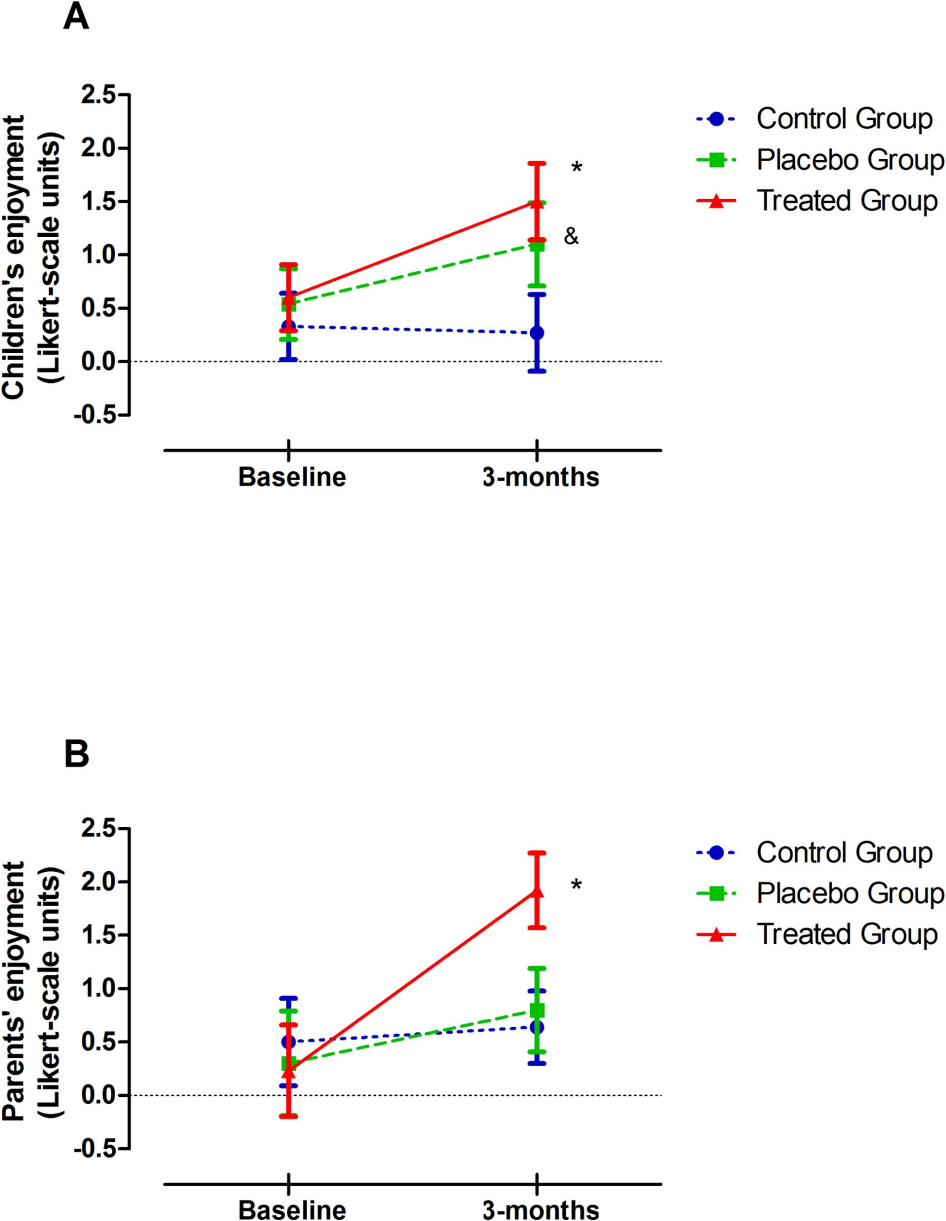
Change in children’s and parents’ enjoyment. (A) Children's and (B) parents' enjoyment. Enjoyment was assessed on 7-point Likert-type scales, where—3 was the least enjoyment, + 3 was the most enjoyment, and 0 was neutral. Values were measured at baseline and in the final interviews. The data are presented as the mean (± SD). Wilcoxon test was performed to compare the differences between the baseline and final measures in each group. Changes in children’s enjoyment: *p = 0.004 (Treated Group); ^&^p = 0.035 (Placebo Group); p = 0.89 (Control Group). Changes in parents’ enjoyment: *p = 0.009 (Treated Group); p = 0.096 (Placebo Group); p = 0.492 (Control Group).

The results on children’s enjoyment are shown in Fig 2A. The differences between the 3 groups did not reach statistical significance (group, p = 0.197). However, the change over time in children's enjoyment between the groups was statistically significant (interaction, p = 0.042), and a statistically significant change in children’s enjoyment before and after the intervention (time points, p = 0.006) was observed. The use of the specifically composed music or the familiar music was associated with a positive change in children’s perceived enjoyment of ACT after 12 weeks compared to the use of no music. Thus, the change in enjoyment from baseline was +0.9 units for the TG (p = 0.004) and +0.56 units (p = 0.035) for the PG compared with no change in the CG (-0.06 units). A similar response was observed for parents’ enjoyment (Fig 2B); the use of the music CD (+1.7 units, p = 0.009) and familiar music (+0.5 units) was associated with a positive change in parents’ perceived enjoyment of ACT after 12 weeks compared with no music (+0.14 units). Again, the differences between the 3 groups did not reach statistical significance (group, p = 0.493). However, the change over time in parents’ enjoyment between the groups was statistically significant (interaction, p = 0.011), and a statistically significant change in parents’ enjoyment before and after the intervention (time points, p = 0.001) was observed.

Moreover, the evaluation of the descriptive words chosen to describe the response to ACT showed that, after use of the music CD, no negative responses were expressed by the children and parents, while prior to the use of the specific music, 6 (40%) children and 4 (30.8%) parents expressed negative perceptions. No changes in negative response were observed in the CG or PG patients or parents. In addition, no adverse effects from the experimental conditions were observed.

**Fig 3:**
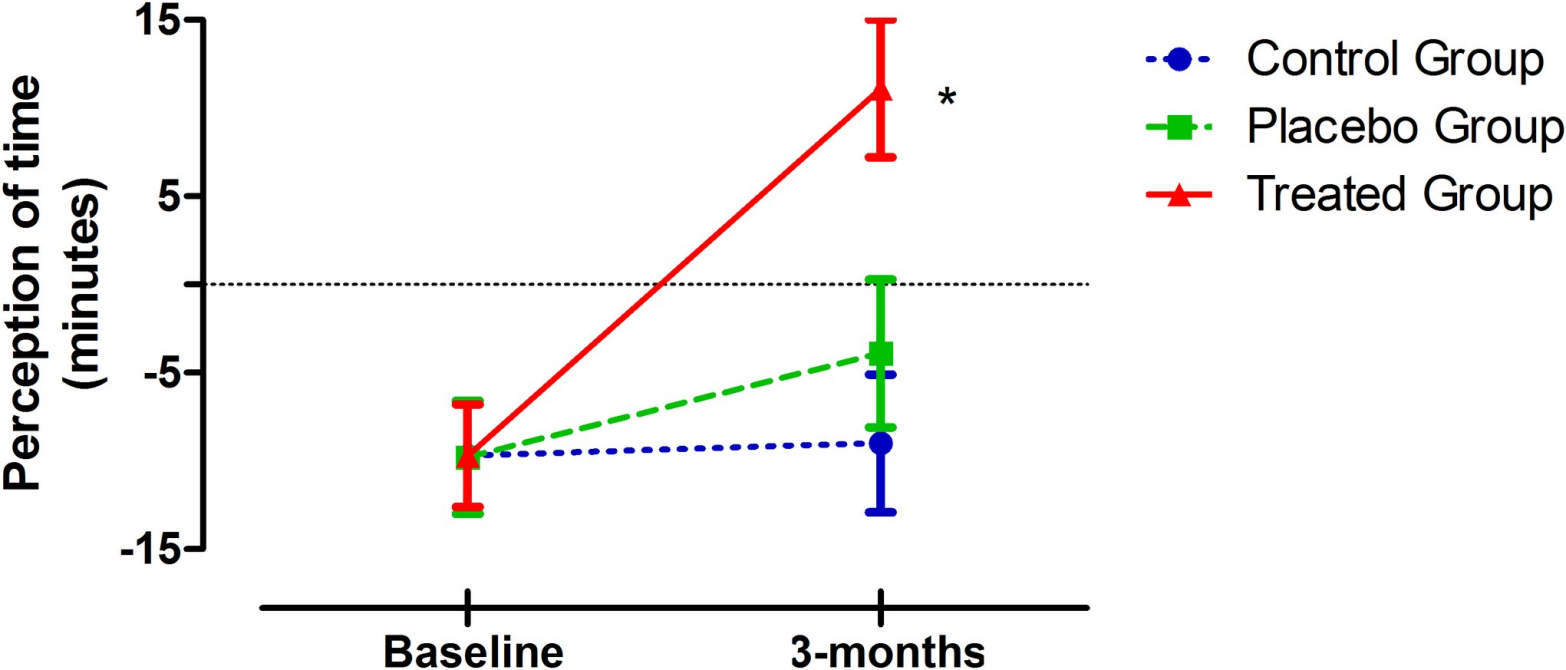
Change in perception of time. To analyze the perception of time, the apparent time value reported on the questionnaire was subtracted from the actual time value given (actual time—apparent time = perception of time). Positive value: perception of ACT shorter. Negative value: perception of ACT longer. Values above 0: participants felt they spent less time taking the ACT routine than the actual time they spent to taking it. Values below 0: participants felt they spent more time taking the ACT routine than the actual time they spent taking it. Differences were calculated at baseline and the final interviews. The data are presented as the mean (± SD). Wilcoxon test was performed to compare the differences between the baseline and final measures in each group. Changes: *p = 0.003 (Treated Group); p = 0.076 (Placebo Group); p = 0.857 (Control Group).

Concerning the analysis of the outcome 'perception of time', based on Grasso and colleagues study [14], the perception of time taken to complete the ACT routine changed positively after the intervention. Thus, the use of music CD as an adjunct to ACT routine not only eliminated the feeling of spending much more time taking the ACT routine, but also led participants to feel the length of the ACT 11.1 min (± 3.9) shorter than the actual time they spent to carry it out. However, PG and CG continued feeling the length of ACT routine longer than the actual time they spent taking it. Thus, PG felt the length of routine 3.9 min (± 4.2) longer than actual time, and no changes were observed in the CG, feeling the length of the routine 9.0 min (± 3.9) longer than actual time (Fig 3). A statistically significant change in the perception of time before and after the intervention (time points, p = 0.001), a statistically significant interaction of time and group (interaction, p = 0.004), and statistically significant differences in the perception of time between the 3 groups (group, p = 0.038) were observed (Fig 3).

The analysis of the use of the specifically composed, played and compiled music as an adjunct to ACT compared to the use of familiar music chosen based on the recommendations given in the study showed a clear positive response in children and parents (2.0±1.4 units, in both cases) to the use of the music CD and a positive response to the use of familiar music (1.0±1.4 units, children; 0.9±1.4 units, parents), with statistically significant differences (p = 0.029, children; p = 0.026, parents). In addition, the music CD was considered useful by 14 (93.3%) participants (p = 0.023), while the familiar music was useful only to 7 (53.8%). All participants except one (93.3%) in the TG reported that they would consider using this music or new recorded music in the future (p = 0.03), but only 5 (38.5%) participants in the PG said they would consider the use of familiar music. Anecdotal reports indicated that the music CD facilitated the relaxation of children and increased enjoyment of ACT (12, 85.7%), helping divert children’s and parents’ attention away from the time spent on ACT. An enhancement of ACT management was also reported. However, only 2 (28.6%) participants in the PG reported these facts. Concerning the time to take an ACT session, the TG tended to spend more time per session than the PG (37.1±18.9 min vs. 27.2±12.1 min, p = 0.09).

The number of respiratory infection exacerbations that required hospitalization in each group and their related cost are shown in Table 3. The average length of hospital stay was 13.66 days. According to the official DRG prices published in Spain, a hospitalization for CF costs a total of €6,704.87. As we can observe, only one child suffered an exacerbation during the intervention in the TG, while 3 and 6 exacerbations were reported in the PG and CG, respectively. Conversely, during the preintervention period, 2 exacerbation episodes were suffered in the TG group, and one each was reported in the PG and CG groups. Based on these data, the use of the music CD as an adjunct in the ACT yielded a potential cost saving of €6,704.87. However, in the CG, the cost increased to €33,524.35, and in the PG, the cost increased to €13,409.74.

**Table 3:** Healthcare resources in children with cystic fibrosis according to the different groups.

Groups	Preintervention		During the intervention		Change	
	Exacerbations (n)	Related cost (€)	Exacerbations (n)	Related cost (€)	Exacerbations (n)	Related cost (€)
**Treated (n = 15)**	2	13,409.74	1	6,704.87	-1	-6,704.87
**Placebo (n = 13)**	1	6,704.87	3	20,114.61	+2	+13,409.74
**Control (n = 15)**	1	6,704.87	6	40,229.22	+5	+33,524.35

Preintervention: 3-month period before the intervention; intervention: 3-month trial period; exacerbation: number of respiratory infection exacerbations that required hospitalization. Hospitalization cost (€) was estimated based on the associated diagnosis-related groups.

## Discussion

The results of this study indicate that the specifically composed, played and compiled instrumental recorded music is an effective adjunct to ACT. Overall, its use demonstrates effectiveness in improving children’s and parents’ enjoyment of ACT, reducing the perception of time taken to complete ACT. Furthermore, the use of the music CD can be considered an efficient option in terms of the avoided costs related to respiratory infection exacerbations.

Although patients and parents consider ACT to be an integral part of CF management, they report serious difficulties in carrying it out [23,24]. Chest physiotherapy requires significant commitments of time and energy, being crucial to establish ACT as a well-structured positive routine [7,23–25]. Providing enjoyable and stimulating strategies can be an effective tool for increasing the participation of the population in health-oriented activities [26]. In rehabilitation programs for chronic obstructive pulmonary disease, patient enjoyment is a factor enabling patients to engage in these programs [27,28]. These enjoyable innovative strategies also include MT interventions that are carried out in the home or community groups [29–31]. Regarding children with CF, the use of the music CD as an adjunct to ACT elicited a positive affective response in patients and parents, in line with the findings reported in infants for the use of specific music during ACT [14] or the use of motivational music while walking by children with CF [15]. Concerning the utilization of familiar music based on recommendations, enjoyment was also increased, and similar results were also found in infants [14]. However, the characteristics of the music compiled on the CD duplicated the positive effect of familiar music, with the music CD being the best option. In addition, no negative descriptive words about ACT were chosen by children or parents after using the music CD. It is evident that certain musical elements that were taken into account at the time of the composition and playing of this therapeutic music had an important influence on the positive attitudes that were observed. Therefore, the results of the current work together with the previous infant study [14] indicate that music could be used to assist in the establishment of ACT as a positive routine and that the use of particular music that is composed, played and compiled specifically for children with CF enhances this positive effect.

The music CD used in the current study incorporated elements to encourage positive responses, ACT rhythmical interaction and distraction. Thus, the music included fluid rhythms [32], varied and bright timbres [32–34], intensity changes that were not excessively marked [32,33], variable sound frequencies [32] and mainly major tonalities [35]. Moreover, the music CD incorporated slow rhythms [15,36], intervals and consonant chords [34,37] and sounds without excessively rapid vibrations [33] to elicit relaxation.

Music therapy studies in the pediatric context have demonstrated that the selection of appropriate music and its elements can positively alter children’s experiences of medical interventions through distraction and/or relaxation [38–45]. Therefore, it is known that induced relaxation during appropriate music listening is one of the novel MT strategies implemented in the management of pulmonary diseases [46]. Relaxation is essential in the course of drug inhalation to achieve an optimal drug concentration in the lungs. In this study, the music CD could facilitate distraction and relaxation during ACT, improving the management of the own ACT. Thus, reports about the use of the music CD suggest that the music itself provided distraction, relaxation and rhythmic support for the management of nebulizer inhalation treatment and airway clearance techniques. In addition, because the music CD was structured in 3 sections with similar lengths to each ACT section, children and/or parents were not required to time the sections of ACT, and the music reportedly distracted them from the time spent on the routine.

Regarding the clear change in perception of time observed after using the music CD, it is evident that certain music elements have an important influence on this variable. Certainly, the use of the music CD decreased the perception of ACT as a long activity, while this effect was not found in the PG or CG. Although no significant change in perception of time was observed in the infant study [14], the anecdotal responses indicated that the time spent doing ACT with specific recorded music seemed to pass more quickly than during their usual routine.

Music can be effective as a distracting stimulus when used in pulmonary rehabilitation training for chronic obstructive pulmonary disease. Among the benefits of music is an increase in the total time spent on therapy sessions [47,48]. In this sense, the music CD also had an effect on the time to take an ACT session in our study. During the intervention, the use of the CD increased the time spent per session, making the time spent close to the required session time [5,49]. Furthermore, 2 patients in the TG increased the ACT frequency per day from one to 2 times.

Regarding the evaluation of its efficiency, although our MT strategy was longer than many MT interventions that have been carried out for pulmonary diseases (2 to 10 week time span) [10,12,13,30,50], the measure of rate of lung function decline (as FEV1% predicted/year), may not be appropriate [51,52]. Therefore, we had to use a clinical outcome that could be measured during this follow-up, i.e., respiratory infection exacerbations. Thus, the efficiency of the intervention was evaluated in terms of avoided healthcare resources to treat respiratory infection exacerbations that required hospitalization, and the use of the music CD as an adjunct to ACT could represent a potential cost saving.

Other factors that could affect the results were not analyzed, for instance, those focused on enhancing the management of ACT [53,54]. The most appropriate ACT and its management for each child are assessed in our Pediatric Pulmonology Unit periodically. However, some home environment factors, such as physical space, are hardly controllable.

The strength of the current intervention is that the study design included an independent PG. However, participants in the CG in the infant study [14] experienced 2 conditions: a control and a placebo.

This study has limitations. First, our results were based on data from a single center. However, this study included half of the children with CF in our region with more than 8 million inhabitants. Second, due to the nature of intervention, it was only possible to blind outcome assessors, and there may be potential bias. Third, 11 participants were withdrawn from the study because they could not be contacted by telephone to be interviewed (n = 3), rejected their consent as a result of family moved (n = 2) or used the music CD (n = 2) or familiar music (n = 4) as an adjunct the ACT less than 50% of the time, but the number withdrawn was still lower than that initially estimated for this study (15 participants). Moreover, the withdrawn participants were similarly distributed among the 3 groups, with the final sample size equal to or above the sample size necessary in each group to enable valid answers to the research question (13 participants).

Further long-term studies are needed to analyze whether this positive experience of ACT has any sustained effects, patients’ perspectives, as well as the efficiency of the intervention in terms of lung function.

In summary, we present the first MT intervention for children with CF between the ages of 2 and 17, demonstrating that the specifically composed, played and compiled instrumental recorded music is an effective adjunct to ACT without adverse reactions and is an efficient option in terms of avoided costs. The music CD improves children’s and parents’ enjoyment of ACT, reducing the perception of time taken to complete ACT. Moreover, this music can provide ACT rhythmic support, distraction and relaxation improving the management of the own ACT. Therefore, this MT intervention we can expect better clinical outcomes in CF patients without changes in treatment regimens or programmed clinic visits in CF units.

## Supporting information

S1 File CONSORT checklist. (PDF)

S2 File Trial study protocol—original. (PDF)

S3 File Trial study protocol—english version. (PDF)

S4 File Music characteristics. (DOCX)

S5 File Music scores. (PDF)

S6 File Questionnaires. **Music CD.** Because the recorded music was compiled in a physical format, i.e., an audio compact disc, the songs used in this study are available from the corresponding authors.
(PDF)
