# Supplementary material for: Effects of music therapy as an adjunct to chest physiotherapy in children with cystic fibrosis: A randomized controlled trial
Source: PLoS One. 2020 Oct 30;15(10):e0241334. doi: 10.1371/journal.pone.0241334 (PMC7598495; doi:10.1371/journal.pone.0241334)
Supplement: S2 File — (PDF) [file pone.0241334.s002.pdf]

PROTOCOLO INVESTIGACIÓN

Título del proyecto:
Efectos de la musicoterapia como complemento de la fisioterapia respiratoria en pacientes con fibrosis quística

Investigador principal:
Dra. Elisa Martín Montañez. Profesora Departamento de Farmacología y Pediatría de la Facultad de Medicina de la Universidad de Málaga.

Investigadores colaboradores:
Dr. Javier Pérez Frías. FEA Neumología Infantil. Hospital Regional Universitario de Málaga, Hospital Materno-Infantil. Catedrático de Pediatría de la Universidad de Málaga en el Dpto. de Farmacología y Pediatría de la Facultad de Medicina de la Universidad de Málaga.

Dra. Laura A. Fuentes Gálvez. Fisioterapeuta especialista en fisioterapia respiratoria infantil. Colaboradora Dpto. de Farmacología y Pediatría de la Facultad de Medicina de la Universidad de Málaga.

Don Alberto Montero Ruiz. Profesor de música, titulado superior especialidad de percusión. Colaborador Dpto. de Farmacología y Pediatría de la Facultad de Medicina de la Universidad de Málaga.

Objetivo principal: Desarrollar una estrategia basada en la musicoterapia como complemento a la fisioterapia respiratoria diaria en niños con fibrosis quística y evaluar sus efectos.

Diseño del proyecto de investigación: Estudio experimental- ensayo clínico controlado aleatorizado. Ámbito: unicentrico. Hospital Materno-Infantil. Neumología Infantil.

Enfermedad o trastorno en estudio: Fibrosis quística**.**

Población en estudio y número total de sujetos: Niños de 2-17 años que acuden a la consulta de Neumología Infantil de la Unidad de Fibrosis Quística del Hospital Materno-Infantil de Málaga. El número necesario de pacientes que finalicen el estudio se ha definido en 13 en el grupo de intervención e igual número en los grupos control y control con música comercial siendo necesaria la inclusión de 39 pacientes en total.

Calendario: Intervención de 2 años de duración.

ANTECEDENTES Y ESTADO ACTUAL DEL TEMA DE ESTUDIO

En los pacientes con fibrosis quística (FQ), enfermedad rara, crónica, multisistémica que afecta ampliamente la calidad de vida del paciente, el deterioro progresivo de la función pulmonar y sus manifestaciones clínicas se presentan en el 95% de los pacientes (CFF, 2014). Estas manifestaciones clínicas se relacionan principalmente con su morbi-mortalidad (Alexander et al, 2014; CFF, 2014). En definitiva, la merma de la capacidad pulmonar y sus consecuencias contribuyen a una degeneración del tejido pulmonar, lo que a la larga hace necesario el trasplante pulmonar como única solución, incluso a edades tempranas (Prados et al, 2000; CFF, 2014). Se hace imprescindible por tanto instaurar un tratamiento adecuado que evite la progresión de la enfermedad pulmonar. Dentro de este tratamiento, la fisioterapia respiratoria (FR) incluida en el abordaje terapéutico habitual tiene un papel esencial en el mantenimiento de la función pulmonar ya que los trastornos respiratorios del paciente con FQ se caracterizan por un exceso de secreciones respiratorias y un deterioro de la aclaración de las mismas y el principal objetivo de la FR en FQ es mejorar el aclaramiento mucociliar y el drenaje de las secreciones (McIlwaine et al, 2014).

Las técnicas convencionales de FR tratan la obstrucción bronquial aplicando métodos tales como la vibración, la percusión o el drenaje autógeno (Pisi y Chetta, 2009). Para la aclaración mucociliar se utilizan respiraciones profundas y, por medio de la tos, las secreciones son arrastradas y desplazadas hacia el exterior (Main et al, 2011). Estas técnicas de FR (CFF, 2014) se realizan normalmente 2 veces al día durante una media de 45 minutos por sesión. Por tanto la FR requiere diariamente un compromiso significativo de tiempo y energía que la convierte en una rutina tediosa tanto para el paciente como para sus familiares complicando su adherencia (Modi & Quittner, 2006) y reduciendo sus beneficios (Sabaté, 2003; Goodfellow et al, 2015).

La música tiene gran valor motivacional en las enfermedades llevando a una mejora psicosocial (Le Roux et al, 2007). Las intervenciones de musicoterapia suelen integrarse en estrategias para la mejora física y psicoemocional en enfermedades pulmonares, caso de la enfermedad pulmonar obstructiva crónica (Bausewein et al, 2013; Panigrahi et al, 2014) donde a través de la música, el paciente alcanza estados emocionales positivos reduciendo la ansiedad y la depresión (Canga et al, 2015). En este sentido, hay pocas intervenciones de musicoterapia en FQ (Goldbeck et al, 2014; Irons et al, 2014). Recientemente, se ha descrito que una música motivadora cuidadosamente seleccionada puede llevar a una respuesta afectiva positiva durante la realización de 6 minutos de ejercicio físico en pacientes con FQ, haciendo este ejercicio agradable (Calik-Kutukcu et al, 2016). Sólo existe un estudio australiano realizado en bebés y niños menores de 2 años donde, una música adecuada creada específicamente para la FR de estos niños y

sus familiares, ayuda a instaurar la FR como rutina al convertirla en una experiencia positiva (Grasso et al, 2000) mejorando, en definitiva, su calidad de vida.

HIPOTESIS DE PARTIDA

La adherencia al tratamiento en general de las enfermedades crónicas es muy baja (Sabaté, 2003). En este sentido la adherencia al tratamiento de la fibrosis quística lo es también, alcanzándose una adherencia a la fisioterapia respiratoria inferior al 50% (Goodfellow et al, 2015). No existe una alternativa terapéutica a la fisioterapia respiratoria para mejorar el aclaramiento mucociliar en estos pacientes, en todo caso, se puede combinar y es deseable, con la realización de ejercicio físico (Kriemler et al, 2016). Mejorar la motivación hacia la fisioterapia respiratoria es la mejor manera de optimar la adherencia a un tratamiento (Smith et al, 2010) y hasta el momento la utilización de dispositivos multimedia, TV, radio, música comercial, lectura de un cuento etc, no mejoran la adherencia en estos pacientes. Una música cuidadosamente seleccionada para el desarrollo de la fisioterapia respiratoria (Grasso et al, 2000) o el ejercicio físico (Calik-Kutukcu et al, 2016) en estos pacientes puede llevar a una respuesta afectiva positiva hacia la actividad que se podría traducir en una mejora en la adherencia (Smith et al, 2010) y por tanto en el aclaramiento mucociliar que podría frenar/mejorar el deterioro de la función pulmonar además de mejorar la calidad de vida del paciente y su entorno.

Hipótesis de partida: Se esperan diferencias significativas en la actitud y percepción hacia la fisioterapia respiratoria diaria, en el grado de adherencia, sintomatología pulmonar y calidad de vida entre aquellos pacientes que reciban el tratamiento combinado de fisioterapia respiratoria convencional complementada con la música terapéutica (grupo de intervención) y aquellos en los que se realice fisioterapia respiratoria convencional con música comercial (grupo control con música) o sin música (grupo control sin música).

OBJETIVOS

Objetivo primario:

Desarrollar una estrategia basada en la musicoterapia como complemento a la fisioterapia respiratoria diaria en niños con fibrosis quística y evaluar sus efectos. Para ello en concreto se plantea la composición, interpretación y edición de música terapéutica instrumental específicamente para pacientes pediátricos con fibrosis quística que complemente la fisioterapia respiratoria convencional que realizan.

Objetivos secundarios:

• Valorar los efectos de la musicoterapia como complemento de la fisioterapia respiratoria sobre la actitud, percepción y adherencia del paciente y familiares hacia la fisioterapia respiratoria.

• Valorar los efectos de la musicoterapia como complemento de la fisioterapia respiratoria sobre la sintomatología pulmonar del paciente y su calidad de vida.

METODOLOGÍA, PLAN DE TRABAJO Y CRONOGRAMA

METODOLOGÍA

- Diseño:

Estudio prospectivo de intervención sobre pacientes pediátricos con FQ. Estudio experimental- ensayo clínico controlado aleatorizado.

- Ámbito de estudio:
Pacientes adscritos al Hospital Materno Infantil de Málaga.

- Sujetos de estudio:
Pacientes que acudan a la consulta de Neumología Infantil de la Unidad de Fibrosis Quística del Hospital Materno-Infantil de Málaga y que cumplan los siguientes criterios de inclusión / exclusión:

- Criterios de inclusión:

- Diagnóstico de fibrosis quística según los criterios diagnósticos internacionalmente aceptados (Aldana et al, 2011).
- Pacientes con edades comprendidas entre 2-17 años.
- Seguir controles periódicos en las consultas de la Unidad de Fibrosis Quística.
- Entender el propósito del estudio.
- Haber otorgado el consentimiento informado.

- Criterios de exclusión:

- Pacientes que no realicen fisioterapia respiratoria.
- Pacientes con problemas auditivos que le impidan beneficiarse de la intervención de musicoterapia.
- Pacientes con riesgo radiológico o clínico de sufrir neumotórax o neumomediastino.
- Pacientes con barotrauma en el mes previo al ingreso en el estudio.
- Paciente con antecedentes de hemoptisis masiva o amenazante.
- Pacientes trasplantados o en lista de espera para trasplante.

Los pacientes/tutor legal deberán haber entendido el objetivo del estudio y expresar su conformidad mediante la firma del Consentimiento Informado previo a la inclusión en el estudio (modelo adjuntado).

- Tamaño muestral:

Para calcular el tamaño de la muestra, se ha asumido la percepción del tiempo necesario para completar la rutina como variable principal. De acuerdo con Grasso et al, (2000), donde los valores del grupo control en esta variable fueron 0.2 ± 10.2 (media ± SD), considerando estos valores como línea de base, una potencia del 80%, un nivel de confianza del 95% y una variabilidad similar al final del estudio, con un tamaño de muestra de 13 sujetos se consideraría estadísticamente significativa una diferencia de 8 minutos. En este estudio son necesarios 39 participantes en total.

- Selección de la muestra:

Tras haber firmado el consentimiento informado se realizará una asignación aleatoria de los pacientes a cada uno de los grupos, utilizando para ello el listado aleatorizado generado mediante el programa estadístico Epidat.

Los grupos definidos son los siguientes:

- *Grupo de intervención o GT* (pacientes que complementan la fisioterapia respiratoria con la música desarrollada específicamente para este fin).
- *Grupo control sin música o GC* (grupo que desarrolla la fisioterapia respiratoria sin música).
- *Grupo control con música o GP* (grupo que acompaña la fisioterapia respiratoria con música comercial elegida por el propio paciente).

En todos los grupos los pacientes continuarán con su régimen habitual de tratamiento.

- Recogida de datos:

De cada paciente se recogerán los datos indicados en el cuaderno de recogida de datos (Anexo) en el que se incluyen variables demográficas y clínicas. En concreto: edad, sexo del paciente, exacerbaciones respiratorias que requieren hospitalización.

Estos pacientes son controlados en la Unidad de Fibrosis Quística del Hospital Materno-Infantil, disponiéndose de una historia clínica detallada donde se incluyen de forma sistemática las variables clínicas y se actualiza el tratamiento que siguen para su enfermedad, por tanto la recogida de datos en este estudio no supone información adicional a la incluida de forma rutinaria en su historia clínica. Esta información la cumplimenta el pediatra investigador a partir de las visitas programadas a la consulta tanto previas al estudio como durante el desarrollo y seguimiento de la intervención, lo que no supone ni un incremento en el número de visitas a la unidad para estos pacientes ni una alteración en su tratamiento y seguimiento habitual.

Además, previo al comienzo de la intervención, y siempre tras ser incluido en el estudio una vez firmado el consentimiento informado, para conocer la percepción hacia la rutina

de FR del paciente y del familiar o familiares que participan en su desarrollo, se realizará una entrevista sobre su experiencia en general con la FR. Durante la intervención se volverá a entrevistar a los pacientes para conocer la evolución de la percepción a lo largo del estudio. Los cuestionarios utilizados antes y durante la intervención, son una adaptación a nuestro entorno y características de nuestros pacientes de cuestionarios validados y diseñados específicamente para conocer características del desarrollo de la FR y la medición de la adherencia, actitud y percepción del tiempo en estos pacientes durante el desarrollo de la rutina fisioterapéutica diaria y la implementación de música terapéutica (Grasso et al, 2000). También se preguntará por la percepción de la sintomatología pulmonar y el impacto sobre la calidad de vida según la adaptación a las características de nuestros pacientes de la escala visual analógica (EVA) para evaluar la magnitud de la disnea utilizada en pacientes con enfermedad pulmonar entre ellos pacientes con FQ (Bausewein et al, 2007) y el cuestionario revisado de calidad de vida para FQ (Quittner et al, 2000).

- Variables del estudio:

- *Demográficas*:

  - Edad (Variable cuantitativa) (Unidad: años)

  - Sexo (Variable cualitativa dicotómica) (Hombre / Mujer)

- *Clínicas*:

  - Número exacerbaciones respiratorias que requieren hospitalización (Variable cuantitativa)

  - Días de hospitalización por cada exacerbación (Variable cuantitativa)

- *Características del desarrollo de la fisioterapia respiratoria*:

  - Complemento de la rutina de fisioterapia con algo (Variable cualitativa dicotómica) (SI /NO)

  - Uso de complementos durante la fisioterapia respiratoria (juguetes, cuentos, música, radio, TV…) (Variable cualitativa)

- *Adherencia fisioterapia respiratoria*:

  - Frecuencia de realización fisioterapia (Variable cuantitativa)

  - Número de veces al día (Variable cuantitativa)

  - Interrupciones en su desarrollo (Variable cualitativa dicotómica) (SI /NO)

  - Duración de cada sesión (Variable cuantitativa)

- *Actitud hacia la fisioterapia respiratoria:*

  - Definición de la fisioterapia respiratoria como desagradable - neutra - agradable según escala de Likert (-3 a +3)

  - Sentimientos hacia la rutina de fisioterapia respiratoria según elección de 3 palabras (Variable cualitativa)

- *Percepción del tiempo para la realización de la fisioterapia respiratoria*:
  - Percepción de ser una rutina larga (Variable cualitativa dicotómica) (SI /NO)
  - Tiempo que le parece necesitar para realizarla (Variable cuantitativa)

- *Sobre la música*:
  - Frecuencia de utilización de la música (Variable cuantitativa)
  - Respuesta a la utilización de la música durante la rutina de fisioterapia respiratoria como desagradable, neutra, agradable según escala de Likert (-3 a +3)
  - Utilidad de la música en la rutina de fisioterapia (Variable cualitativa dicotómica) (SI /NO)
  - Manera en que ha sido útil la música (Variable cualitativa)
  - Continuación de la utilización de esta música como complemento a la fisioterapia respiratoria (Variable cualitativa dicotómica) (SI /NO)

- *Percepción sintomatología pulmonar e impacto sobre la calidad de vida:*
  - Sensación pulmonar definida como dificultad para respirar según escala visual analógica (EVA) para evaluar la magnitud de la disnea donde 1 significa no falta de aire y 7 máxima falta de aire
  - Frecuencia de sintomatología según cuestionario revisado de calidad de vida para FQ durante las 2 semanas anteriores sobre aparición de tos durante el día y la noche, expectoración y tipo, dificultad para respirar, pitos al respirar, congestión.

- Desarrollo de la música terapéutica instrumental:

En este trabajo, se pretende desarrollar una música terapéutica instrumental dirigida específicamente a nuestros pacientes pediátricos y sus familiares adaptada a las características de la FR que realizan según las directrices de la Unidad de Fibrosis Quística a la que pertenecen.

El tratamiento de FR en esta unidad está dividido en 3 partes con una duración total de unos 40 minutos por sesión: En la primera parte, el paciente debe estar relajado mientras se le aplica el tratamiento nebulizador durante unos 10-15 minutos. Seguidamente, se procede al trabajo de fisioterapia en sí y limpieza bronquial durante unos 20-30 minutos con la aplicación de percusiones y vibraciones que favorezcan la subida del moco y su posterior expulsión. Y para terminar la sesión, se plantea una nueva fase de relajación de unos 5 minutos donde se suelen de nuevo administrar tratamiento nebulizador.

La música que se pretende desarrollar, se divide en 3 secciones según las 3 partes del tratamiento con una duración similar a cada una de las partes:

- Sección A: tratamiento nebulizador
- Sección B: trabajo de fisioterapia - limpieza bronquial
- Sección C: relajación-nebulización

El músico titulado superior del equipo investigador compondrá y realizará las partituras de diferentes piezas musicales en cada sección según las pautas indicadas por los profesionales clínicos del equipo. Se estima la composición de unas 10 piezas repartidas entre las tres secciones.

Una vez compuestas, el músico titulado superior en la especialidad de percusión, interpretará y grabará las obras con instrumentos de percusión de afinación determinada (glockenspiel, marimba, vibráfono y xilófono) e indeterminada (batería, pailas, congas, pequeña percusión).

Para la grabación de audio el músico titulado superior utilizará un estudio portátil (Tascam) y la microfonía específica dependiendo del tipo de instrumento musical. Posteriormente, el audio será editado con el programa informático de edición Cakewalk Sonar Platinum. Se entregará un CD de audio a los pacientes del grupo de intervención.

- Intervención:

Una vez reclutados los pacientes y realizada la *entrevista inicial* en función del grupo de pertenencia (grupos GT, GC o GP), se pedirá a los pacientes que durante 6 semanas (Grasso et al, 2000; Canga et al, 2015) realicen su rutina de FR habitual:

- *Pacientes Grupo de intervención o GT:* complementada con la música desarrollada específicamente para este fin. El CD de música se proporcionará tras la entrevista.
- *Pacientes Grupo control sin música o GC*: Continuarán desarrollando su fisioterapia respiratoria de la manera habitual y sin el CD de música.
- *Grupo control con música o GP:* complementada con música comercial elegida por el propio paciente.

Como se ha comentado el tratamiento de FR en esta unidad está dividido en 3 partes con una duración total de unos 40 minutos por sesión: En la primera parte, el paciente debe estar relajado mientras se le aplica el tratamiento nebulizador durante unos 10-15 minutos. Seguidamente, se procede al trabajo de fisioterapia en sí y limpieza bronquial durante unos 20-30 minutos con la aplicación de percusiones y vibraciones que favorezcan la subida del moco y su posterior expulsión. Y para terminar la sesión, se plantea una nueva fase de relajación de unos 5 minutos donde se suelen de nuevo administrar tratamiento nebulizador.

En todos los grupos los pacientes continuarán con su régimen habitual de tratamiento. En ningún caso se plantea la modificación del seguimiento o de los tratamientos prescritos por los facultativos responsables del paciente según el protocolo vigente del hospital.

Tras las 6 semanas, se realizará una *segunda entrevista* y se pedirá a los pacientes que continúen desarrollando la FR de la manera indicada según el grupo al que pertenecen

durante otras 6 semanas. A los 3 meses (Grasso et al, 2000) desde el inicio de la intervención, se volverá a entrevistar a los pacientes. Tras estos 3 meses, se le ofrecerá tanto al grupo GC como al grupo GP el CD de música para que si lo desean complementen su rutina de FTR con la música desarrollada para esta intervención.

- Análisis estadístico de datos:
Se llevará a cabo con el software del paquete estadístico SPSS licenciado para la Universidad de Málaga.

Inicialmente se realizará un análisis descriptivo de las variables del estudio, los valores de las variables continuas se resumirán en una tabla donde se mostrarán sus correspondientes medias y desviación estándar. Las variables categóricas se presentarán en frecuencias absolutas y frecuencias relativas.

Para analizar que las diferencias observadas en las variables de interés son estadísticamente significativas, en el caso de variables cualitativas se aplicará el test de la Chi-cuadrado o la prueba exacta de Fisher en el caso de que el porcentaje de valores esperados menores de 5 supere el 20%. Para analizar las diferencias observadas en las variables cuantitativas, se aplicará ANOVA y, en el caso de que no se verificara la condición de normalidad, el test no paramétrico de Kruskal Wallis.

Para contrastar las diferencias en la evolución de variables entre los diferentes grupos se aplicará el test multivariante ANOVA de medidas repetidas (MANOVA) con un factor entre sujetos (grupo) y un factor intra sujetos (diferentes momentos de la intervención). Dependiendo del cumplimiento de la condición de esfericidad, se utilizará la corrección de Greenhouse-Geisser. Para contrastar las diferencias entre momento inicial y final en cada grupo se aplicará el test de Wilcoxon.

Para comparar la respuesta al uso de música después de la intervención en TG y PG, se utilizará la prueba U de Mann-Whitney.

Se requerirá una significación del 5% ($p < 0,05$) para considerar una diferencia como estadísticamente significativa.

- Limitaciones:
Sesgo de selección, las personas que declinan participar en el estudio pueden introducir un sesgo de selección, esto se puede controlar en la fase de análisis de datos, analizando si existen diferencias entre los que participan y los que no.

PLAN DE TRABAJO

El grupo investigador se reunirá para definir la música instrumental que se va a componer como complemento a cada una de las partes de la fisioterapia respiratoria habitual del paciente con FQ. Tras la reunión, el músico titulado superior miembro del equipo investigador compondrá y realizará las partituras de las obras musicales,

interpretará y grabará dichas obras con la utilización de diversos instrumentos de percusión de afinación determinada e indeterminada.

El grupo investigador supervisará y seleccionará la música que se vaya componiendo para perfilar la adecuación de las obras a cada momento de la fisioterapia respiratoria. Una vez seleccionada el músico titulado superior editará la música terapéutica instrumental según las pautas de la consulta de neumología y la fisioterapeuta especialista en fisioterapia respiratoria infantil.

Se preparará para su difusión la música elegida en un CD. A partir de este momento, se procederá al reclutamiento de pacientes por parte de la consulta de Neumología Infantil de la Unidad de Fibrosis Quística y al desarrollo de la intervención:

A los pacientes del grupo de intervención se les pedirá que complementen cada sesión de FR diaria escuchando la música que se le entrega en el CD durante 6 semanas. En el caso de los pacientes del grupo control con música se les pedirá que complementen su FR diaria con la música comercial que les guste durante 6 semanas y a los del grupo control sin música se les pedirá que continúen con el desarrollo de su FR diaria de forma habitual.

Tras la entrevista inicial, se realizará una entrevista al paciente a las 6 semanas y se le pedirá que continúe desarrollando la FR como en estas 6 últimas semanas. A los 3 meses (es decir 6 semanas más tarde) se volverá a entrevistar a los pacientes.

Al finalizar el estudio, se ofrecerá tanto al grupo GC como al grupo GP el CD de música para que si lo desean complementen su rutina de FR con la música desarrollada para esta intervención.

En ningún caso se modifica el tratamiento de los pacientes y las visitas de seguimiento clínico son las indicadas por la Unidad de Fibrosis Quística. En ningún caso se verán alterados por la intervención ni el tratamiento habitual de los pacientes ni su seguimiento.

La recogida de datos demográficos y clínicos a cargo del facultativo de la consulta de Neumología Infantil de la Unidad de Fibrosis Quística colaborador en el estudio, se realizará en el cuaderno de recogida de datos diseñado a tal efecto (Anexo).

A partir de los datos obtenidos en el estudio, se procederá a su análisis de forma sistemática, hasta realizar el análisis final y la preparación de comunicaciones a congresos y publicaciones en revistas especializadas.

CRONOGRAMA

Tareas	AÑO 1												AÑO 2											
**Meses**	1	2	3	4	5	6	7	8	9	10	11	12	1	2	3	4	5	6	7	8	9	10	11	12
Reunión de inicio y definición de la música a desarrollar																								
Composición /realización de partituras																								
Interpretación /grabación obras musicales																								
Supervisión y selección de las obras																								
Edición de las obras seleccionadas																								
Análisis anual y final																								
Preparación CD música																								
Reclutamiento																								
Recogida datos demográficos/clínicos																								
Entrevista iniciales y de seguimiento																								
Análisis estadístico																								

CONSIDERACIONES ÉTICAS

Se recuerda en este apartado que en este estudio no se realiza intervención con ningún medicamento o producto sanitario en investigación. Se trata de comparar la técnica de fisioterapia respiratoria convencional incluida en el abordaje terapéutico habitual en los pacientes con fibrosis quística frente a la utilización de música instrumental específicamente compuesta, interpretada y grabada-editada como complemento a esta misma fisioterapia respiratoria incluida en el abordaje terapéutico habitual de los pacientes. Este estudio no reporta ninguna alteración ni en su tratamiento ni en su seguimiento habitual y podría convertir esta rutina de fisioterapia en una rutina positiva. Esta actitud mejoraría su adherencia, lo que supondría una mejora del aclaramiento mucociliar y los beneficios sobre la función pulmonar y calidad de vida.

El perfil de seguridad de las situaciones propuestas: grupo de intervención (fisioterapia respiratoria complementada con la música terapéutica), grupo control sin música (fisioterapia respiratoria sin música) y grupo control con música (fisioterapia respiratoria complementada con música comercial elegida por el paciente) es favorable y el balance beneficio/riesgo óptimo. Tras finalizar la intervención, se le ofrecerá tanto al grupo GC como al grupo GP el CD de música para que si lo desean complementen su rutina de FR con la música desarrollada para esta intervención.

Por otra parte esta intervención de musicoterapia propuesta no conlleva riesgos para los pacientes con FQ sometidos a fisioterapia respiratoria, pues simplemente consiste en complementar su propia FR rutinaria con música.

En todo momento se respetan los principios fundamentales establecidos en las declaraciones, protocolos y convenios nacionales e internacionales sobre ética de la investigación, así como los requisitos establecidos en la legislación nacional y autonómica en el ámbito de la investigación, desarrollo e innovación biomédica, la protección de datos de carácter personal y la bioética.

El proyecto sigue las guías de Buenas Prácticas Clínicas y las recomendaciones éticas contenidas en la Declaración de Helsinki de la Asociación Médica Mundial - Principios éticos para las investigaciones médicas en seres humanos (64ª Asamblea General, Fortaleza, Brasil, octubre 2013). Se respeta lo establecido por la Ley 14/2007, de 3 de Julio de Investigación Biomédica.

El estudio garantiza la confidencialidad de los datos según la Ley Orgánica de Protección de datos de Carácter Personal (Ley Orgánica 15/1999) y a la Ley 41/2002, ley básica reguladora de la autonomía del paciente y de derechos y obligaciones en materia de información y documentación clínica. Los datos que puedan identificar a los pacientes, se mantendrán de forma separada, y estarán identificados por un código. La base de datos del estudio se mantendrá anonimizada a través de este sistema de codificación, por lo que no será posible identificar a los pacientes. Toda la información será utilizada únicamente para los fines reseñados.

Todos los investigadores implicados en el proyecto se comprometen a cumplir las normas necesarias para preservar la confidencialidad de la información facilitada por los participantes.

Este proyecto se presenta al CEI Provincial de Málaga para su aprobación.

Previo a su inclusión en el estudio, los participantes deben conocer y entender el proyecto y consentir su participación, que es voluntaria. Se adjuntan los documentos de Consentimiento Informado y Hoja de información. Como se ha comentado, la participación no supone un cambio respecto al tratamiento de FR convencional en sí, ya que se continuaría aplicando la misma fisioterapia respiratoria incluida en el abordaje terapéutico habitual, pero en este caso para el grupo de intervención complementada con música terapéutica específica y en el grupo control con música, con música comercial. Esto no supone un cambio en su atención médica respecto a la que recibiría de no participar.

APLICABILIDAD

La estrategia propuesta en este proyecto tiene una aplicación directa en la práctica asistencial ya que se prevé una mejora en la actitud y percepción hacia la fisioterapia respiratoria diaria, en el grado de adherencia, sintomatología pulmonar y calidad de vida

entre aquellos participantes que reciban el tratamiento combinado de fisioterapia respiratoria convencional complementada con la música terapéutica sin modificar sus tratamientos ni su seguimiento. Esto llevará a una disminución de la carga de la enfermedad, lo que puede traducirse en mejoras en la práctica del Sistema Sanitario.

BIBLIOGRAFÍA

• Aldana JM, León MT, Salamanca C, Olveira C, Olveira G, Pérez-Frías J, Pérez E, Sierra C, Dapena FJ, Quintana E. Guía asistencial de fibrosis quística. Servicio Andaluz de Salud. Consejería de Salud. Junta de Andalucía. 2011.

• Alexander S, Alshafi K, Anderson AK, Balfour-Lynn I, Bentley S, Buchdahl R, et al. Clinical guidelines for the care of children with cystic fibrosis. Royal Brompton Hospital Paediatric Cystic Fibrosis Team. 2014; 6th edition. Disponible en: www.rbht.nhs.uk/childrencf

• Bausewein C, Farquhar M, Booth S, Gysels M, Higginson IJ. Measurement of breathlessness in advanced disease: a systematic review. Respir Med. 2007. 101:399-410.

• Bausewein C, Booth S, Gysels M, Higginson IJ. Non-pharmacological interventions for breathlessness in advanced stages of malignant and nonmalignant diseases. Cochrane Syst. Rev. 11 (2013) CD005623.

• Calik-Kutukcu E, Saglam M, Vardar-Yagli N, Cakmak A, Inal-Ince D, Bozdemir-Ozel C, Sonbahar-Ulu H, Arikan H, Yalcin E, Karakaya J. Listening to motivational music while walking elicits more positive affective response in patients with cystic fibrosis. Complement Ther Clin Pract. 2016;23:52-8.

• Canga B, Azoulay R, Raskin J, Loewy J. AIR: Advances in Respiration e Music therapy in the treatment of chronic pulmonary disease. Respiratory Medicine 109 (2015) 1532e1539.

• CFF, Cystic Fibrosis Foundation Patient registry 2014 annual data report. Cystic Fibrosis Foundation.

• Goldbeck L, Fidika A, Herle M, Quittner AL. Psychological interventions for individuals with cystic fibrosis and their families. Cochrane Database Syst Rev. 2014. (6):CD003148.

• Goodfellow NA, Hawwa AF, Reid AJ, Horne R, Shields MD, McElnay JC. Adherence to treatment in children and adolescents with cystic fibrosis: a cross-sectional, multi-method study investigating the influence of beliefs about treatment and parental depressive symptoms. BMC Pulm Med. 2015;15:43.

• Grasso MC, Button BM, Allison DJ, Sawyer SM. Benefits of Music Therapy as an Adjunct to Chest Physiotherapy in Infants and Toddlers With Cystic Fibrosis. Pediatric Pulmonology 2000. 29:371–381.

• Irons JY, Petocz P, Kenny DT, Chang AB. Singing as an adjunct therapy for children and adults with cystic fibrosis. Cochrane Database Syst Rev. 2014.(6):CD008036.

• Kriemler S, Radtke T, Christen G, Kerstan-Huber M, Hebestreit H. Short-Term Effect of Different Physical Exercises and Physiotherapy Combinations on Sputum Expectoration, Oxygen Saturation, and Lung Function in Young Patients with Cystic Fibrosis. Lung. 2016 May 4. [Epub ahead of print]:1- 6.

• Le Roux FH, Bouic, PJ ;Bester, MM. The Effect of Bach's Magnificat on Emotions, Immune, and Endocrine Parameters During Physiotherapy Treatment of Patients with Infectious Lung Conditions. Journal of Music Therapy. 2007. 44, 2;156 -168.

• Main E, Prasad A, Van der Schans CP. Conventional Cheste physiotherapy compared to other airway clearance techniques for cystic fibrosis. Cochrane Database of Systematic Reviews 2005, Issue 1. Art No..CD 002011.

• McIlwaine MP, Lee Son NM, Richmond ML. Physiotherapy and cystic fibrosis: what is the evidence base?. Curr Opin Pulm Med. 2014;20:613-7.

• Modi AC, Quittner AL. Barriers to treatment adherence for children with cystic fibrosis and asthma: what gets in the way? J Pediatr Psychol. 2006;31:846–58.

• Panigrahi A, Sohani S, Amadi C, Joshi A. Role of music in the management of chronic obstructive pulmonary disease (COPD): a literature review, Technol. Health Care 2014; 2:53 - 61.

• Pisi G, Chetta A. Airway clearance therapy in cystic fibrosis patients. 2009; 80: 102-106.

• Prados C, Máiz L, Antelo C, Baranda F, Blázquez J, Borro JM, Gartnerg S, Garzón G, Girón R, de Gracia J, Lago J, Lama R, Martínez MT, Moreno A, Oliveira C, Pérez-Frías J, A Solé A, Salcedo A. Fibrosis quística: consenso sobre el tratamiento del neumotórax y de la hemoptisis masiva y sobre las indicaciones del trasplante pulmonar. Arch Bronconeumol. 2000;36:411-6.

• Quittner AL, Sweeny S, Watrous M, Munzenberger P, Bearss K., Gibson Nitza A, Fisher L,

Henry B. Translation and linguistic validation of a disease-specific quality of life measure for

cystic fibrosis. Journal of PediatricPsychology. 2000; 25: 403-14.

• Sabaté E. Adherence to long-term therapies: evidence for action. Geneva: World Health Organisation; 2003.

• Smith BA, Modi AC, Quittner AL, Wood BL. Depressive symptoms in children with cystic fibrosis and parents and its effects on adherence to airway clearance. Pediatr Pulmonol. 2010;45:756–63.

ANEXO - CUADERNO DE RECOGIDA DE DATOS

Edad:

Sexo:

Exacerbaciones respiratorias que requieren hospitalización:

	Previo al estudio	Durante el estudio
Nº exacerbaciones		
Días de hospitalización por exacerbación		

<u>CUESTIONARIOS:</u>

 Características del desarrollo de la fisioterapia respiratoria:
- Complemento de la rutina de fisioterapia con algo (SI /NO)
- Uso de complementos durante la fisioterapia respiratoria (juguetes, cuentos, música, radio, TV…)

 Adherencia fisioterapia respiratoria:
- Frecuencia de realización fisioterapia
- Número de veces al día
- Interrupciones en su desarrollo (SI /NO)
- Duración de cada sesión

 Actitud hacia la fisioterapia respiratoria:
- Definición de la fisioterapia respiratoria como desagradable - neutra - agradable según escala de Likert (-3 a +3):
- Sentimientos hacia la rutina de fisioterapia respiratoria según elección de 3 palabras

 Percepción del tiempo para la realización de la fisioterapia respiratoria:
- Percepción de ser una rutina larga (SI /NO)
- Tiempo que le parece necesitar para realizarla

 Sobre la música:
- Frecuencia de utilización de la música
- Respuesta a la utilización de la música durante la rutina de fisioterapia respiratoria como desagradable, neutra, agradable según escala de Likert (-3 a +3)
- Utilidad de la música en la rutina de fisioterapia (SI /NO)
- Manera en que ha sido útil la música
- Continuación de la utilización de esta música como complemento a la fisioterapia respiratoria (SI /NO)

 Percepción sintomatología pulmonar e impacto sobre la calidad de vida:

- Sensación pulmonar definida como dificultad para respirar según escala visual analógica (EVA) para evaluar la magnitud de la disnea donde 1 significa no falta de aire y 7 máxima falta de aire

- Sintomatología respiratoria y características según cuestionario revisado de calidad de vida para FQ: Frecuencia durante las 2 semanas anteriores:

siempre, a menudo, a veces, nunca

Tos durante el día ....
Despertar durante la noche por toser ....
Escupir (expectorar) mucosidad ....
Mucosidad mayormente: Transparente Transparente a amarilla Amarillosa-verdosa Verde con muestras de sangre No sé
Congestionado(a) ....
Pitos al respirar ....
Dificultad al respirar ....

CONSENTIMIENTO INFORMADO – INFORMACIÓN AL PACIENTE

Efectos de la musicoterapia como complemento de la fisioterapia respiratoria en pacientes con fibrosis quística.

Antes de proceder a la firma de este consentimiento informado, lea atentamente la información que a continuación se le facilita y realice las preguntas que considere oportunas.

Naturaleza:

Las técnicas de drenaje bronquial que se utilizan de 1 a 2 veces al día en las personas que tienen fibrosis quística son tratamientos que ayudan a estas personas a mantenerse sanas y puedan respirar con más facilidad. Estas técnicas de fisioterapia respiratoria aflojan el moco espeso y pegajoso, para que pueda sacarse tosiendo o soplando. La limpieza de las vías respiratorias reduce las infecciones pulmonares y mejora la función pulmonar.

Estás técnicas suponen un compromiso significativo de tiempo y energía para el paciente y sus familiares en el caso de los niños que no pueden realizarla por sí mismos por su corta edad. Por eso aunque son muy beneficiosas, se convierten en una rutina aburrida.

Importancia:

Consideramos que convertir la fisioterapia respiratoria en una actividad más divertida para los niños y sus familiares es muy importante para su correcta realización.

Este estudio es un ensayo clínico sin intervención farmacológica donde el método que asigna a los pacientes a uno u otro brazo de estudio es el azar. A un grupo de niños con fibrosis quística le proponemos realizar su rutina de fisioterapia respiratoria escuchando canciones que un músico ha compuesto especialmente para oírla durante el desarrollo de esta fisioterapia. A un segundo grupo de niños con fibrosis quística se le propondrá complementar su rutina de fisioterapia respiratoria con música comercial elegida por el propio paciente. Y en un tercer grupo de estudio los niños con fibrosis quística continuarán realizando su fisioterapia respiratoria de forma habitual, es decir, sin complementar con música.

La música terapéutica compuesta especialmente para acompañar al desarrollo de la fisioterapia respiratoria tiene 3 partes, las mismas que su rutina de fisioterapia respiratoria:

1. Tratamiento nebulizador
2. Trabajo de fisioterapia - limpieza bronquial
3. Relajación-nebulización

Los dos grupos que van realizar la fisioterapia respiratoria escuchando música deben de adaptar su fisioterapia habitual a la música y no al revés. Solo queremos que la música complemente la actividad.

En este periodo, independientemente al grupo que pertenezca, le realizaremos 3 entrevistas sobre su experiencia en general con la fisioterapia respiratoria: antes de comenzar, a las 6 semanas y al finalizar el estudio. El estudio en concreto durará 3 meses.

Además de la información obtenida sobre las entrevistas, se recogerán datos habituales incluidos en su historia médica, en concreto datos relacionados con su sintomatología pulmonar.

Todos los datos obtenidos se incluirán en una base de datos anónima codificada por lo que no es posible identificar a los pacientes. En ningún informe del estudio aparecerá su nombre, y su identidad no podrá ser conocida salvo requerimiento legal. Los resultados del estudio podrán ser comunicados a las autoridades sanitarias y, eventualmente, a la comunidad científica a través de congresos y/o publicaciones.

Durante el estudio, debe continuar con su régimen habitual de tratamiento para la fibrosis quística sin alterarlo. Debe de saber, que la participación en este estudio, no supondrá la aplicación de técnicas de monitorización adicionales ni la aplicación de ninguna medida que pueda dañarle o molestarle, ni la extracción de muestras biológicas extra, ni alteraciones en su tratamiento y seguimiento.

Tras estos 3 meses, a aquellos pacientes pertenecientes a los grupos que no han utilizado esta música terapéutica se les ofrecerá la música para que si lo desean complementen su rutina de fisioterapia respiratoria con esta música.

Implicaciones para el paciente:

- La participación es totalmente voluntaria.
- El paciente puede retirarse del estudio cuando así lo manifieste, sin dar explicaciones y sin que esto repercuta en sus cuidados médicos.
- Todos los datos de carácter personal, obtenidos en este estudio son confidenciales y se tratarán conforme a la Ley Orgánica de Protección de Datos de Carácter Personal 15/99.
- La información obtenida se utilizará exclusivamente para los fines específicos de este estudio.

Riesgos de la investigación para el paciente:

Este estudio no supone riesgo para el paciente, pues solo se trata de complementar la rutina de fisioterapia respiratoria habitual del paciente con música, lo que no supone ni un incremento en el número de visitas a la unidad para estos pacientes ni una alteración en su tratamiento ni en su seguimiento habitual.

Si usted tiene alguna duda o requiere información adicional se puede poner en contacto con nuestro personal de la Unidad de Fibrosis Quística en el teléfono: 951292187 o en el correo electrónico: emartinm@uma.es

CONSENTIMIENTO INFORMADO – CONSENTIMIENTO POR ESCRITO DEL PACIENTE

Efectos de la musicoterapia como complemento de la fisioterapia respiratoria en pacientes con fibrosis quística.

Yo (Nombre y Apellidos):..

- He leído el documento informativo que acompaña a este consentimiento (Información al Paciente)
- He podido hacer preguntas sobre el estudio *Efectos de la musicoterapia como complemento de la fisioterapia respiratoria en pacientes con fibrosis quística.*
- He recibido suficiente información sobre el estudio *Efectos de la musicoterapia como complemento de la fisioterapia respiratoria en pacientes con fibrosis quística.* He hablado con el profesional sanitario informador: Dr. Javier Pérez Frías.
- Comprendo que mi participación es voluntaria y soy libre de participar o no en el estudio.
- Se me ha informado que todos los datos obtenidos en este estudio serán confidenciales y se tratarán conforme establece la Ley Orgánica de Protección de Datos de Carácter Personal 15/99.
- Se me ha informado de que la información obtenida sólo se utilizará para los fines específicos del estudio.
- **Deseo** ser informado/a de mis datos genéticos y otros de carácter personal que se obtengan en el curso de la investigación, incluidos los descubrimientos inesperados que se puedan producir, siempre que esta información sea necesaria para evitar un grave perjuicio para mi salud o la de mis familiares biológicos.

Si No

Comprendo que puedo retirarme del estudio:

- Cuando quiera
- Sin tener que dar explicaciones
- Sin que esto repercuta en mis cuidados médicos

Presto libremente mi conformidad para participar en el *proyecto titulado Efectos de la musicoterapia como complemento de la fisioterapia respiratoria en pacientes con fibrosis quística.*

Firma del paciente<br>(mayor de 12 años)	Firma de los padres<br>(o representante legal)	Firma del profesional<br>sanitario informador
Nombre y apellidos:<br>………………..........	Nombre y apellidos:<br>…………………………..	Nombre y apellidos:<br>……………………………….
Fecha: ………………	Fecha: …………………..	Fecha: ……………………….

CONSENTIMIENTO INFORMADO ADAPTADO A MENORES – INFORMACIÓN AL PACIENTE

Efectos de la musicoterapia como complemento de la fisioterapia respiratoria en pacientes con fibrosis quística.

Antes de proceder a la firma de este consentimiento informado, lee atentamente la información que a continuación se te facilita y realiza las preguntas que consideres oportunas.

Naturaleza:

El Dr. Javier Pérez Frías me ha informado de que en los niños con fibrosis quística las técnicas de drenaje bronquial como las que debo realizar yo de 1 a 2 veces al día me ayudan a respirar mejor y sentirme más sano. Estas técnicas de fisioterapia respiratoria aflojan el moco espeso y pegajoso, para que pueda sacarlo tosiendo o soplando. La limpieza de las vías respiratorias hace que tenga menos infecciones en los pulmones y mejore la función de mis pulmones.

Estás técnicas suelen cansar al paciente y ocupan mucho tiempo del día, por eso, aunque ayudan a respirar mejor pueden ser aburridas. Pensamos que convertir estas técnicas en una actividad más divertida puede ayudarte a realizarlas correctamente por ejemplo acompañando las sesiones con música.

Metodología empleada para el estudio:

-¿Cómo se realizará el estudio?

Se realiza con niños que como yo tienen fibrosis quística y hacen técnicas de drenaje bronquial cada día. A unos niños se les dirá que hagan la fisioterapia respiratoria escuchando unas canciones que un músico ha compuesto especialmente para nosotros. A otros se les pedirá que hagan la fisioterapia respiratoria escuchando la música que les guste y a otros que la hagan como habitualmente la realizan, es decir, sin música. Se elegirá "a suertes" qué niño realizará la fisioterapia con las canciones que el músico ha compuesto (grupo 1), qué niño la hará escuchando la música que le guste (grupo 2) y qué niño la realizará sin música (grupo 3). No cambiarán nada los tratamientos o cuidados que recibirán todos los niños.

-¿En qué consistirán las intervenciones?

A los niños que participen en el estudio se les pedirá que mientras se ponen el aerosol, cuando intentan sacar el moco y cuando vuelven a ponerse el aerosol cada día durante 3 meses escuchen la música que el músico ha compuesto especialmente para ellos (si pertenecen al grupo 1), escuchen la música que les guste (si pertenecen al grupo 2) y no escuchen música (si pertenecen al grupo 3).

Los dos grupos que van realizar la fisioterapia respiratoria escuchando música deben de adaptar los ejercicios a la música y no al revés. Solo queremos que escuches la música pero que esto no modifique las sesiones de fisioterapia respiratoria.

Durante el estudio, los niños deben continuar con su tratamiento para la fibrosis quística sin alterarlo. Participar en este estudio, no supondrá la aplicación de técnicas de monitorización adicionales ni la aplicación de ninguna medida que pueda dañarte o molestarte, ni la extracción de muestras de sangre extra, ni alteraciones en tu tratamiento y seguimiento. No hay nada nuevo ni ninguna prueba salvo la música.

A los pacientes de los tres grupos se les harán 3 entrevistas sobre cómo hacen la fisioterapia respiratoria y cómo se sienten. La primera entrevista antes de comenzar, la segunda a las 6 semanas y la tercera a los 3 meses que es cuando finaliza el estudio.

Cuando finalice el estudio, a aquellos niños de los grupos que han realizado la fisioterapia respiratoria escuchando la música que les guste o sin música, se les ofrecerá la música que el músico ha compuesto para los niños con fibrosis quística para que si quieren, la utilicen.

-¿Qué datos se recogerán?

Además de la información obtenida sobre las entrevistas, se recogerán datos habituales incluidos en tu historia médica, en concreto datos relacionados con tu sintomatología pulmonar.

-¿Supondrá algún riesgo para el niño?

Este estudio no supone riesgo pues solo se trata de escuchar música durante la realización de fisioterapia respiratoria, lo que no supone, ni un incremento en el número de visitas al médico, ni una alteración en tu tratamiento ni en tu seguimiento habitual.

-¿Cómo se garantizará el anonimato de los pacientes?

Todos los datos obtenidos se incluirán en una base de datos en la que no aparece tu nombre, si no un código, por lo que no es posible identificarte. En ningún informe del estudio aparecerá tu nombre, y tu identidad no podrá ser conocida salvo requerimiento legal. Los resultados del estudio podrán ser comunicados a través de congresos, informes etc. pero nunca con tu nombre ni datos personales.

Implicaciones para el paciente:

- La participación es totalmente voluntaria.
- Puedes retirarte del estudio cuando quieras, sin dar explicaciones y sin que esto repercuta en tus cuidados médicos.
- Todos los datos de carácter personal, obtenidos en este estudio son confidenciales y se tratarán conforme a la Ley Orgánica de Protección de Datos de Carácter Personal 15/99.
- La información obtenida se utilizará exclusivamente para los fines específicos de este estudio.

Si tienes alguna duda o necesitas información adicional puedes ponerte en contacto con nuestro personal de la Unidad de Fibrosis Quística en el teléfono: 951292187 o en el correo electrónico: emartinm@uma.es

CONSENTIMIENTO INFORMADO – CONSENTIMIENTO POR ESCRITO DEL PACIENTE

Efectos de la musicoterapia como complemento de la fisioterapia respiratoria en pacientes con fibrosis quística.

Yo (Nombre y Apellidos):...

- He leído el documento informativo que acompaña a este consentimiento (Información al Paciente)
- He podido hacer preguntas sobre el estudio *Efectos de la musicoterapia como complemento de la fisioterapia respiratoria en pacientes con fibrosis quística.*
- He recibido suficiente información sobre el estudio *Efectos de la musicoterapia como complemento de la fisioterapia respiratoria en pacientes con fibrosis quística.* He hablado con el profesional sanitario informador: Dr. Javier Pérez Frías.
- Comprendo que mi participación es voluntaria y soy libre de participar o no en el estudio.
- Se me ha informado que todos los datos obtenidos en este estudio serán confidenciales y se tratarán conforme establece la Ley Orgánica de Protección de Datos de Carácter Personal 15/99.
- Se me ha informado de que la información obtenida sólo se utilizará para los fines específicos del estudio.
- **Deseo** ser informado/a de mis datos genéticos y otros de carácter personal que se obtengan en el curso de la investigación, incluidos los descubrimientos inesperados que se puedan producir, siempre que esta información sea necesaria para evitar un grave perjuicio para mi salud o la de mis familiares biológicos.

Si No

Comprendo que puedo retirarme del estudio:

- Cuando quiera
- Sin tener que dar explicaciones
- Sin que esto repercuta en mis cuidados médicos

Presto libremente mi conformidad para participar en el *proyecto titulado Efectos de la musicoterapia como complemento de la fisioterapia respiratoria en pacientes con fibrosis quística.*

Firma del paciente<br>(mayor de 12 años)	Firma de los padres<br>(o representante legal)	Firma del profesional<br>sanitario informador
Nombre y apellidos:<br>………………..........	Nombre y apellidos:<br>…………………………..	Nombre y apellidos:<br>……………………………….
Fecha: ………………	Fecha: …………………..	Fecha: ……………………….
