# Supplementary material for: Effects of music therapy as an adjunct to chest physiotherapy in children with cystic fibrosis: A randomized controlled trial
Source: PLoS One. 2020 Oct 30;15(10):e0241334. doi: 10.1371/journal.pone.0241334 (PMC7598495; doi:10.1371/journal.pone.0241334)
Supplement: S3 File — (PDF) [file pone.0241334.s003.pdf]

JUNTA DE ANDALUCIA
CONSEJERÍA DE SALUD

TRIAL PROTOCOL

Tittle:
Effects of music therapy as complement of chest physiotherapy in patients with cystic fibrosis

Principal investigator:
Elisa Martín Montañez, PhD. Department of Pharmacology and Pediatrics, Faculty of Medicine, Malaga University, Biomedical Research Institute of Malaga (IBIMA), Malaga, Spain.

Collaborating researchers:
Javier Pérez Frías, MD, PhD. Department of Pharmacology and Pediatrics, Faculty of Medicine, Malaga University. Biomedical Research Institute of Malaga (IBIMA), Malaga, Spain. Unit of Pediatric Pulmonology, Malaga Regional Hospital, Malaga, Spain.

Laura A. Fuentes Gálvez, PhD. Physiotherapist specialist in children chest physiotherapy. Department of Pharmacology and Pediatrics, Faculty of Medicine, Malaga University, Biomedical Research Institute of Malaga (IBIMA), Malaga, Spain.

Alberto Montero Ruiz, PhD. Professional musician and music teacher of children. Department of Pharmacology and Pediatrics, Faculty of Medicine, Malaga University. Biomedical Research Institute of Malaga (IBIMA), Malaga, Spain. Department of Education. Andalusian Government, Malaga, Spain.

Objective: To develop a strategy based on music therapy as adjunct to daily chest physiotherapy in children with cystic fibrosis and to evaluate its effects.

Study design: Interventional - randomized controlled trial. Single center: Unit of Pediatric Pulmonology, Malaga Regional Hospital.

Disease or disorder under study: Cystic fibrosis.

Participants: Children between ages of 2-17 from the Pediatric Pulmonology Unit at Malaga Regional Hospital. The sample size determined in each group is 13 participants. A total of 39 participants are necessary.

Total study period: Two years.

BACKGROUND

Cystic fibrosis (CF), is a rare, chronic, multisystemic disease that greatly affects the quality of life. A progressive lung dysfunction and its clinical manifestations are found in 95% of patients (CFF, 2014). These clinical manifestations are mainly related to morbidity and mortality (Alexander et al, 2014, CFF, 2014). Thus, the decrease in lung capacity and its consequences contribute to a degeneration of lung tissue, being ultimately the lung transplantation the only solution (Prados et al, 2000, CFF, 2014). Therefore, it is essential to establish an adequate treatment that prevents the progression of the lung disease. Chest physiotherapy (CPT) is one of the main approaches used to improve airway clearance, being essential in the maintenance of pulmonary function in CF due to the excess of respiratory secretions in these patients (McIlwaine et al, 2014).

Chest physiotherapy treats bronchial obstruction through airway clearance techniques administration such as deep breathing, huffing, coughing, percussions or vibrations (Pisi and Chetta, 2009; Main et al, 2011; CFF, 2014). The CPT is prescribed daily, requiring a significant commitment of time and energy for children and family members, what complicates CPT adherence (Modi & Quittner, 2006) and reduces its benefits (Sabaté, 2003; Goodfellow et al. , 2015).

Music exerts a motivational role in chronic diseases leading to psycho-emotional improvement (Le Roux et al, 2007). Music therapy (MT) interventions are usually part of integrative strategies to ameliorate some physical and/or psycho-emotional consequences in lung diseases, case of chronic obstructive pulmonary disease (Bausewein et al, 2013; Panigrahi et al, 2014). Patients reach emotional positive states reducing anxiety and depression with MT (Canga et al, 2015). In this sense, there are few music therapy interventions in CF (Goldbeck et al, 2014; Irons et al, 2014). Recently, it has been described that a carefully selected motivational music can lead to positive affective response during exercise in CF children aged from 8 to 18 (Calik-Kutukcu et al, 2016). There is only one study in CF infants and children under 2 years of age where, an appropriate music created specifically for CPT, helps to establish CPT as a routine by converting it into a positive experience (Grasso et al, 2000).

HYPOTHESIS

The adherence in chronic disease treatment is very low (Sabaté, 2003), being the CPT adherence in CF less than 50 % (Goodfellow et al, 2015). To improve airway clearance in CF patients, there is no therapeutic alternative to CPT, in any case, it has to be combined with physical exercise (Kriemler et al, 2016). Motivation is the best way to optimize treatment adherence (Smith et al, 2010) and the use of carefully selected music during CPT (Grasso et al, 2000) or physical exercise (Calik-Kutukcu et al, 2016) in CF

patients can lead to a positive affective response that could be translated into a improvement in adherence (Smith et al, 2010) and therefore in airway clearance that could maintain the lung function and improve the children's and family member's quality of life.

Baseline hypothesis: Significant differences are expected in chest physiotherapy enjoyment and perception, adherence to daily chest physiotherapy, pulmonary symptomatology and quality of life between participants that use specific composed music as an adjunct to chest physiotherapy routine (intervention group), commercial music as an adjunct to chest physiotherapy routine (control group with music), or no music (control group without music).

OBJECTIVES

Main objective:

To develop a strategy based on music therapy as adjunct to daily chest physiotherapy in children with cystic fibrosis and to evaluate its effects. Instrumental music specifically composed, interpreted and compiled to cystic fibrosis children as an adjunct to chest physiotherapy routine will be used.

Secondary objectives:

• To evaluate the effects of music therapy as an adjunct to chest physiotherapy on enjoyment, perception, adherence to daily chest physiotherapy of patients and family members.

• To evaluate the effects of music therapy as an adjunct to chest physiotherapy on pulmonary symptomatology and quality of life.

METHODOLOGY, TRIAL PLAN AND SCHEDULE

METHODOLOGY

- Study design:

Interventional prospective study on pediatric cystic fibrosis patients. Randomized controlled trial.

- Scope:

Single center: Unit of Pediatric Pulmonology, Malaga Regional Hospital, Malaga, Spain.

- Participants:

Children from the Pediatric Pulmonology Unit at Malaga Regional Hospital who meet all of the following inclusion criteria and none of the exclusion criteria:

- Inclusion criteria:

- Diagnosis of cystic fibrosis based on international criteria (Aldana et al, 2011).
- Children between ages of 2-17.
- Undergoing periodic clinic visits in the cystic fibrosis Unit.
- Understanding the purpose of the study.
- To provide written informed consent.

- Exclusion criteria:

- Children without chest physiotherapy prescription.
- Children with severe hearing loss.
- Children at radiologic or clinical risk of pneumothorax or pneumomediastinum.
- Children with barotrauma in the month prior to entry in the study.
- Children with past history of massive or life-threatening haemoptysis.
- Transplant recipients or children awaiting a lung transplant.

Children / legal guardian must have understood the purpose of the study and express their agreement by signing the informed consent prior to the inclusion in the study (model attached).

- Sample size:

To calculate the sample size, the perception of time taken to complete the routine has been assumed as main variable. According to Grasso et al, (2000) where the control group values in this variable were 0.2±10.2 (mean± SD), considering these values as baseline, a power of 80 %, a confidence level of 95 %, and a similar variability at the end of the study, a difference of 8 minutes would be considered as statistically significant with a sample size of 13 subjects. A total of 39 participants are necessary.

- Randomization:

After providing written informed consent, a random allocation sequence will be carried out using the Epidat program. The participants will be randomly allocated into intervention group, control group without music, or control group with music.

The groups are the followed:

- *Intervention group or TG* (participants that use specific composed music as an adjunct to chest physiotherapy routine).
- *Control group without music or CG* (participants that do not use music as an adjunct to chest physiotherapy routine).
- *Control group with music or PG* (participants that use commercial music as an adjunct to chest physiotherapy routine).

Participants in every group will continue with their usual treatment regimen without modifications.

- Data collection:

Demographic and clinical data will be collected in the case report formulary from each participant (Annex). Specifically: age, gender and respiratory infection exacerbations that requiring hospitalization.

These participants are controlled in the Pediatric Pulmonology Unit at Malaga Regional Hospital, where each child with cystic fibrosis has a detailed clinic history, being the clinical variables systematically included and the treatment updated. Therefore, the present study does not imply additional clinic information collection. Demographic and clinical information will be completed by Pediatricians during the usual follow-up.

In addition, participants will complete a baseline and 2 evaluation questionnaires during the trial-period in order to know children's and family members' chest physiotherapy perception. The questionnaires will be conducted about chest physiotherapy experiences to evaluate the evolution of their perceptions during the trial-period. These questionnaires are designed specifically to know evolution of CPT characteristics such as, enjoyment and perception or time or adherence to daily chest physiotherapy, after the use of music therapy as an adjunct to CPT (Grasso et al, 2000). The perception of pulmonary symptomatology and the impact on the quality of life will be also evaluate using a visual analog scale (VAS) to assess the magnitude of dyspnea in pulmonary diseases such as CF (Bausewein et al, 2007) and the revised cystic fibrosis quality of life questionnaire (Quittner et al, 2000), respectively.

- Study variables:

- *Demographics*:
  - Age (quantitative variable) (unit: years)
  - Gender (qualitative, dichotomous variable) (male / female)

- *Clinics*:
  - Number of respiratory infection exacerbations that requiring hospitalization (quantitative variable)
  - Days of hospitalization per exacerbation (quantitative variable)

- *Chest physiotherapy characteristics*:
  - Use of activities to accompany the routine (qualitative, dichotomous variable) (yes /no)
  - Type of activities used to accompany the routine (toys, stories, music, radio, TV…) (qualitative variable)

- *Chest physiotherapy adherence*:
  - Routine frequency (quantitative variable)

- Number of times per day (quantitative variable)
- Interruptions (qualitative, dichotomous variable) (yes /no)
- Length per session (quantitative variable)

- *Chest physiotherapy attitude*:
  - Response to the routine using a Likert scale (-3 to +3): least enjoyment – neutral - most enjoyment (quantitative variable)
  - Feelings about the routine choosing 3 words to describe these feelings (qualitative variable)

- *Perception of time taken to complete the chest physiotherapy*:
  - Perception of being a long routine (qualitative, dichotomous variable) (yes /no)
  - Time that seems to need the routine (quantitative variable)

- *About the music*:
  - Music use frequency (quantitative variable)
  - Response to the use of music during routine using a Likert scale (-3 to +3): least enjoyment – neutral - most enjoyment (quantitative variable)
  - Usefulness of music during routine (qualitative, dichotomous variable) (yes /no)
  - How music has been useful (qualitative variable)
  - To continue using this music as an adjunct to the routine in the future (qualitative, dichotomous variable) (yes /no)

- *Perception of pulmonary symptomatology and impact on quality of life*:
  - Measurement of the symptoms of dyspnea using a dyspnea visual analog scale (VAS), being 1 no dyspnea and 7 maximal dyspnea.

- Frequency of respiratory symptoms according to the revised quality of life questionnaire for cystic fibrosis during the previous 2 weeks: cough during the day and night, expectoration and type of expectoration, breathing difficulties, whistling sounds when breathing, and respiratory congestion.

- Development of the instrumental therapeutic music:

An instrumental therapeutic music will be developed specifically to the CF children from the Pediatric Pulmonology Unit at Malaga Regional Hospital to use as an adjunct to each part of the CPT routine according to the CPT strategy of this Unit.

The CPT treatment in this Unit is divided into 3 sections with a total length of about 40 minutes: During the first part, children should be relaxed while the nebulizer inhalation treatment is applied around 10-15 minutes. Next, airway clearance techniques are administered during 20-30 minutes to promote mucus expulsion. Finally, a new relaxation phase of about 5 minutes is proposed, where antibiotic nebulizer treatment is administered if necessary.

The music will be divided into 3 sections related to the 3 parts of the CPT routine:

- Section A: nebulizer treatment
- Section B: CPT work-bronchial clearance
- Section C: relaxation-nebulization

The professional musician will compose, and score-write the different music pieces according to the clinical recommendations from the research group. The composition of about 10 pieces is estimated.

After that, the musician with higher percussion studies will perform the songs with pitched percussion instruments (marimba, vibes, glockenspiel and xylophone) and unpitched percussion instruments (drums, congas, bongos, multi-percussion set and small-percussion instruments).

To record the audio, a portable studio (Tascam) and specific microphones will be used. Subsequently, the audio will be edited with the Cakewalk Sonar Platinum edition software and complied in an audio-CD that will be use by participants in the intervention group.

- Intervention:

After participant recruitment they will complete the baseline questionnaire and be asked to carry out the CPT routine during 6 weeks (Grasso et al, 2000; Canga et al, 2015) as follow:

- *Intervention group or TG*: Using the Music-CD as an adjunct to each part of the CPT routine. The Music-CD will be provided after baseline questionnaire.
- *Control group without music or CG*: Continuing with the usual CPT without modifications.
- *Control group with music or PG:* Using commercial music as an adjunct to each part of the CPT routine.

As has been comment, the CPT treatment in this Unit is divided into 3 sections with a total length of about 40 minutes: During the first part, children should be relaxed while the nebulizer inhalation treatment is applied around 10-15 minutes. Next, airway clearance techniques are administered during 20-30 minutes to promote mucus expulsion. Finally, a new relaxation phase of about 5 minutes is proposed, where antibiotic nebulizer treatment is administered if necessary.

Participants in every group will continue with their usual treatment regimen without modifications. Follow-up changes or treatments modifications are not planned in any case.

A second questionnaire will be completed after 6 weeks and participants will continue managing the CPT routine in the same way during a new 6-week period. After that, the final questionnaire will be completed (Grasso et al, 2000) and the music-CD will be offered to CG and PG participants.

- Statistical analysis:
Statistical analysis will be performed using SPSS Software.

Initially a descriptive analysis of the study variables will be carried out, the values of the continuous variables will be summarized in a table where their corresponding means and standard deviation will be shown. The categorical variables will be presented in absolute and relative frequencies.

To compare qualitative measures Chi-square or Fisher tests will be used, and ANOVA or Kruskal-Wallis tests to quantitative.

To contrast the differences in the evolution of the variables between the different groups the multivariate analysis of variance test of repeated measures (MANOVA) will be applied with a factor between subjects (group) and a factor intra subjects (different moments of the intervention). Depending on the compliance of the sphericity condition, the Greenhouse-Geisser correction will be used. To contrast the differences between baseline and final measures in each group Wilcoxon test will be applied. To compare response to using music after the intervention into TG and PG, Mann–Whitney U test was used.

To compare response to using music after the intervention into TG and PG, Mann–Whitney U test will be used.

A significance of 5% ($p<0.05$) will be required to consider a difference as statistically significant.

- Limitations:
Selection bias, people who decline to participate in the study can introduce a selection bias, this can be controlled in the data analysis phase, analyzing if there are differences between people who participate and people who do not.

TRIAL PLAN

The research group will meet to define the instrumental music that will be composed to CF children as an adjunct to each part of CPT routine. After that, the musician member of the research group will compose and score-write the musical pieces. Then, he will interpret these songs playing percussion instruments and record the pieces.

The research group will supervise and select the adequate songs to each part of CPT and then, the musician will edit the musical pieces selected according to the clinical recommendations from the Pulmonology Unit and the Physiotherapist specialist in children CPT.

The music chosen will be compiled in an audio-CD and, after that moment, the recruitment and the intervention will be started and carried out as described above.

Demographic and clinical data will be collected and the questionnaires fill in as mentioned before.

Finally trial data will be analyzed systematically and diffused.

SCHEDULE

Tasks	Year 1												Year 2											
**Months**	1	2	3	4	5	6	7	8	9	10	11	12	1	2	3	4	5	6	7	8	9	10	11	12
Initial meeting and definition of the music to be developed																								
Music composition/ Music scores-writing																								
Interpretation/Recording of musical pieces																								
Musical pieces supervising and selection																								
Edition of the musical pieces selected																								
Study analysis																								
Music-CD compilation																								
Recruitment																								
Demographic and clinical data collection																								
Filled in questionnaires																								
Statistical analysis																								

ETHICAL CONSIDERATIONS

The interventions associated with each group are not related with the use of investigational drugs or devices. The aim of this trial is to compare the usual CPT routine prescribed in CF patients with the use of commercial music or an instrumental music specifically composed, interpreted and compiled to CF children as an adjunct to each part of CPT routine without modifying the usual treatment regimens or programmed clinic visits. This music therapy intervention could help to establish CPT as a positive routine that could improve its adherence and, therefore, the airway clearance. It could maintain the lung function and improve the children's and family member's quality of life.

The safety profile in each group of study: intervention group (music-CD as an adjunct to chest physiotherapy routine), control group without music (usual chest physiotherapy routine) and control group with music (commercial music as an adjunct to chest physiotherapy routine) is favorable, being optimum the benefit / risk balance.

At the end of the trial period, the music-CD will be offered to both control groups (CG and PG participants).

On the other hand, this music therapy intervention does not presents risks for CF participants due to the intervention consists of using the music as an adjunct to usual CPT.

The study will be conducted according to the Declaration of Helsinki and the Spanish and regional protocols concerning to ethics in human research.

The data confidentiality is assured according to the Spanish protection of personal data 15/1999 Law and the basic law 41/2002 that regulates patient autonomy and rights and obligations in terms of information and clinical documentation.

All the researchers involved in the project are committed to preserve the confidentiality of the information provided by participants.

The study has been applied to the Ethics in Human Research Committee of Malaga Regional Hospital "CEI Provincial de Málaga" to be approved.

Participants (children / legal guardian) must have known and understood the purpose of the study and express their agreement by signing the informed consent prior to the inclusion in the study (model attached). The participation is voluntary.

APPLICABILITY

The music therapy strategy proposed in this project has a direct applicability in healthcare practice due to it is expected an improvement in chest physiotherapy enjoyment and perception, adherence to daily chest physiotherapy, pulmonary symptomatology and quality of life between participants that use specific composed music as an adjunct to chest physiotherapy routine without modifying their treatments and follow-up. These will lead to a reduction in the burden of the disease, which can be translated into improvements in Healthcare systems.

REFERENCES

• Aldana JM, León MT, Salamanca C, Olveira C, Olveira G, Pérez-Frías J, Pérez E, Sierra C, Dapena FJ, Quintana E. Guía asistencial de fibrosis quística. Servicio Andaluz de Salud. Consejería de Salud. Junta de Andalucía. 2011.

• Alexander S, Alshafi K, Anderson AK, Balfour-Lynn I, Bentley S, Buchdahl R, et al. Clinical guidelines for the care of children with cystic fibrosis. Royal Brompton Hospital Paediatric Cystic Fibrosis Team. 2014; 6th edition. Disponible en: www.rbht.nhs.uk/childrencf

• Bausewein C, Farquhar M, Booth S, Gysels M, Higginson IJ. Measurement of breathlessness in advanced disease: a systematic review. Respir Med. 2007. 101:399-410.

• Bausewein C, Booth S, Gysels M, Higginson IJ. Non-pharmacological interventions for breathlessness in advanced stages of malignant and nonmalignant diseases. Cochrane Syst. Rev. 11 (2013) CD005623.

• Calik-Kutukcu E, Saglam M, Vardar-Yagli N, Cakmak A, Inal-Ince D, Bozdemir-Ozel C, Sonbahar-Ulu H, Arikan H, Yalcin E, Karakaya J. Listening to motivational music while walking elicits more positive affective response in patients with cystic fibrosis. Complement Ther Clin Pract. 2016;23:52-8.

• Canga B, Azoulay R, Raskin J, Loewy J. AIR: Advances in Respiration e Music therapy in the treatment of chronic pulmonary disease. Respiratory Medicine 109 (2015) 1532e1539.

• CFF, Cystic Fibrosis Foundation Patient registry 2014 annual data report. Cystic Fibrosis Foundation.

• Goldbeck L, Fidika A, Herle M, Quittner AL. Psychological interventions for individuals with cystic fibrosis and their families. Cochrane Database Syst Rev. 2014. (6):CD003148.

• Goodfellow NA, Hawwa AF, Reid AJ, Horne R, Shields MD, McElnay JC. Adherence to treatment in children and adolescents with cystic fibrosis: a cross-sectional, multi-method study investigating the influence of beliefs about treatment and parental depressive symptoms. BMC Pulm Med. 2015;15:43.

• Grasso MC, Button BM, Allison DJ, Sawyer SM. Benefits of Music Therapy as an Adjunct to Chest Physiotherapy in Infants and Toddlers With Cystic Fibrosis. Pediatric Pulmonology 2000. 29:371–381.

• Irons JY, Petocz P, Kenny DT, Chang AB. Singing as an adjunct therapy for children and adults with cystic fibrosis. Cochrane Database Syst Rev. 2014.(6):CD008036.

• Kriemler S, Radtke T, Christen G, Kerstan-Huber M, Hebestreit H. Short-Term Effect of Different Physical Exercises and Physiotherapy Combinations on Sputum Expectoration, Oxygen Saturation, and Lung Function in Young Patients with Cystic Fibrosis. Lung. 2016 May 4. [Epub ahead of print]:1- 6.

• Le Roux FH, Bouic, PJ ;Bester, MM. The Effect of Bach's Magnificat on Emotions, Immune, and Endocrine Parameters During Physiotherapy Treatment of Patients with Infectious Lung Conditions. Journal of Music Therapy. 2007. 44, 2;156 -168.

• Main E, Prasad A, Van der Schans CP. Conventional Cheste physiotherapy compared to other airway clearance techniques for cystic fibrosis. Cochrane Database of Systematic Reviews 2005, Issue 1. Art No..CD 002011.

• McIlwaine MP, Lee Son NM, Richmond ML. Physiotherapy and cystic fibrosis: what is the evidence base?. Curr Opin Pulm Med. 2014;20:613-7.

• Modi AC, Quittner AL. Barriers to treatment adherence for children with cystic fibrosis and asthma: what gets in the way? J Pediatr Psychol. 2006;31:846–58.

• Panigrahi A, Sohani S, Amadi C, Joshi A. Role of music in the management of chronic obstructive pulmonary disease (COPD): a literature review, Technol. Health Care 2014; 2:53 - 61.

• Pisi G, Chetta A. Airway clearance therapy in cystic fibrosis patients. 2009; 80: 102-106.

• Prados C, Máiz L, Antelo C, Baranda F, Blázquez J, Borro JM, Gartnerg S, Garzón G, Girón R, de Gracia J, Lago J, Lama R, Martínez MT, Moreno A, Oliveira C, Pérez-Frías J, A Solé A, Salcedo A. Fibrosis quística: consenso sobre el tratamiento del neumotórax y de la hemoptisis masiva y sobre las indicaciones del trasplante pulmonar. Arch Bronconeumol. 2000;36:411-6.

• Quittner AL, Sweeny S, Watrous M, Munzenberger P, Bearss K., Gibson Nitza A, Fisher L,

Henry B. Translation and linguistic validation of a disease-specific quality of life measure for

cystic fibrosis. Journal of PediatricPsychology. 2000; 25: 403-14.

• Sabaté E. Adherence to long-term therapies: evidence for action. Geneva: World Health Organisation; 2003.

• Smith BA, Modi AC, Quittner AL, Wood BL. Depressive symptoms in children with cystic fibrosis and parents and its effects on adherence to airway clearance. Pediatr Pulmonol. 2010;45:756–63.

ANNEX - CASE REPORT FORMULARY

Age:

Gender:

Respiratory infection exacerbations that requiring hospitalization:

	Pre-study period	Study-period
Number of exacerbations		
Days hospitalized per exacerbation		

QUESTIONNAIRES:

 Chest physiotherapy characteristics:

- Use of activities to accompany the routine (yes /no)
- Type of activities used to accompany the routine (toys, stories, music, radio, TV…)

 Chest physiotherapy adherence:

- Routine frequency
- Number of times per day
- Interruptions (yes /no)
- Length per session

 Chest physiotherapy attitude:

- Response to the routine using a Likert scale (-3 to +3): least enjoyment – neutral - most enjoyment
- Feelings about the routine choosing 3 words to describe these feelings

 Perception of time taken to complete the chest physiotherapy:

- Perception of being a long routine (yes /no)
- Time that seems to need the routine

 About the music:

- Music use frequency
- Response to the use of music during routine using a Likert scale (-3 to +3): least enjoyment – neutral - most enjoyment
- Usefulness of music during routine (yes /no)
- How music has been useful
- To continue using this music as an adjunct to the routine in the future (yes /no)

 Perception of pulmonary symptomatology and impact on quality of life:

- Measurement of the symptoms of dyspnea using a dyspnea visual analog scale (VAS), being 1 no dyspnea and 7 maximal dyspnea

- Frequency of respiratory symptoms according to the revised quality of life questionnaire for cystic fibrosis during the previous 2 weeks:

always, often, sometimes, never
Coughing during the day..
Wake up at night because of coughing..
To expectorate (spit out the mucus)...
Mucus appearance: Watery Yellow/Transparent Yellow-Green Green/Bloody I do not know
Respiratory congestion...
Whistling sounds when breathing...
Breathing difficulties..

INFORMED CONSENT – INFORMATION SHEET

Effects of music therapy as an adjunct to chest physiotherapy in patients with cystic fibrosis.

Before signing this informed consent form, please read carefully the information provided below and ask the questions you consider appropriate.

Background:

Airway clearance techniques used one or twice daily in people with cystic fibrosis are treatments that help these people stay healthy and breathe easier. These chest physiotherapy techniques loosen the thick and sticky mucus, that it is removed coughing or blowing. The airway clearance reduces lung infections and improves lung function.

These techniques require a significant commitment of time and energy for patients and family members, in the case of young children who can not to be actively involved in their own techniques. This is why, even though they are very beneficial, they become a boring routine.

Importance:

We believe that converting chest physiotherapy into a more enjoyable activity for children and family members is very important for its correct management.

This study is a clinical trial without pharmacological intervention where the patients are assigned to one or another study arm at random. We propose that a group of cystic fibrosis children perform their chest physiotherapy routine listening to songs that a musician has composed especially to listen to during the development of this physiotherapy. A second group of children with cystic fibrosis will be proposed to adjunct their chest physiotherapy routine with commercial music chosen by the patient. And in a third study group cystic fibrosis children will continue to perform their chest physiotherapy as usual.

The specific composed music to use as an adjunct to chest physiotherapy has 3 sections, the same sections as your chest physiotherapy routine:

1. Nebulizer treatment
2. Chest physiotherapy work-bronchial clearance
3. Relaxation-nebulization

The two groups that will perform chest physiotherapy listening to music should adapt their usual physiotherapy to music and not the other way around. We just want the music to complement the activity.

In this period, regardless of the group to which you will belong, we will do 3 interviews about your experience in general with chest physiotherapy: before starting, at 6 weeks and at the end of the study. The study will last 3 months.

In addition to the information obtained about the interviews, usual data included in your clinic history will be collected, specifically data related to your pulmonary symptomatology.

Data obtained will be included in a coded anonymous database, being no possible to identify patients. In the reports study your name will not appear, and your identity cannot be known except legal requirement. The study results may be communicated to the health authorities and, eventually, to the scientific community through congresses and / or publications.

During the study, you should continue with your cystic fibrosis usual treatment regimen without modifications. You should know, that participation in this study, will not involve the realization of additional monitoring techniques or any measure that may harm or bother you, or the extraction of extra biological samples, or alterations in treatment and follow-up.

After these 3 months, this therapeutic music will be offered to those patients belonging to the groups that have not used it. If they wish, they will adjunct their chest physiotherapy routine with this music.

Patient implications:

- Participation is entirely voluntary.
- Patients may choose to leave the trial at any time, without explanations and without affecting their medical care.
- Personal data obtained in this study are confidential and will be treated according to the Protection of Personal Data 15/99 Law.
- Information obtained will be used for the specific purposes of this study exclusively.

Patient risks:

This study does not presents risks for patients due to the intervention consists of use the music as an adjunct to usual chest physiotherapy. The intervention not represents an increment in the number of clinic visit to the Unit, nor treatment modifications or usual follow-up.

If you have any questions or require additional information, please contact our staff of the Cystic Fibrosis Unit by telephone: 951292187 or by email: emartinm@uma.es

INFORMED CONSENT – WRITTEN CONSENT FORM

Effects of music therapy as an adjunct to chest physiotherapy in patients with cystic fibrosis.

I (full name):...

- I have read the foregoing information (Information sheet)
- I have had the opportunity to ask questions about the study *Effects of music therapy as an adjunct to chest physiotherapy in patients with cystic fibrosis.*
- I have received enough information about the study *Effects of music therapy as an adjunct to chest physiotherapy in patients with cystic fibrosis.* I have spoken with the healthcare professional informant: Dr. Javier Pérez Frías.
- I understand that my participation is voluntary and I am free to participate or not in the study.
- I have been informed that data obtained in this study will be confidential and will be treated according to the Protection of Personal Data 15/99 Law.
- I have been informed that information obtained will be used for the specific purposes of this study exclusively.
- **I would like to be informed about** my genetic and other personal data obtained during this investigation, including any unexpected discoveries that may occur, provided that this information is necessary to avoid serious damage to my health or my biological relatives health.

Yes No

I understand that I can choose to leave the trial:

- At any time
- Without explanations
- Without affecting my medical care

I am freely accepting my agreement to participate in the project entitled *Effects of music therapy as an adjunct to chest physiotherapy in patients with cystic fibrosis.*

Signature of patient (over the age of 12)	Signature of parents (or legal guardian)	Signature of healthcare professional informant
Full name: ………………...........	Full name: …………………………..	Full name: ……………………………….
Date: ………………	Date: …………………..	Date: ……………………….

INFORMED ASSENT FORM FOR CHILDREN/MINORS – INFORMATION SHEET

Effects of music therapy as an adjunct to chest physiotherapy in patients with cystic fibrosis.

Before signing this informed consent form, please read carefully the information provided below and ask the questions you consider appropriate.

Background:

The Doctor Javier Pérez Frías has informed me that in cystic fibrosis children the airway clearance techniques, such as the techniques that I have to be use once or twice daily, help me to breath better and to feel healthier. These chest physiotherapy techniques loosen the thick and sticky mucus, so I can remove it by coughing or blowing. The airway clearance makes I have less lung infections and improve my lung function.

These techniques tend to tire the patient and they consume a lot of time every day, so even though they help you to breathe better they can be boring. We think that turning these techniques into a funnier activity can help you to use them correctly, for example, if you listen to music during the session.

Methodology used in the study:

- How will the study be done?

It will be done with children who like me have cystic fibrosis and do airway clearance techniques every day. Some children will do chest physiotherapy listening to some songs that a musician has composed especially for us. Others children will do chest physiotherapy listening to the music they like and others children will do chest physiotherapy as usual. Children in each group will be chosen "by lots". So, children belonging to group 1 will do the chest physiotherapy listening to the songs that the musician has composed, children belonging to the group 2 will do it listening to the music that he likes and children belonging to group 3 will do it as usual. Children treatments and cares will not been changed.

- What will the interventions consist of?

Children who participate in the study, during their aerosol administration and when they try to expulse the mucus every day for 3 months, will listen to the music that the musician has composed especially for them (if they belong to the group 1), they will listen to the music they like (if they belong to the group 2), and in the case of group 3, children will not listen to music.

The two groups that will perform chest physiotherapy listening to music should adapt their usual physiotherapy to music and not the other way around. We just want the music to complement the activity.

During the study, children should continue with their cystic fibrosis usual treatment regimen without modifications. You should know, that participation in this study, will not involve the realization of additional monitoring techniques or any measure that may harm or bother you, or the extraction of extra biological samples, or alterations in treatment and follow-up. There is nothing new except music.

Patients will answer 3 interviews about how they do chest physiotherapy and how they feel during the routine. The first interview will be before starting, the second at 6 weeks and the third at the end of the study (after 3 months).

When the study finished, the music specifically composed will be offered to those patients belonging to the groups that have not used it. If they wish, they will do their chest physiotherapy routine with this music.

-What data will be collected?

In addition to the information obtained about the interviews, usual data included in your clinic history will be collected, specifically data related to your pulmonary symptomatology.

-Will it pose any risk to the child?

This study does not present risks for you, due to the intervention consists of listening to music during chest physiotherapy. The intervention not represents an increment in the number of clinic visit to the Unit, nor treatment modifications or usual follow-up.

-How will the anonymity of patients be guaranteed?

Data obtained will be included in a coded anonymous database, being your identification no possible. In the reports study your name will not appear, and your identity cannot be known except legal requirement. The study results may be communicated to the health authorities and, eventually, to the scientific community through congresses and / or publications but without your name or personal data.

Patient implications:

- Participation is entirely voluntary.
- You can choose to leave the trial at any time, without explanations and without affecting your medical care.
- Personal data obtained in this study are confidential and will be treated according to the Protection of Personal Data 15/99 Law.
- Information obtained will be used for the specific purposes of this study exclusively.

If you have any questions or require additional information, please contact our staff of the Cystic Fibrosis Unit by telephone: 951292187 or by email: emartinm@uma.es

INFORMED CONSENT – WRITTEN CONSENT FORM

Effects of music therapy as an adjunct to chest physiotherapy in patients with cystic fibrosis.

I (full name):..

- I have read the foregoing information (Information sheet)
- I have had the opportunity to ask questions about the study *Effects of music therapy as an adjunct to chest physiotherapy in patients with cystic fibrosis.*
- I have received enough information about the study *Effects of music therapy as an adjunct to chest physiotherapy in patients with cystic fibrosis.* I have spoken with the healthcare professional informant: Dr. Javier Pérez Frías.
- I understand that my participation is voluntary and I am free to participate or not in the study.
- I have been informed that data obtained in this study will be confidential and will be treated according to the Protection of Personal Data 15/99 Law.
- I have been informed that information obtained will be used for the specific purposes of this study exclusively.
- **I would like to be informed about** my genetic and other personal data obtained during this investigation, including any unexpected discoveries that may occur, provided that this information is necessary to avoid serious damage to my health or my biological relatives health.

Yes No

I understand that I can choose to leave the trial:

- At any time
- Without explanations
- Without affecting my medical care

I am freely accepting my agreement to participate in the project entitled *Effects of music therapy as an adjunct to chest physiotherapy in patients with cystic fibrosis.*

Signature of patient (over the age of 12)	Signature of parents (or legal guardian)	Signature of healthcare professional informant
Full name: ………………...........	Full name: …………………………..	Full name: ……………………………….
Date: ………………	Date: …………………..	Date: ……………………….
