# Supplementary material for: Effects of music therapy as an adjunct to chest physiotherapy in children with cystic fibrosis: A randomized controlled trial
Source: PLoS One. 2020 Oct 30;15(10):e0241334. doi: 10.1371/journal.pone.0241334 (PMC7598495; doi:10.1371/journal.pone.0241334)
Supplement: S4 File — (DOCX) [file pone.0241334.s004.docx]

| **Section A (nebulizer treatment)** | | | | | | | |
| --- | --- | --- | --- | --- | --- | --- | --- |
| **Songs** | | **Duration** | **Tonality** | **BPM** | **Instruments** | | |
| **Estudio**  **nº 1** | | 04’16’’ | C major | 60 | Drum set, marimba, vibraphone, xylophone, glockenspiel, MPS and small percussion | | |
| **Estudio**  **nº 2** | | 04’24’’ | A minor | 60 | Vibraphone | | |
| **Sagú** | | 02’57’’ | C minor | 65 | Drum set, marimba, vibraphone and Mark tree | | |
| **Tonfarbe** | | 01’19’’ | X | 54 | MPS: gong, Mark tree, tom-toms and cymbals | | |
| **Total duration** | | | 12’56’’ | | **BPM (mean)** | | 59.75 |
| **Section B (ACT work-** **bronchial clearance)** | | | | | | | |
| **Songs** | | **Duration** | **Tonality** | **BPM** | **Instruments** | | |
| **Caibarién** | | 04’09’’ | D minor | 100 | Drum set, congas, bongos, marimba, vibraphone, xylophone, glockenspiel, MPS and small percussion | | |
| **8:00 A.M.** | | 05’24’’ | C minor | 120 | Drum set, congas, bongos, marimba, vibraphone, xylophone, glockenspiel, MPS and small percussion | | |
| **Obstinato Blues** | | 03’47’’ | F major | 130 | Drum set, congas, marimba, vibraphone, xylophone, glockenspiel, MPS and small percussion | | |
| **Idris** | | 03’30’’ | X | 112 | Drum set, congas, bongos, pailas, tom-toms, temple blocks, cowbells and small percussion | | |
| **Leyenda** | | 06’16’’ | F major | 100 | Drum set, congas, marimba, vibraphone, xylophone, glockenspiel, MPS and small percussion | | |
| **Total duration** | | | 23’06’’ | | **BPM (mean)** | | 112.4 |
| **Section C (relaxation-nebulization)** | | | | | | | |
| **Song** | **Duration** | | **Tonality** | **BPM** | **Instruments** | | |
| **Estudio nº 1 (reedited)** | 04’20’’ | | C major | 60 | Vibraphone, marimba, MPS and glockenspiel | | |
| **Total duration** | | | 04’20’’ | | **BPM (mean)** | 60 | |

**S4 File. Music characteristics.**

Abbreviations: MPS, multi-percussion set; BPM, beats-per-minute (number of quarter notes per minute); X, without tonality; ACT, Airway clearance therapy.
