# Supplementary material for: Effects of music therapy as an adjunct to chest physiotherapy in children with cystic fibrosis: A randomized controlled trial
Source: PLoS One. 2020 Oct 30;15(10):e0241334. doi: 10.1371/journal.pone.0241334 (PMC7598495; doi:10.1371/journal.pone.0241334)
Supplement: S5 File — (PDF) [file pone.0241334.s005.pdf]

Estudio nº 1

Swing ballad

Alberto Montero

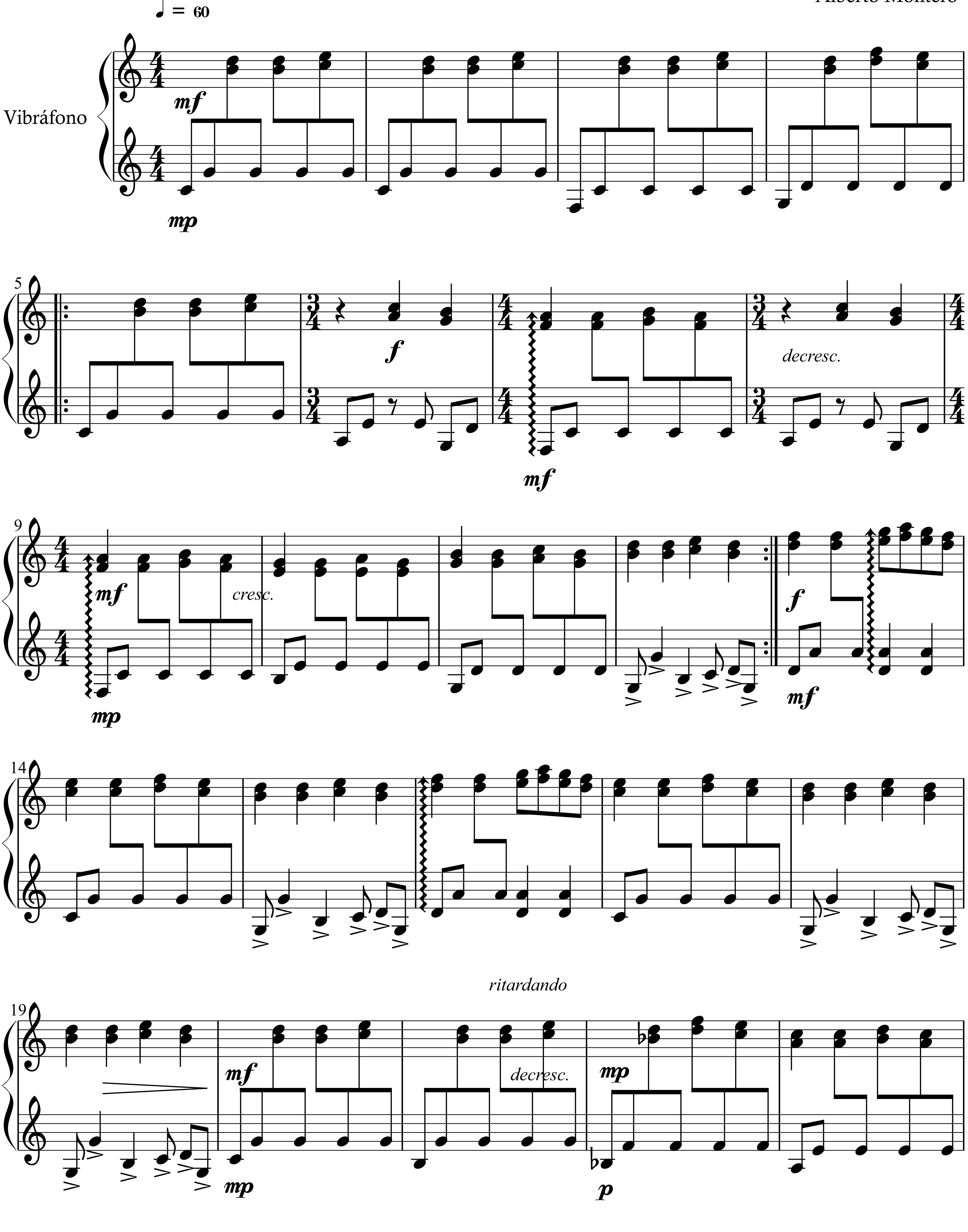

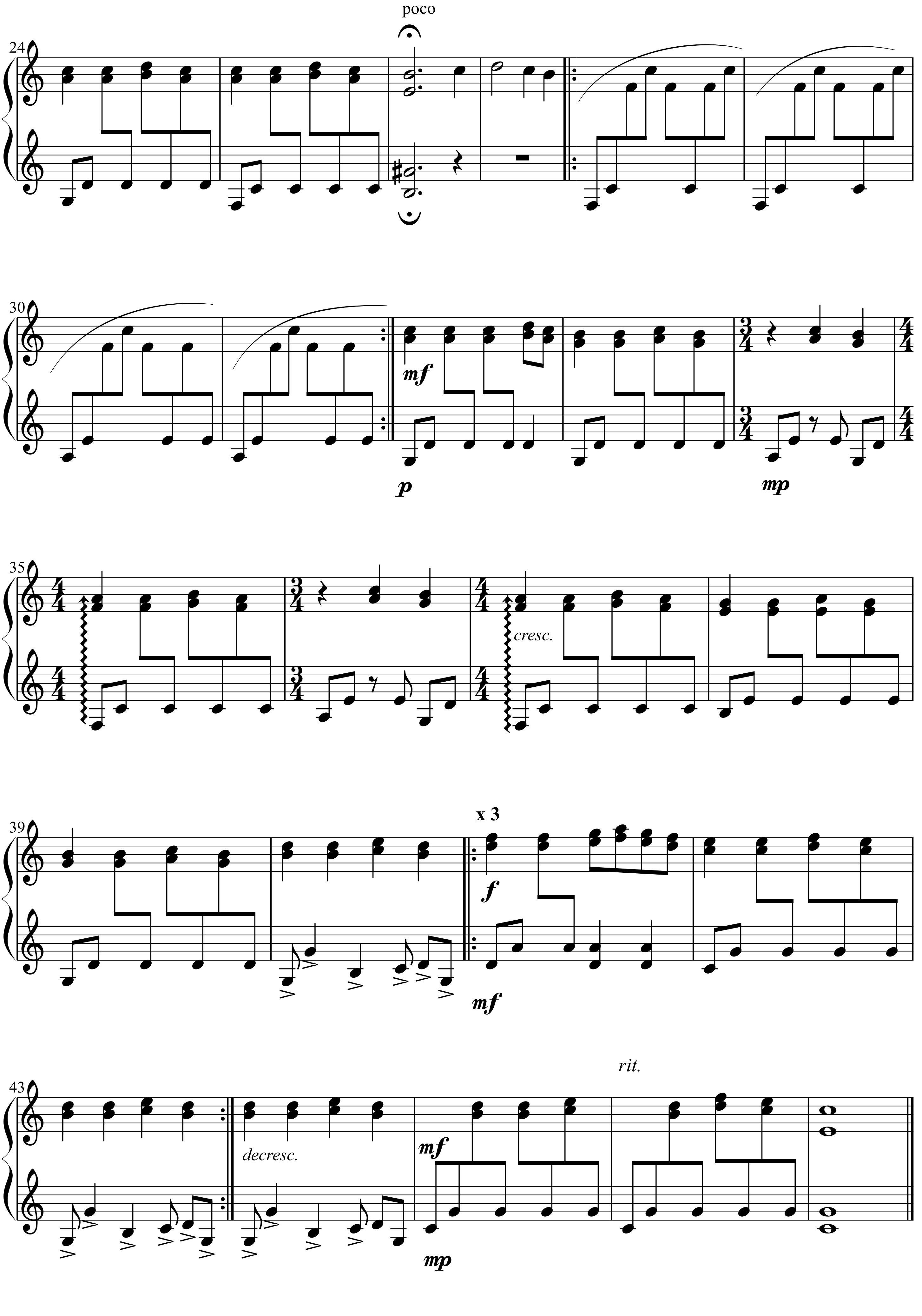
poco
mf
p
mp
cresc.
x 3
f
mf
rit.
decresc.
mf
mp

Estudio nº 2

para vibráfono

Alberto Montero

♩ = 60

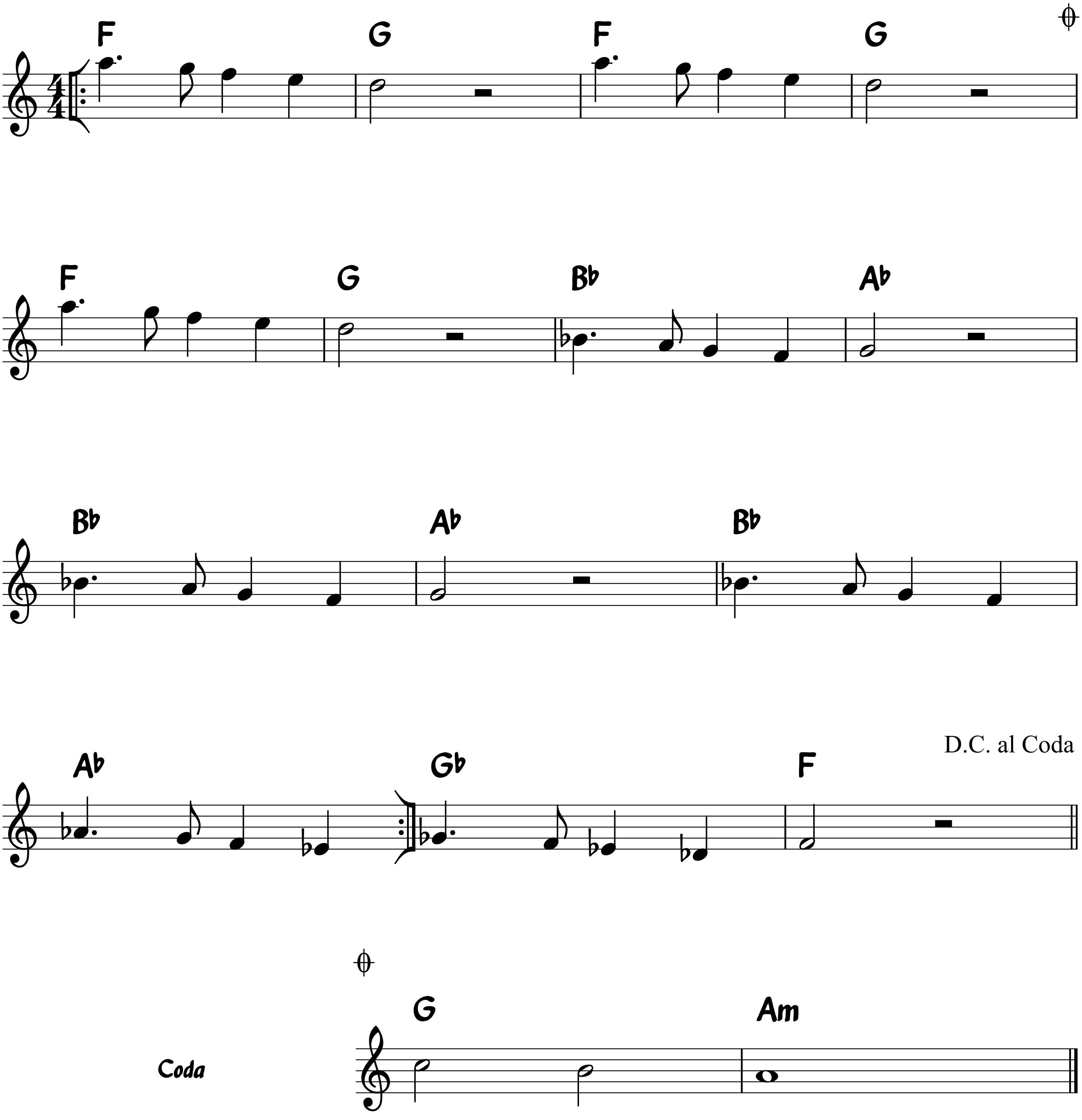

Sagú

Vibráfono

Swing ballad

Larghetto

Alberto Montero

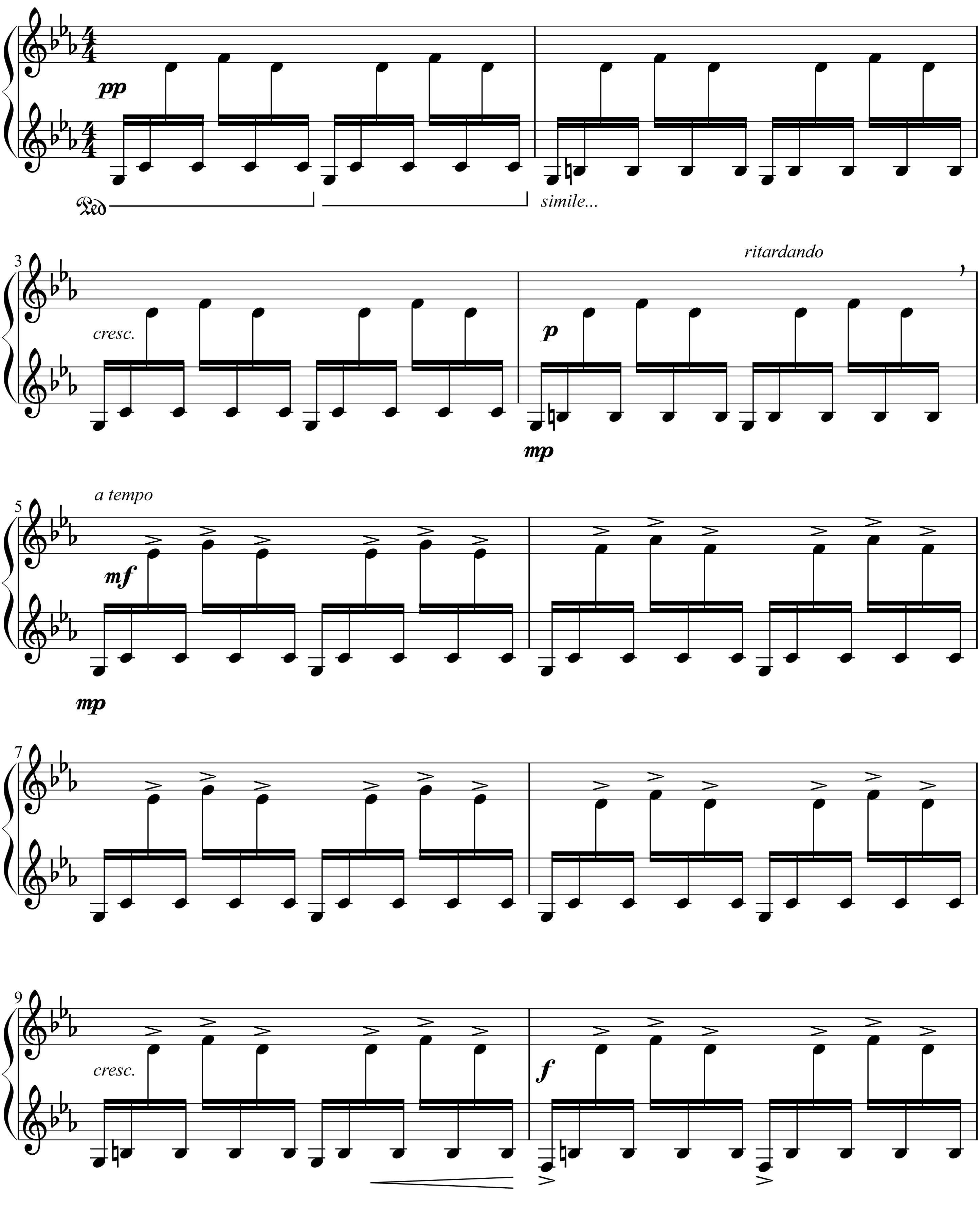

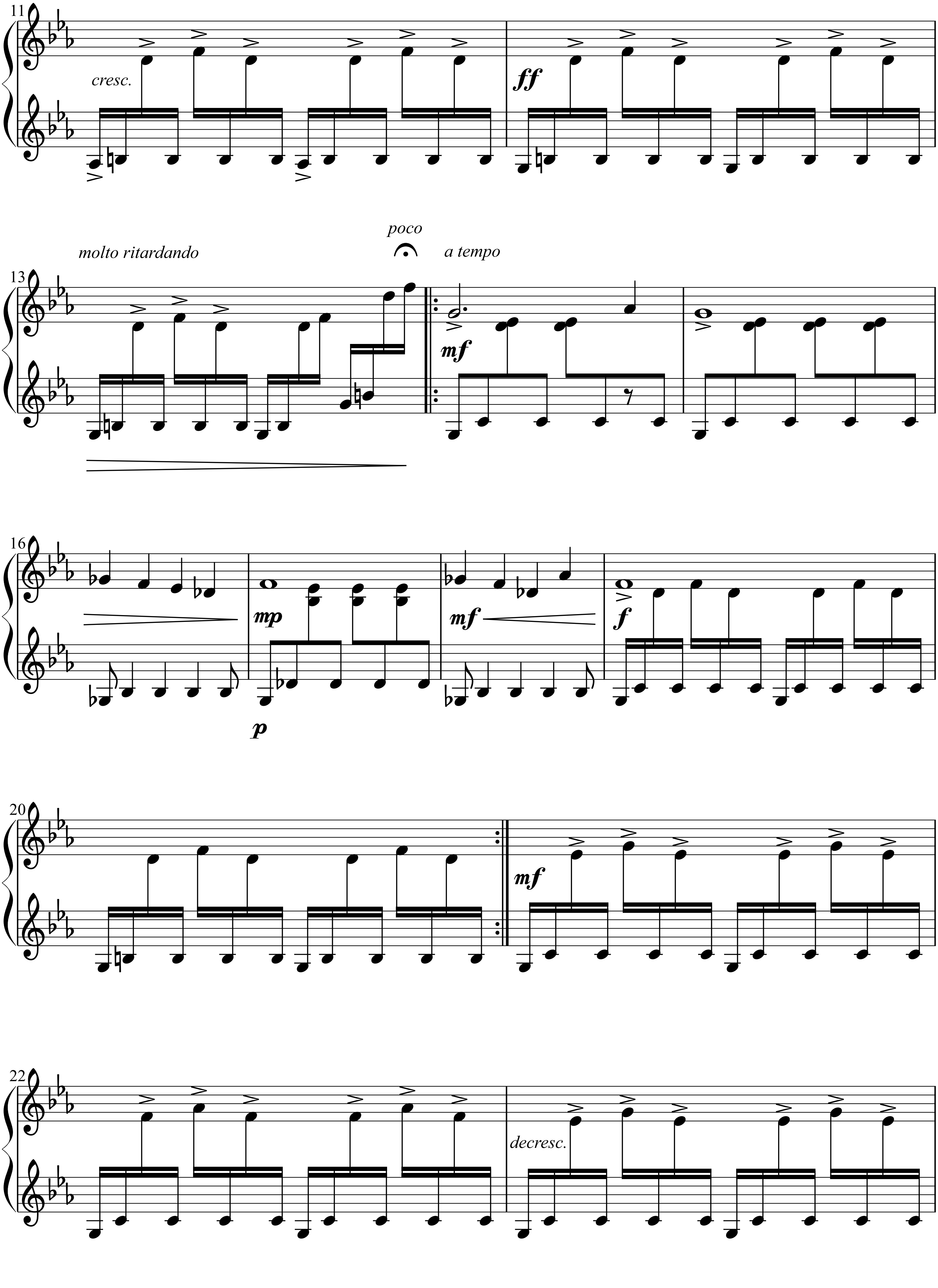
11
cresc.
ff
molto ritardando
poco
a tempo
13
mf
16
mp
p
mf
f
20
mf
22
decresc.

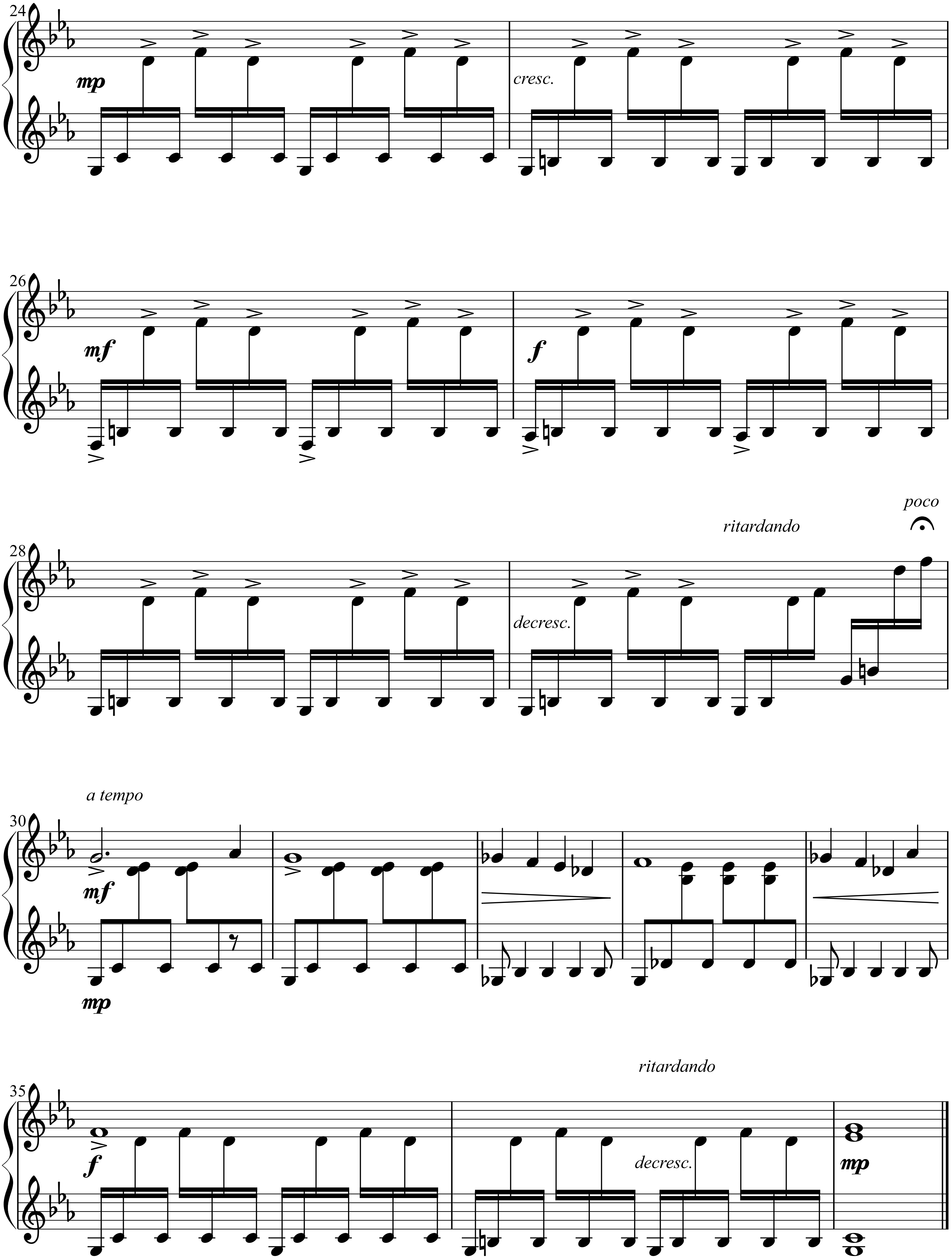
24
mp
cresc.
26
mf
f
poco
ritardando
28
decresc.
a tempo
30
mf
mp
35
ritardando
f
decresc.
mp

Tonfarbe

Alberto Montero

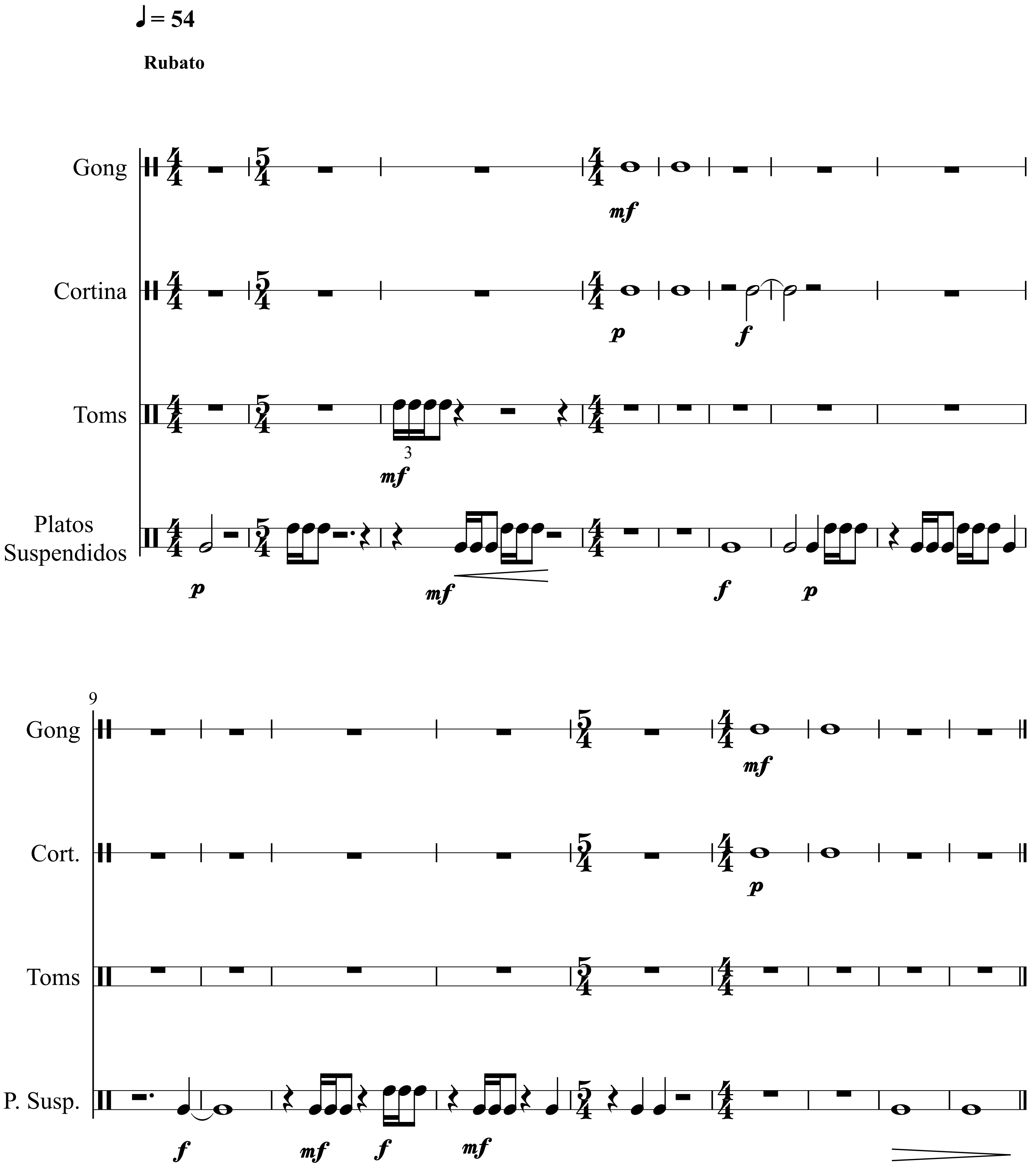

Caibarién

Latin

Alberto Montero

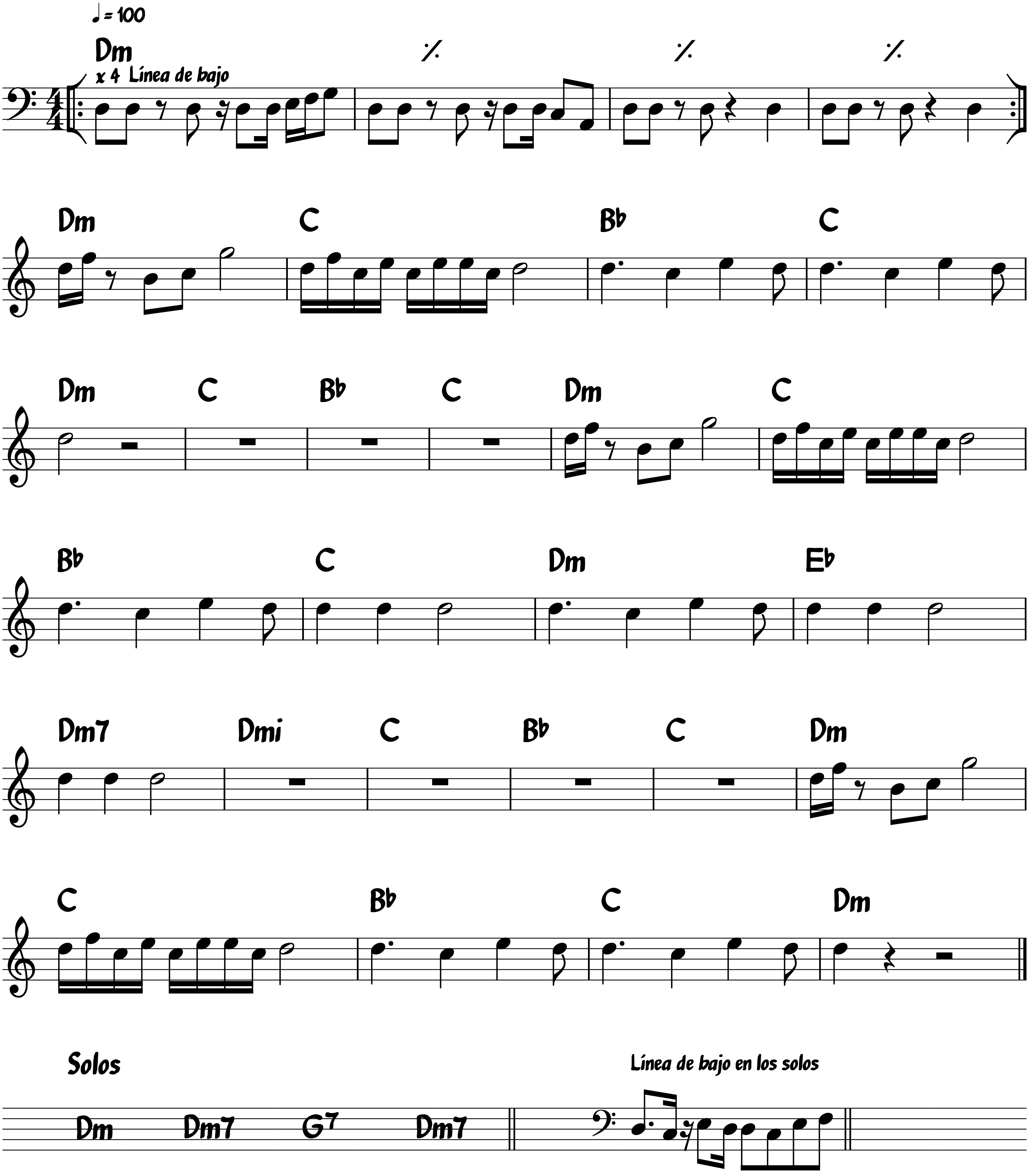

8:00 AM

Alberto Montero

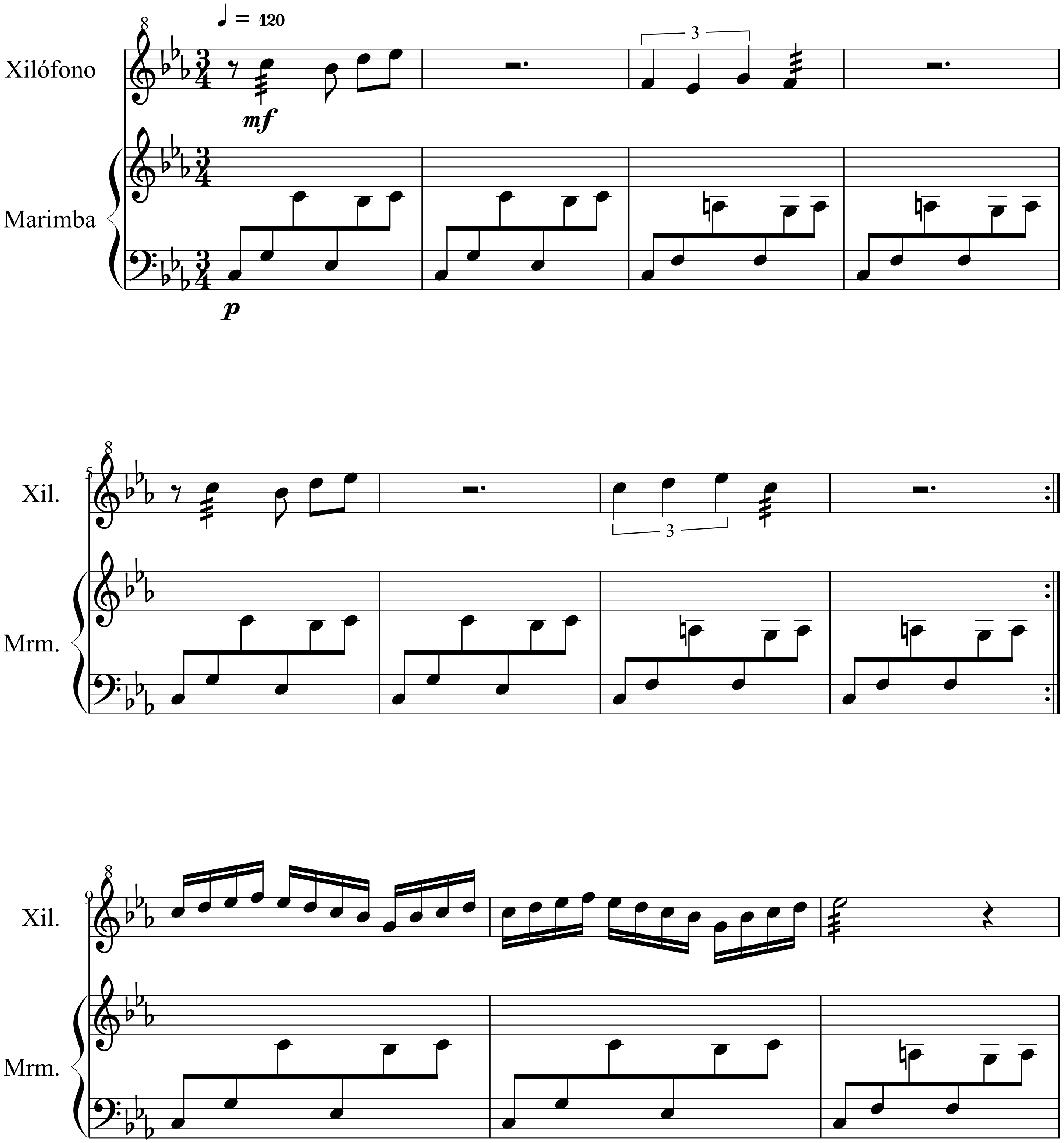

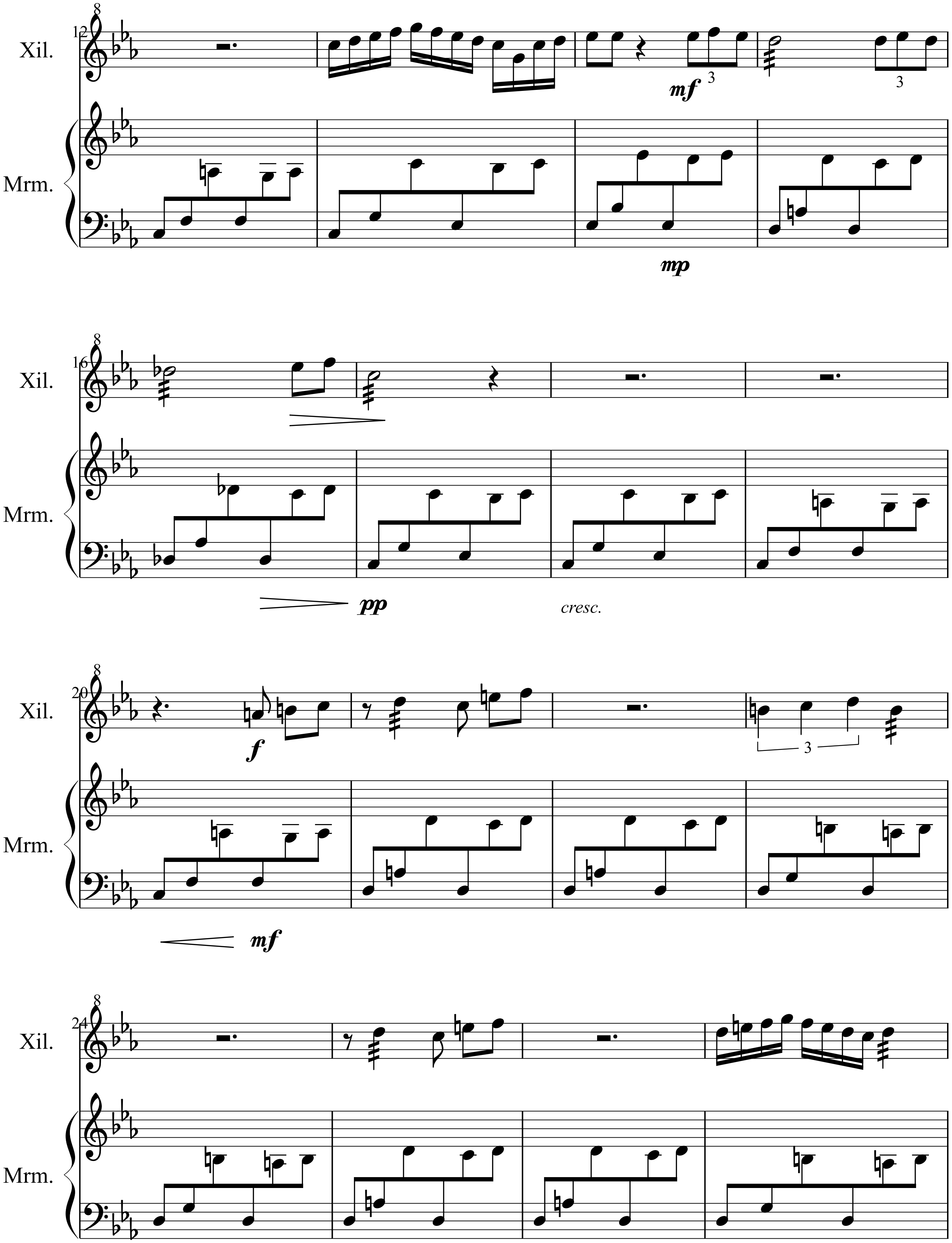
Xil.
Mrm.
12
mf
mp
16
pp
cresc.
20
f
mf
24

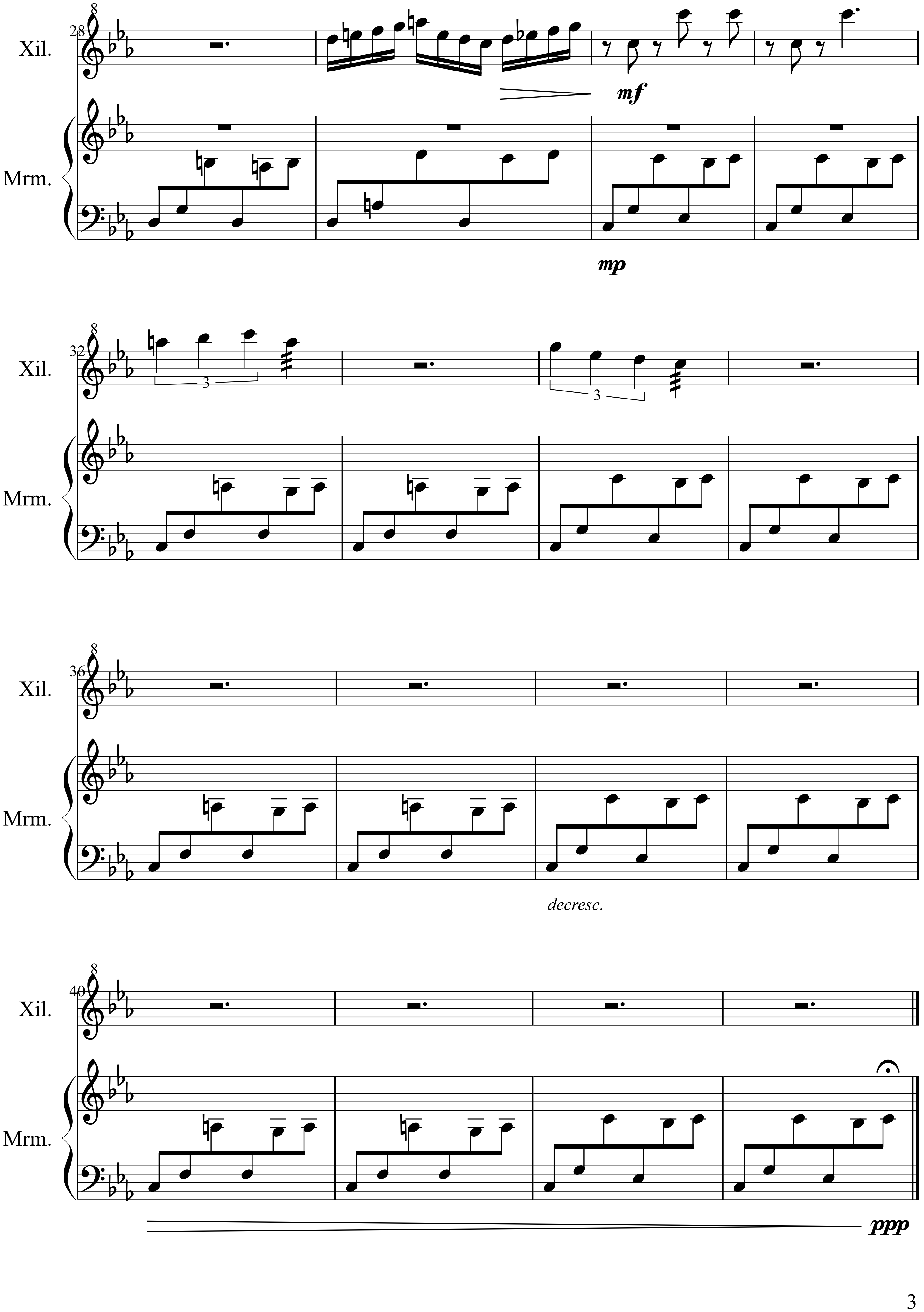
Xil.
Mrm.
28
mf
mp
32
3
36
decresc.
40
ppp

Obstinato Blues

Alberto Montero

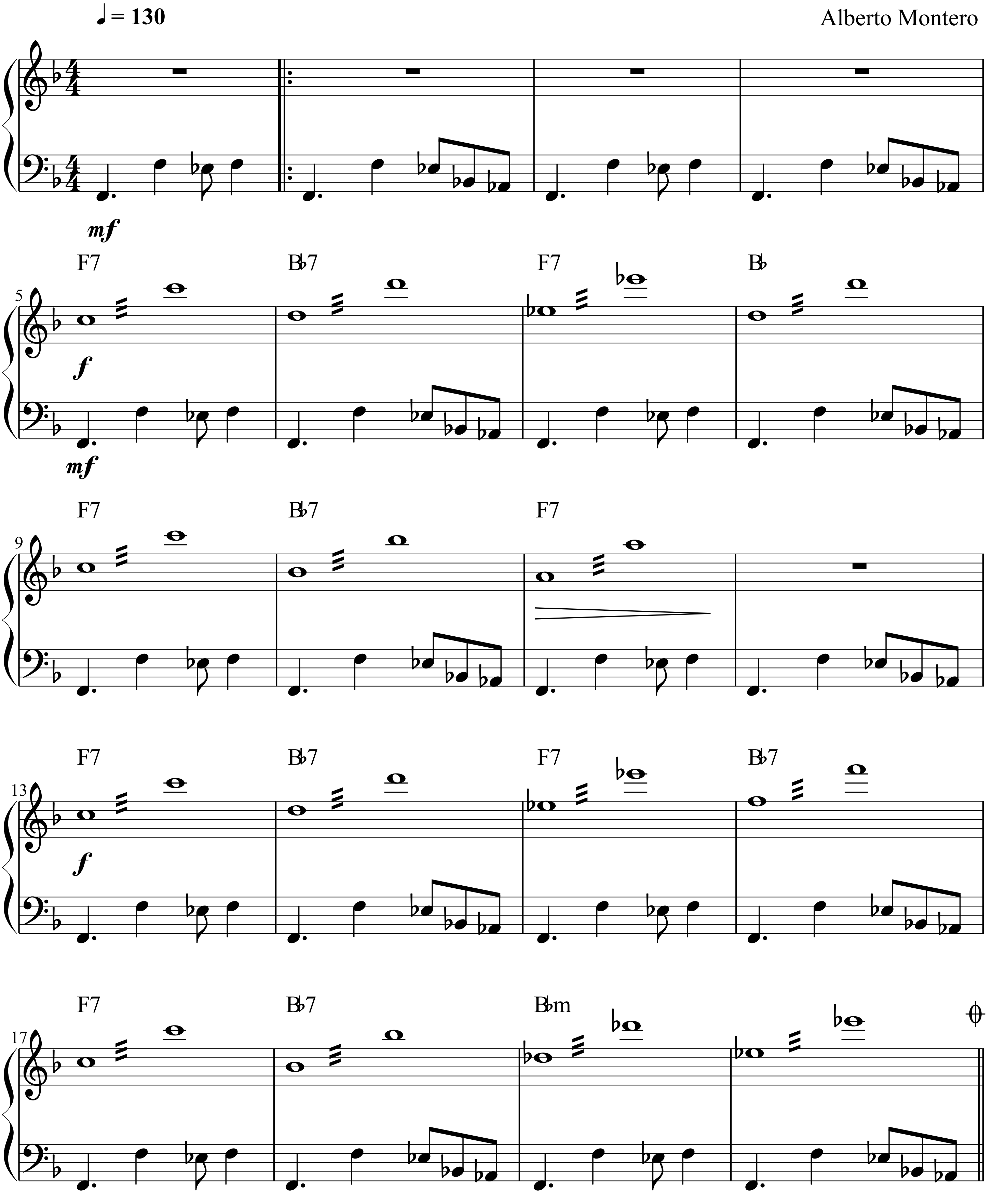

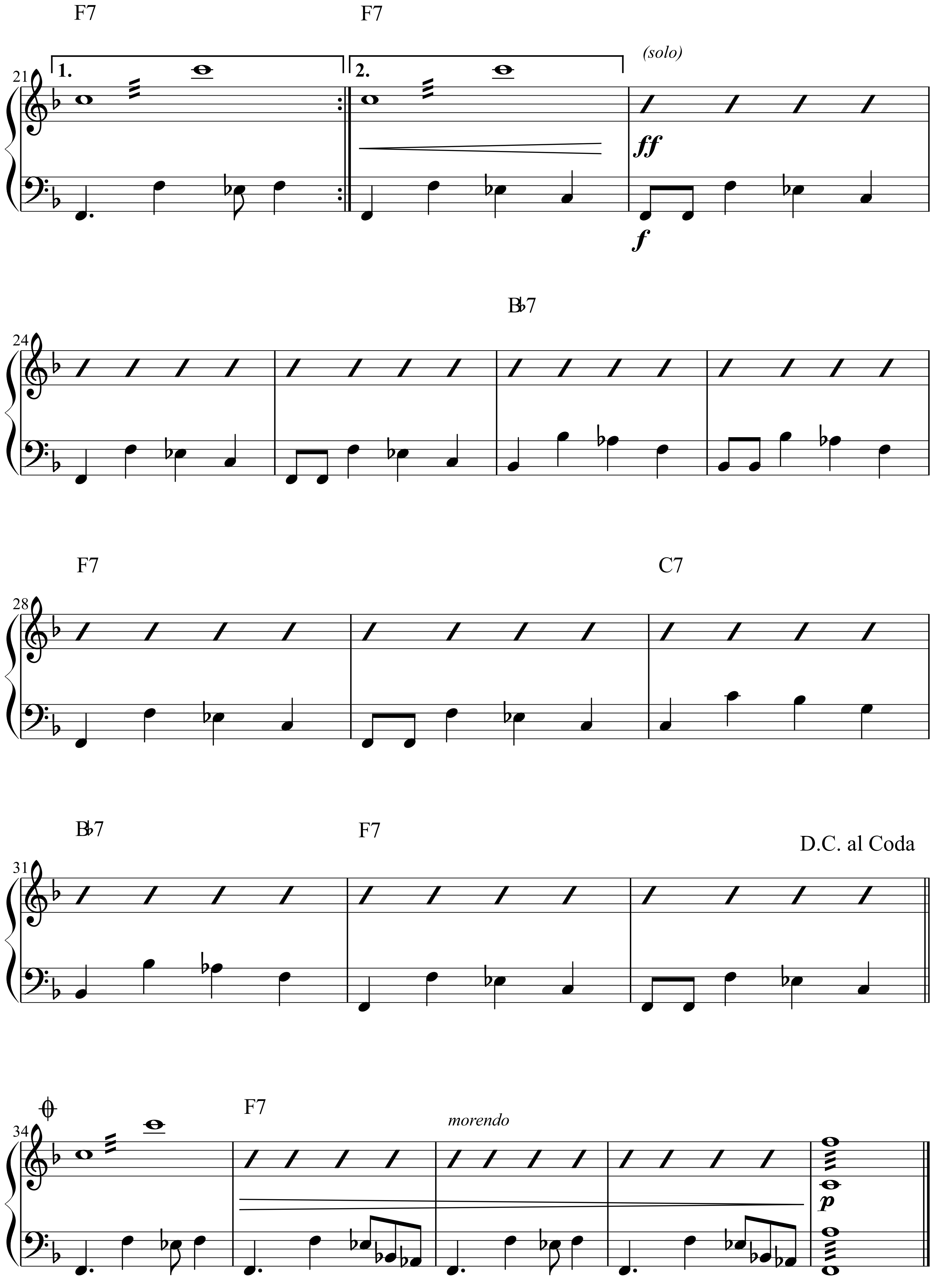
F7
F7
(solo)
21
1.
2.
ff
f
B♭7
24
F7
C7
28
B♭7
F7
D.C. al Coda
31
F7
morendo
34
p

Málaga, 2011

Idris

Pieza breve para percusión

Alberto Montero

NOTACIÓN PARA BATERÍA

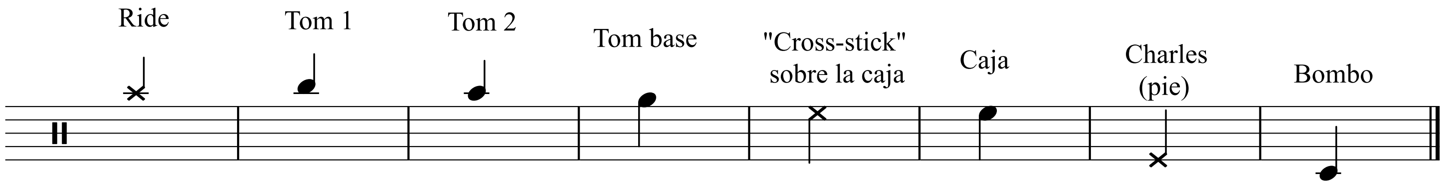

Idris

Alberto Montero

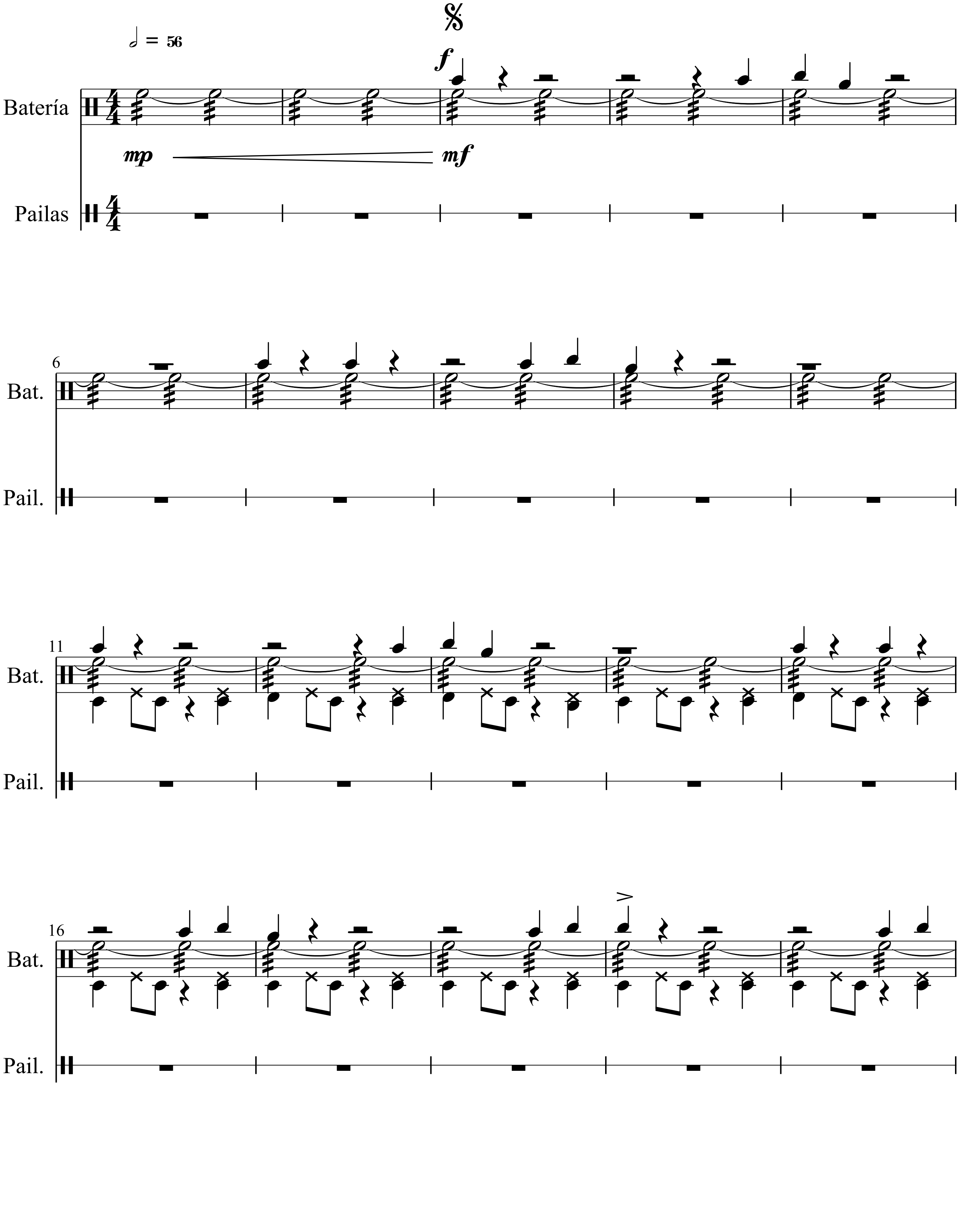

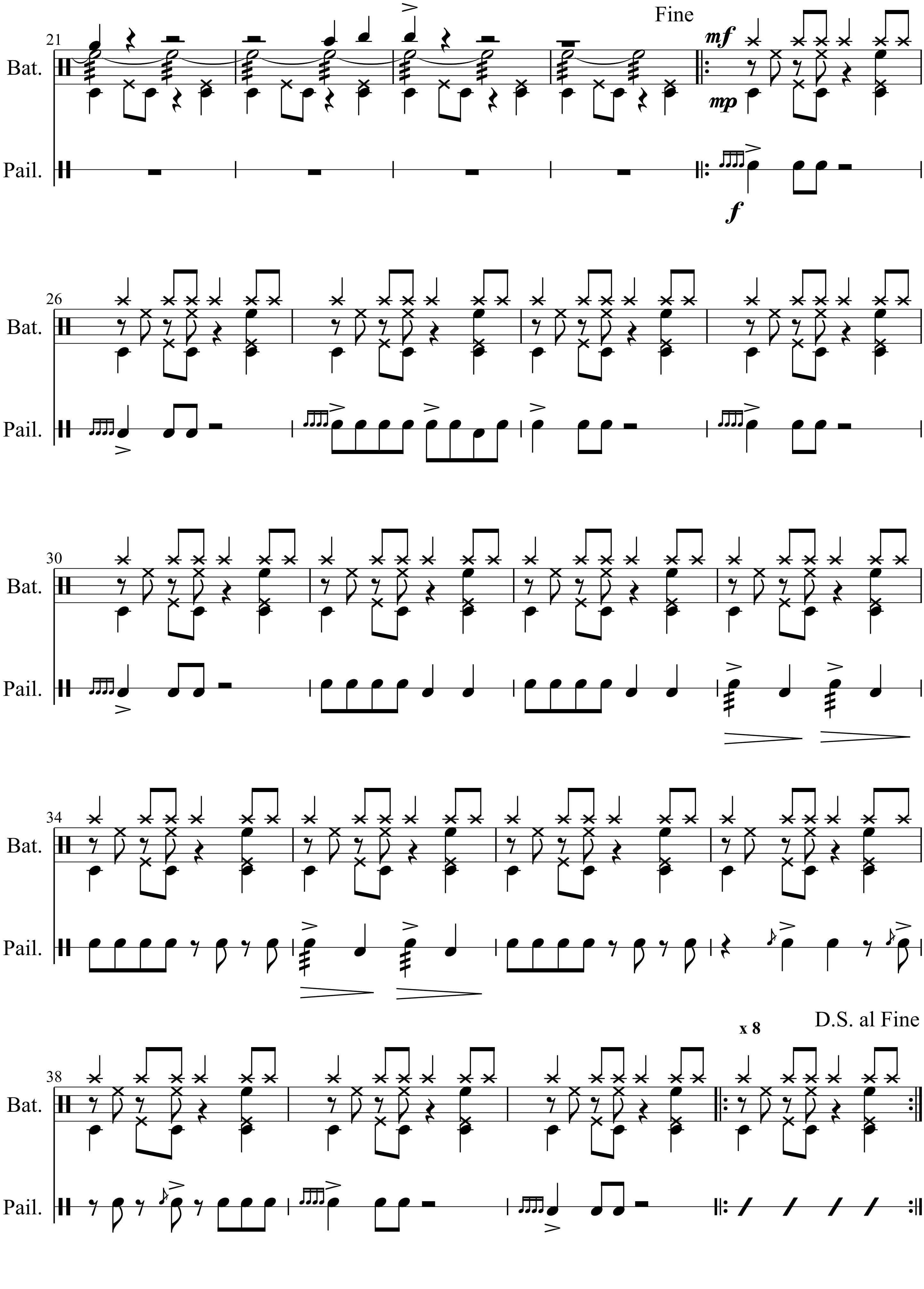
21
Bat.
Pail.
Fine
mf
mp
f
26
30
34
38
x 8
D.S. al Fine

Leyenda

Alberto Montero

Latin

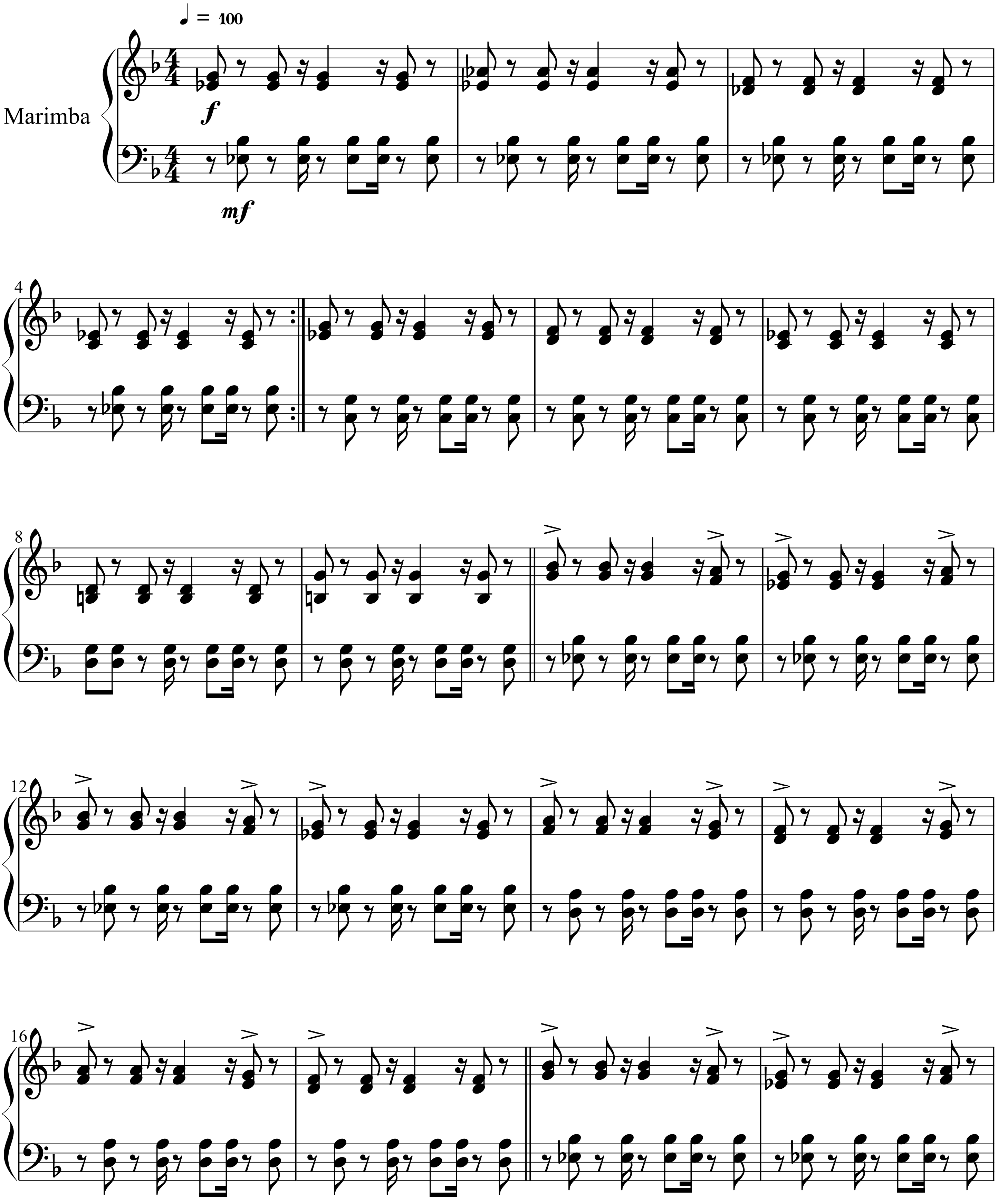

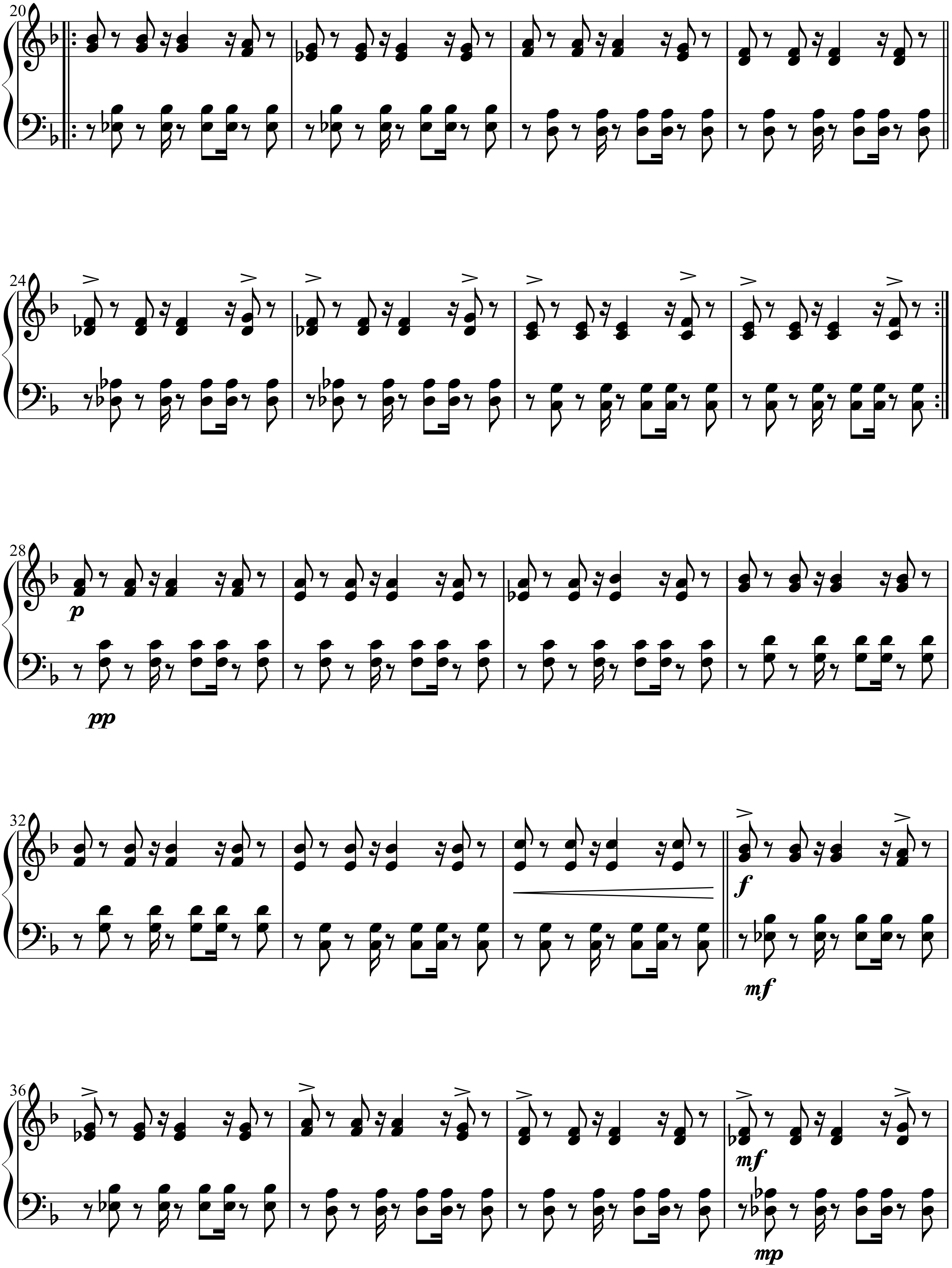
20
24
28
p
pp
32
f
mf
36
mf
mp

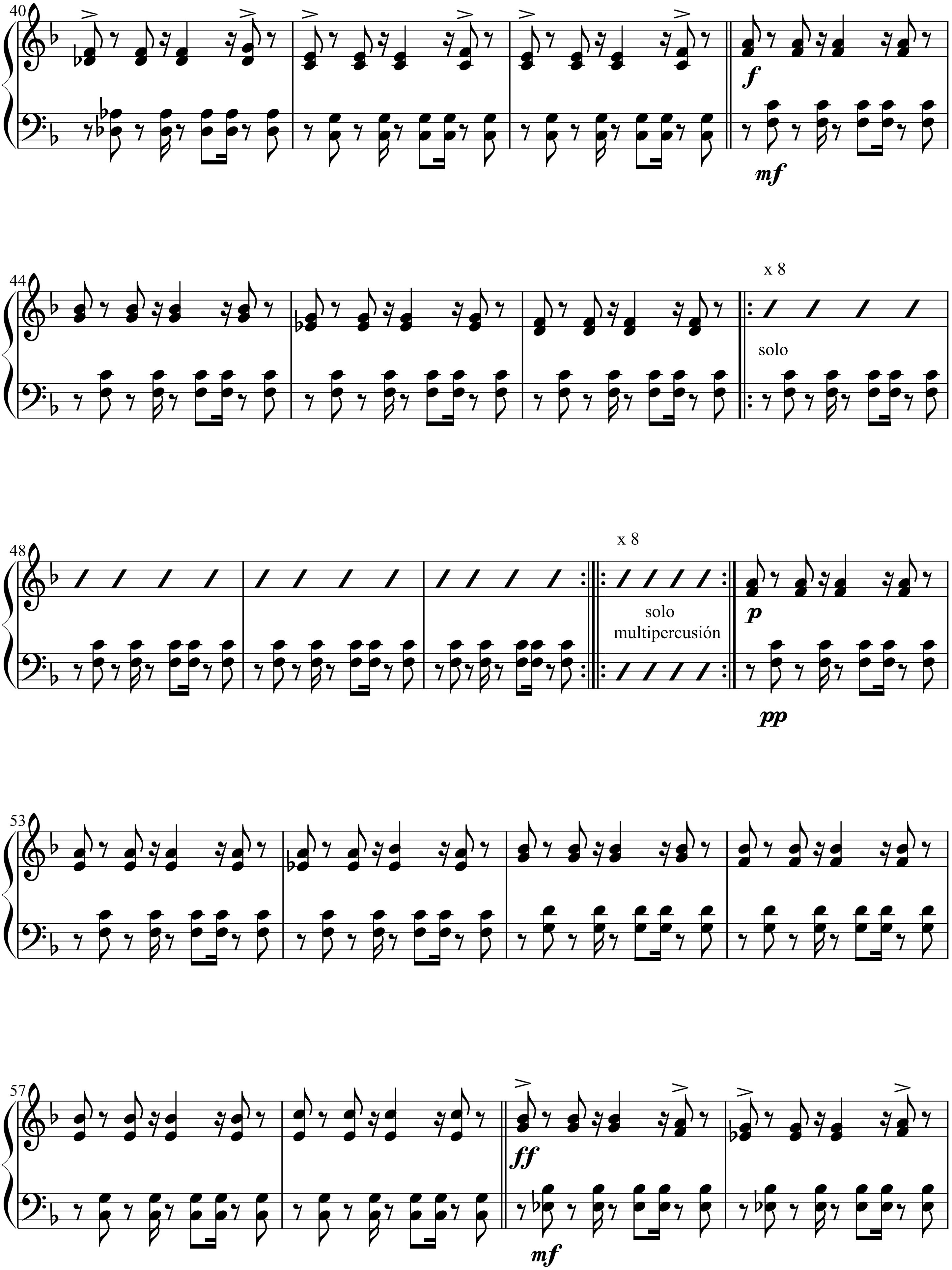
x 8
solo
x 8
solo
multipercusión

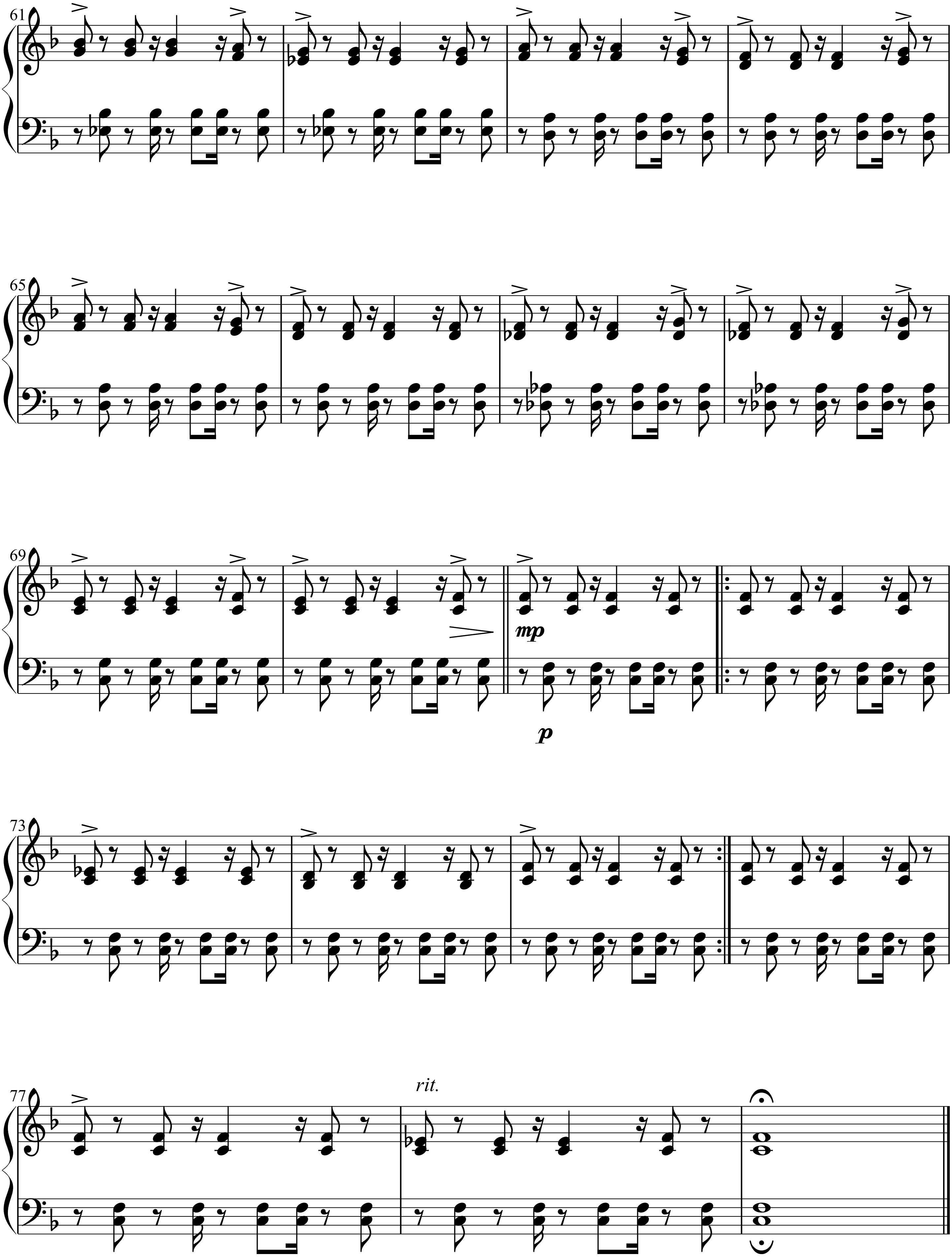
61
65
69
mp
p
73
77
rit.
