# Supplementary material for: Effects of music therapy as an adjunct to chest physiotherapy in children with cystic fibrosis: A randomized controlled trial
Source: PLoS One. 2020 Oct 30;15(10):e0241334. doi: 10.1371/journal.pone.0241334 (PMC7598495; doi:10.1371/journal.pone.0241334)
Supplement: S6 File — Music CD. Because the recorded music was compiled in a physical format, i.e., an audio compact disc, the songs used in this study are available from the corresponding authors. (PDF) [file pone.0241334.s006.pdf]

CUESTIONARIO INICIAL – TODOS LOS GRUPOS

En primer lugar, me gustaría darle las gracias por participar en este estudio.

*Este es un breve cuestionario que nos proporcionará información acerca de su **experiencia en general con la fisioterapia respiratoria**. Su nombre no se relacionará con sus respuestas y sus respuestas serán totalmente confidenciales.*

*Es importante que usted **responda con honestidad. No hay respuestas "correctas",** por lo que no sienta que debe responder de una manera particular.*

1. Información Demográfica

Me gustaría comenzar preguntándole por su edad/ edad de su hijo/a: [ ]

2. Fisioterapia respiratoria diaria

Le voy a pedir que **piense en el último mes** y me responda según su impresión global en este tiempo.

a) ¿Con qué frecuencia realiza/ su hijo o hija la fisioterapia respiratoria?

Nunca/casi nunca		A veces/de vez en cuando		Con asiduidad		Siempre/casi S.	

b) ¿Cuántas veces al día?

1		2	

c) ¿Suele hacerla sin muchas interrupciones?

si		no	

d) ¿Cuánto tiempo dura la rutina de fisioterapia? [ ]

e) ¿Quién suele hacer la rutina?

Usted		Otro familiar	

f) ¿Suele complementar la rutina de fisioterapia respiratoria con algo?

si		no	

Música		Radio/TV		Cantando		Cuento		Juguetes		Otros	

3. Variables percepción-actitud

a) En la escala de "**-3 a +3**" que tiene delante, escoja el valor que considere más apropiado para definir como su hijo/hija responde a la fisioterapia [ ]

**-3**	puntuación mínima	muy desagradable (sentimiento negativo)
**0**	puntuación neutra	ni negativo ni positivo
**+3**	puntuación máxima	muy agradable (sentimiento positivo)

b) En la escala de "**-3 a +3**" que tiene delante, califique su propia respuesta a la fisioterapia

[ ]

c) De la tabla "**percepción**", que palabras describen mejor sus sentimientos hacia la fisioterapia. **Elija 3 palabras:**

resignación	relajación	afecto	nada
aburrida	aceptable	encantadora	triste
tolerable	molesta	cansada	divertida

d) De la lista **"actitud hacia la fisioterapia respiratoria"**, que palabras describirían mejor la actitud de su hijo/hija hacia la fisioterapia. **Elija 3 palabras:**

la detesta	conforme	aburrido	divertido
desagradable-antipatía	aprecio por el beneficio	aversión	satisfacción
voluntarioso	le angustia	la acepta	tolerable

e) ¿Le resulta relajante / a su hijo o hija realizar la fisioterapia respiratoria?

si		no	

f) Piense en el tiempo que necesita para realizar la fisioterapia.

¿Se le hace larga?

si		no	

Mire la flecha "**percepción temporal**". ¿Cuánto tiempo le parece que necesita para completarla?

10 min o menos		20 min		30 min		45 min		60 min o más	

Muchas gracias por responder estas preguntas. Su tiempo es muy apreciado.

Grupo control: *En seis semanas le llamaré para hacer el segundo cuestionario por teléfono. Es completamente opcional.*

si		no	

Grupo placebo: *En seis semanas le llamaré para hacer el segundo cuestionario por teléfono. En la página web tiene mi número de teléfono y dirección e-mail para cualquier duda que le surja al respecto. Es completamente opcional.*

si		no	

Grupo tratado*: En la página web tiene mi número de teléfono y dirección e-mail para cualquier duda que le surja al respecto o si tiene algún problema con la reproducción del CD de música. En seis semanas me pondré de nuevo en contacto con usted para realizar un segundo cuestionario. Es completamente opcional.*

si		no	

CUESTIONARIO INTERMEDIO – TODOS LOS GRUPOS

Este es nuestro segundo cuestionario. De nuevo, me gustaría darle las gracias por participar en este estudio. Voy a hacerle algunas preguntas para el seguimiento del cuestionario inicial. Su nombre no se relacionará con sus respuestas y sus respuestas serán totalmente confidenciales.

Es importante que usted ***responda con honestidad. No hay respuestas "correctas",*** *por lo que no sienta que debe responder de una manera particular.*

1. Fisioterapia respiratoria diaria

Le voy a pedir que **piense en el último mes** y me responda según su impresión global en este tiempo.

a) ¿Ha continuado/ su hijo o hija realizando la fisioterapia ______________ veces al día?

b) ¿Continúa haciendo la rutina?

Usted		Otro familiar	

c) ¿Ha habido cambios en la forma de hacer la fisioterapia durante las últimas seis semanas?

(p.ej: sigue patrones similares, las mismas actividades…)

____

si		no	

d) ¿Sigue complementando la ruina de fisioterapia con ______________?

Muchas gracias por responder estas preguntas. Su tiempo es muy apreciado.

Grupo control: *En seis semanas le llamaré para hacer el cuestionario final por teléfono y en ese momento, si lo desea, recibirá el CD de música a utilizar como complemento de la fisioterapia respiratoria diaria. Es completamente opcional.*

si		no	

Grupo placebo: *En seis semanas le llamaré para hacer el cuestionario final por teléfono y en ese momento, si lo desea, recibirá el CD de música a utilizar como complemento de la fisioterapia respiratoria diaria. Es completamente opcional.*

si		no	

Grupo tratado*: En seis semanas le llamaré para hacer el cuestionario final por teléfono.* Es completamente opcional.

si		no	

CUESTIONARIO FINAL – GRUPO CONTROL

Este es nuestro cuestionario final. De nuevo, me gustaría darle las gracias por participar en este estudio. Voy a hacerle algunas preguntas para el seguimiento del cuestionario inicial y del segundo cuestionario. Su nombre no se relacionará con sus respuestas y sus respuestas serán totalmente confidenciales.

*Es importante que usted **responda con honestidad. No hay respuestas "correctas",** por lo que no sienta que debe responder de una manera particular.*

1. Fisioterapia respiratoria diaria

Le voy a pedir que **piense en el último mes** y me responda según su impresión global en este tiempo.

a) ¿Ha continuado/ su hijo o hija realizando la fisioterapia ______________ veces al día?

b) ¿Cuánto tiempo dura la rutina de fisioterapia? [ ]

c) ¿Continúa haciendo la rutina?

Usted		Otro familiar	

d) ¿Ha habido cambios en la forma de hacer la fisioterapia durante las últimas seis semanas?

(p.ej: sigue patrones similares, las mismas actividades…)

____

si		no	

e) ¿Sigue complementando la ruina de fisioterapia con ______________?

2. Variables percepción-actitud

a) En la escala de "**-3 a +3**" que tiene delante, escoja el valor que considere más apropiado para definir como su hijo/hija responde a la fisioterapia [ ]

**-3**	puntuación mínima	muy desagradable (sentimiento negativo)
**0**	puntuación neutra	ni negativo ni positivo
**+3**	puntuación máxima	muy agradable (sentimiento positivo)

b) En la escala de "**-3 a +3**" que tiene delante, califique su propia respuesta a la fisioterapia

[ ]

c) De la tabla "**percepción**", que palabras describen mejor sus sentimientos hacia la fisioterapia. **Elija 3 palabras:**

resignación	relajación	afecto	nada
aburrida	aceptable	encantadora	triste
tolerable	molesta	cansada	divertida

d) De la lista **<u>"actitud hacia la fisioterapia respiratoria"</u>**, que palabras describirían mejor la actitud de su hijo/hija hacia la fisioterapia. **<u>Elija 3 palabras:</u>**

la detesta	conforme	aburrido	divertido
desagradable-antipatía	aprecio por el beneficio	aversión	satisfacción
voluntarioso	le angustia	la acepta	tolerable

e) ¿Le resulta relajante / a su hijo o hija realizar la fisioterapia respiratoria?

si		no	

f) Piense en el tiempo que necesita para realizar la fisioterapia.

¿Se le hace larga?

si		no	

Mire la flecha "**<u>percepción temporal</u>**". ¿Cuánto tiempo le parece que necesita para completarla?

10 min o menos		20 min		30 min		45 min		60 min o más	

Muchas gracias por responder estas preguntas. Su tiempo es muy apreciado. Si lo desea, puede utilizar el CD de música que le proporcionamos como complemento de la fisioterapia respiratoria diaria. En la web tiene mi número de teléfono y dirección e-mail para cualquier duda que le surja al respecto o si tiene algún problema con la reproducción de la música.

si		no	

CUESTIONARIO FINAL- GRUPO PLACEBO

Este es nuestro cuestionario final. De nuevo, me gustaría darle las gracias por participar en este estudio. Voy a hacerle algunas preguntas para el seguimiento del cuestionario inicial y del segundo cuestionario. Su nombre no se relacionará con sus respuestas y sus respuestas serán totalmente confidenciales.

*Es importante que usted **<u>responda con honestidad. No hay respuestas "correctas",</u>** por lo que no sienta que debe responder de una manera particular.*

1. Fisioterapia respiratoria diaria

Le voy a pedir que **<u>piense en el último mes</u>** y me responda según su impresión global en este tiempo.

a) ¿Ha continuado/ su hijo o hija realizando la fisioterapia _______________ veces al día?

b) ¿Cuánto tiempo dura la rutina de fisioterapia?

c) ¿Continúa haciendo la rutina?

Usted		Otro familiar	

d) ¿Ha habido cambios en la forma de hacer la fisioterapia durante las últimas seis semanas?

(p.ej: sigue patrones similares, las mismas actividades…)

____

si		no	

e) ¿Sigue complementando la ruina de fisioterapia con ______________?

2. Variables percepción-actitud

a) En la escala de "**-3 a +3**" que tiene delante, escoja el valor que considere más apropiado para definir como su hijo/hija responde a la fisioterapia [ ]

**-3**	puntuación mínima	muy desagradable (sentimiento negativo)
**0**	puntuación neutra	ni negativo ni positivo
**+3**	puntuación máxima	muy agradable (sentimiento positivo)

b) En la escala de "**-3 a +3**" que tiene delante, califique su propia respuesta a la fisioterapia

[ ]

c) De la tabla "**percepción**", que palabras describen mejor sus sentimientos hacia la fisioterapia. **Elija 3 palabras:**

resignación	relajación	afecto	nada
aburrida	aceptable	encantadora	triste
tolerable	molesta	cansada	divertida

d) De la lista **"actitud hacia la fisioterapia respiratoria"**, que palabras describirían mejor la actitud de su hijo/hija hacia la fisioterapia. **Elija 3 palabras:**

la detesta	conforme	aburrido	divertido
desagradable-antipatía	aprecio por el beneficio	aversión	satisfacción
voluntarioso	le angustia	la acepta	tolerable

e) ¿Le resulta relajante / a su hijo o hija realizar la fisioterapia respiratoria?

si		no	

f) Piense en el tiempo que necesita para realizar la fisioterapia.

¿Se le hace larga?

si		no	

Mire la flecha "**percepción temporal**". ¿Cuánto tiempo le parece que necesita para completarla?

10 min o menos		20 min		30 min		45 min		60 min o más	

3. Sobre el uso de música

a) ¿Con qué frecuencia ha utilizado música?

Nunca/casi nunca		A veces/de vez en cuando		Con asiduidad		Siempre/casi S.	

b) Ahora que usted ha utilizado música durante 6 semanas, ¿hay algún comentario que le gustaría hacer sobre cualquier aspecto de la música?

c) En la escala de "**-3 a +3**" que tiene delante, escoja el valor que considere más apropiado para definir como su hijo/hija responde a la utilización de música durante la fisioterapia

**-3**	puntuación mínima	muy desagradable (sentimiento negativo)
**0**	puntuación neutra	ni negativo ni positivo
**+3**	puntuación máxima	muy agradable (sentimiento positivo)

d) En la escala de "**-3 a +3**" que tiene delante, califique su propia respuesta a la utilización de música durante la fisioterapia

e) ¿Ha sido útil la música?

si		no	

f) ¿De qué manera ha sido útil/ no útil?

g) ¿Le recomendaría música a otros padres/pacientes durante la fisioterapia?

si		no	

h) ¿Le gustaría continuar con el uso de música durante la fisioterapia?

si		no	

i) ¿Con qué prefiere complementar en el futuro la fisioterapia respiratoria?

Muchas gracias por responder estas preguntas. Su tiempo es muy apreciado. Si lo desea, puede utilizar el CD de música que le proporcionamos como complemento de la fisioterapia respiratoria diaria. En la web tiene mi número de teléfono y dirección e-mail para cualquier duda que le surja al respecto o si tiene algún problema con la reproducción de la música.

si		no	

CUESTIONARIO FINAL- GRUPO TRATADO

Este es nuestro cuestionario final. De nuevo, me gustaría darle las gracias por participar en este estudio. Voy a hacerle algunas preguntas para el seguimiento del cuestionario inicial y del segundo cuestionario. Su nombre no se relacionará con sus respuestas y sus respuestas serán totalmente confidenciales.

*Es importante que usted **responda con honestidad. No hay respuestas "correctas",** por lo que no sienta que debe responder de una manera particular.*

1. Fisioterapia respiratoria diaria

Le voy a pedir que **piense en el último mes** y me responda según su impresión global en este tiempo.

a) ¿Ha continuado/ su hijo o hija realizando la fisioterapia ______________ veces al día?

b) ¿Cuánto tiempo dura la rutina de fisioterapia? [ ]

c) ¿Continúa haciendo la rutina?

usted		Otro familiar	

d) ¿Ha habido cambios en la forma de hacer la fisioterapia durante las últimas seis semanas?

(p.ej: sigue patrones similares, las mismas actividades…)

____

si		no	

e) ¿Sigue complementando la ruina de fisioterapia con ______________?

2. Variables percepción-actitud

a) En la escala de "**-3 a +3**" que tiene delante, escoja el valor que considere más apropiado para definir como su hijo/hija responde a la fisioterapia [ ]

**-3**	puntuación mínima	muy desagradable (sentimiento negativo)
**0**	puntuación neutra	ni negativo ni positivo
**+3**	puntuación máxima	muy agradable (sentimiento positivo)

b) En la escala de "**-3 a +3**" que tiene delante, califique su propia respuesta a la fisioterapia [ ]

c) De la tabla "**percepción**", que palabras describen mejor sus sentimientos hacia la fisioterapia. **Elija 3 palabras:**

resignación	relajación	afecto	nada
aburrida	aceptable	encantadora	triste
tolerable	molesta	cansada	divertida

d) De la lista **"actitud hacia la fisioterapia respiratoria"**, que palabras describirían mejor la actitud de su hijo/hija hacia la fisioterapia. **Elija 3 palabras:**

la detesta	conforme	aburrido	divertido
desagradable-antipatía	aprecio por el beneficio	aversión	satisfacción
voluntarioso	le angustia	la acepta	tolerable

e) ¿Le resulta relajante / a su hijo o hija realizar la fisioterapia respiratoria?

si		no	

f) Piense en el tiempo que necesita para realizar la fisioterapia.

¿Se le hace larga?

si		no	

Mire la flecha "**percepción temporal**". ¿Cuánto tiempo le parece que necesita para completarla?

10 min o menos		20 min		30 min		45 min		60 min o más	

3. Sobre el CD de música

a) ¿Con qué frecuencia ha utilizado el CD de música?

Nunca/casi nunca		A veces/de vez en cuando		Con asiduidad		Siempre/casi S.	

b) Ahora que usted ha utilizado el CD de música durante 6 semanas, ¿hay algún comentario que le gustaría hacer sobre cualquier aspecto de la música?

c) En la escala de "**-3 a +3"** que tiene delante, escoja el valor que considere más apropiado para definir como su hijo/hija responde a la utilización del CD de música durante la fisioterapia

**-3**	puntuación mínima	muy desagradable (sentimiento negativo)
**0**	puntuación neutra	ni negativo ni positivo
**+3**	puntuación máxima	muy agradable (sentimiento positivo)

d) En la escala de "**-3 a +3"** que tiene delante, califique su propia respuesta a la utilización del CD de música durante la fisioterapia

e) ¿Ha sido útil el CD de música?

si		no	

f) ¿De qué manera ha sido útil/ no útil?

g) ¿Hay alguna de las 3 partes de la música en particular que le haya sido o piense que puede ser más útil?

1		2		3	

¿Por qué?

h) ¿Le recomendaría este CD de música a otros padres/pacientes?

si		no	

i) ¿Le gustaría continuar con el uso de esta u otra música terapéutica durante la fisioterapia?

si		no	

j) ¿Con qué prefiere complementar en el futuro la fisioterapia respiratoria?

Muchas gracias por responder estas preguntas. Su tiempo es muy apreciado.
